# Colombiains A–C, Three New Alkaloids From the Soft Coral‐Derived Fungus *Cladosporium colombiae* CSGL 10M10

**DOI:** 10.1002/cbdv.71769

**Published:** 2026-09-30

**Authors:** Jing Xie, Bing‐Bing Liu, Yang‐Ying Zheng, Jun‐Feng Huan, Xiao‐Yu Tian, Xiu‐Ping Lin, Hui‐Mei Xie, Yuan‐Yuan Cheng, Cui‐Xian Zhang

**Affiliations:** ^1^ School of Pharmaceutical Sciences Guangzhou University of Chinese Medicine Guangzhou Guangdong P. R. China; ^2^ State Key Laboratory of Tropical Oceanography/Guangdong Key Laboratory of Marine Materia Medica South China Sea Institute of Oceanology Chinese Academy of Sciences Guangzhou P. R. China

**Keywords:** alkaloids, cardioprotective activity, *Cladosporium colombiae*

## Abstract

Three new alkaloids, colombiains **A**–**C** (**1**–**3**), together with 8 known ones (**4**–**11**), were isolated from the soft coral‐derived fungus *Cladosporium colombiae* CSGL 10M10. Their structures were elucidated by spectroscopic interpretation, electronic circular dichroism (ECD), and single‐crystal X‐ray crystallographic methods. Compounds **6** and **11** exhibited significant cardioprotective activity against oxygen‐glucose deprivation/reoxygenation (OGD/R)‐induced injury in H9c2 myocardial cell line at 10 µM. In addition, a preliminary network pharmacology analysis of compound **6** suggested that *β*‐carboline alkaloids might exert protective effects through a multi‐target, multi‐pathway mode of action, providing a rationale for future mechanistic studies.

## Graphical abstract

Three new alkaloids, colombiains **A**–**C** (**1**–**3**), together with eight known ones (**4**–**11**) were isolated from the soft coral‐derived fungus *Cladosporium colombiae* CSGL 10M10. Compounds **6** and **11** exhibited significant cardioprotective activity against OGD/R‐induced injury in H9c2 cardiomyocytes at 10 µM. Network pharmacology analysis of compound **6** suggested a multi‐target, multi‐pathway protective mechanism.

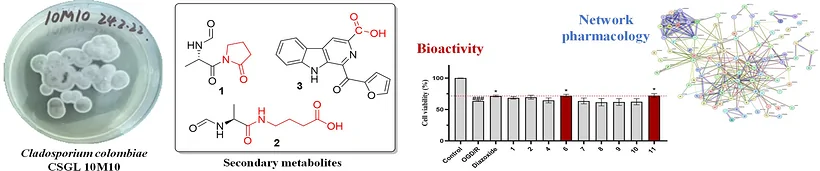

## 1 Introduction

*Cladosporium*, a genus of saprophytic or endophytic fungi, is widely distributed in natural environments and particularly abundant in marine ecosystems has garnered significant interest for its chemically diverse and bioactive secondary metabolites, which encompass polyketides, alkaloids, steroids, and terpenoids with antimicrobial, cytotoxic, and enzyme‐inhibitory effects [1, 2, 3]. The alkaloids reported from *Cladosporium* are mainly tetramic acid derivatives, diketopiperazines, and other types such as *β*‐carboline alkaloids [2]. Notably, only one *β*‐carboline alkaloid has been isolated from this genus [4], suggesting that *Cladosporium* represents a promising yet underexplored source for the discovery of *β*‐carboline alkaloids. The skeleton of *β‐*carbolines is a pyrido[3,4‐*b*]indole framework and occurs in both terrestrial and marine organisms [5, 6, 7]. Natural and synthetic *β*‐carboline derivatives have shown protective effects in experimental models of cardiomyocyte injury, including oxidative‐stress and myocardial ischemia/reperfusion models [8, 9, 10]. These observations provide a rationale for evaluating *Cladosporium* derived metabolites in a defined cardiomyocyte‐injury assay.

As part of our continuing investigation of bioactive metabolites from marine microorganisms [11, 12, 13, 14, 15], a chemical investigation of the soft coral‐associated fungus *Cladosporium colombiae* CSGL 10M10 was carried out. This investigation afforded three new alkaloids, colombiains A ‐ C (**1–3**), together with eight known metabolites (**4–11**) (Figure 1). The potential cardioprotective effects of the compounds were evaluated using an OGD/R‐induced injury model in H9c2 cardiomyocytes, and compounds **6** and **11** exhibited protective effects comparable to those of the positive control diazoxide. Further network pharmacology analysis indicated that compound **6** might exert its cardioprotective effects through signaling pathways including diabetic cardiomyopathy, lipid and atherosclerosis, and neurotrophin signaling.

**FIGURE 1:**
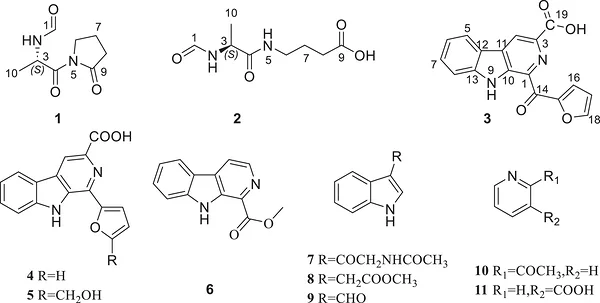
Structures of **1** ‐ **11** from the soft coral‐derived fungus *Cladosporium colombiae* CSGL 10M10.

## 2 Results and Discussion

Compound **1** was obtained as a white amorphous solid and had the molecular formula C_8_H_12_N_2_O_3_ as determined by high‐resolution electrospray ionization mass spectrometry (HRESIMS) ion peak at *m*/*z* 185.0925 [M + H]^+^ (calcd. for 185.0926), implying 4 degrees of unsaturation (Figure S8). The Ultraviolet (UV) absorption maxima was reached at 216 nm (Figure S6). The infrared (IR) spectrum displayed characteristic absorptions for carbonyl (1726 cm^−1^) (Figure S7). The ^1^H nuclear magnetic resonance (NMR), ^13^C NMR and distortionless enhancement by polarization transfer (DEPT) spectrum displayed signals for one methyl at *δ*_H_ 1.52 (3H, d, *J* = 7.0 Hz, H_3_‐10), three methylenes at *δ*_H_ 1.68 (2H, m, H‐7), 2.44 (2H, m, H‐8), and 3.72 (2H, m, H‐6), one methine at *δ*_H_ 6.19 (1H, m, H‐3), and one formyl proton at *δ*_H_ 8.63 (1H, s, H‐1) (Table 1, Figures S9‐S11). The ^13^C NMR, DEPT, and heteronuclear single‐quantum coherence (HSQC) spectra indicated 8 carbon resonances corresponding to one methyl carbon at *δ*_C_ 17.6 (CH_3_, C‐10), three methylene carbons at *δ*_C_ 18.6 (CH_2_, C‐7), 33.8 (CH_2_, C‐8), and 46.2 (CH_2_, C‐6), one aliphatic methine carbon at *δ*_C_ 48.3 (CH, C‐3), one formyl carbon at *δ*_C_ 161.5 (CH, C‐1), and two amide carbonyl carbons at *δ*_C_ 174.5 (C, C‐4) and 175.8 (C, C‐9) (Table 1, Figures S10‐S13).

**TABLE 1:** ^1^H (400 MHz) and ^13^C (100 MHz) NMR data of **1**, **2** and **3**.

	1^a^		2^b^			3^c^	
No.	*δ*_H_, mult (*J*, Hz)	*δ*_C_, type	*δ*_H_, mult (*J*, Hz)	*δ*_C_, type	No.	*δ*_H_, mult (*J*, Hz)	*δ*_C_, type
1	8.63, s	161.5, CH	8.06, s	163.4, CH	1	—	134.3, C
3	6.19, m	48.3, CH	4.50, q (7.3)	48.1, CH	3	—	137.0, C
4	—	174.5, C	—	174.2, C	4	9.16, s	120.6, CH
6	3.72, m	46.2, CH_2_	3.40, t (7.0)	43.6, CH_2_	5	8.47, d (7.9)	122.3, CH
7	1.68, m	18.6, CH_2_	2.13, m	21.6, CH_2_	6	7.37, t (7.5)	121.0, CH
8	2.44, m	33.8, CH_2_	2.29, m	31.2, CH_2_	7	7.65, t (7.7)	129.4, CH
9	—	175.8, C	—	181.6, C	8	7.87, d (8.1)	113.5, CH
10	1.52, d (7.0)	17.6, CH_3_	1.39, d (7.3)	17.7, CH_3_	10	—	136.2, C
					11	—	131.7, C
					12	—	120.5, C
					13	—	142.2, C
					14	—	178.5, C
					15	—	150.7, C
					16	8.20, d (1.6)	148.6, CH
					17	6.90, dd (3.5, 1.6)	113.0, CH
					18	8.79, d (3.5)	125.1, CH
					19	—	166.8, C
					9‐NH	12.37, s	—

^a^ Recorded in Pyridine‐*d_5_*.

^b^ Recorded in CD_3_OD.

^c^ Recorded in DMSO‐*d*_6_.

All protons were assigned to the corresponding carbons from the HSQC spectrum (Table 1, Figure S13). Analysis of the ^1^H‐^1^H COSY spectrum (Figure S12) allowed the compound **1** to be divided into two structural subunits: *N*‐formylalanine and 2‐pyrrolidinone (Table 1, Figure 1). The spin system of *N*‐formylalanine was fully assigned on the basis of combined ^1^H‐^1^H COSY correlations of H‐3 (*δ*_H_ 6.19)/H_3_‐10 (*δ*_H_ 1.52) and ^13^C NMR data (Table 1, Figure S10). The attachment of the *N*‐formyl moiety was established by the HMBC correlations of H‐3 (*δ*_H_ 6.19) to C‐1 (*δ*_C_ 161.5) and C‐4 (*δ*_C_ 174.5), and H_3_‐10 (*δ*_H_ 1.52) to C‐3 (*δ*_C_ 48.3) and C‐4 (*δ*_C_ 174.5). The second spin system was assigned to the 2‐pyrrolidinone subunit based on the continuous ^1^H‐^1^H COSY correlations of H_2_‐6 (*δ*_H_ 3.72)/H_2_‐7 (*δ*_H_ 1.68)/H_2_‐8 (*δ*_H_ 2.44), together with the 2D NMR data (Table 1, Figures S12‐S15). In its HMBC spectrum, the correlations from H_2_‐6 (*δ*_H_ 3.72) to C‐9 (*δ*_C_ 175.8), H_2_‐7 (*δ*_H_ 1.68) to C‐9 (*δ*_C_ 175.8), and H_2_‐8 (*δ*_H_ 2.44) to C‐9 (*δ*_C_ 175.8) supported the assignment of 2‐pyrrolidinone (Figure S14). Finally, the HMBC correlation from H_2_‐6 (*δ*_H_ 3.72) to C‐4 (*δ*_C_ 174.5) established the linkage between C‐4 of *N*‐formylalanine and the amide nitrogen of the 2‐pyrrolidinone moiety in compound **1**.

Biogenetically, the stereocenter at C‐3 of **1** was derived from alanine. As naturally occurring alanine was almost exclusively in the *L*‐configuration [16, 17], the 3*S* configuration was tentatively assigned from a biogenetic perspective. This deduction was further supported by the calculated ECD spectrum, in which the simulated spectrum of the 3*S* isomer showed excellent agreement with the experimental ECD spectrum (Table S4‐S5, Figures 2, S1, S2, and S5). Based on the above evidence, the structure of **1** was determined and named as colombiain A.

**FIGURE 2:**
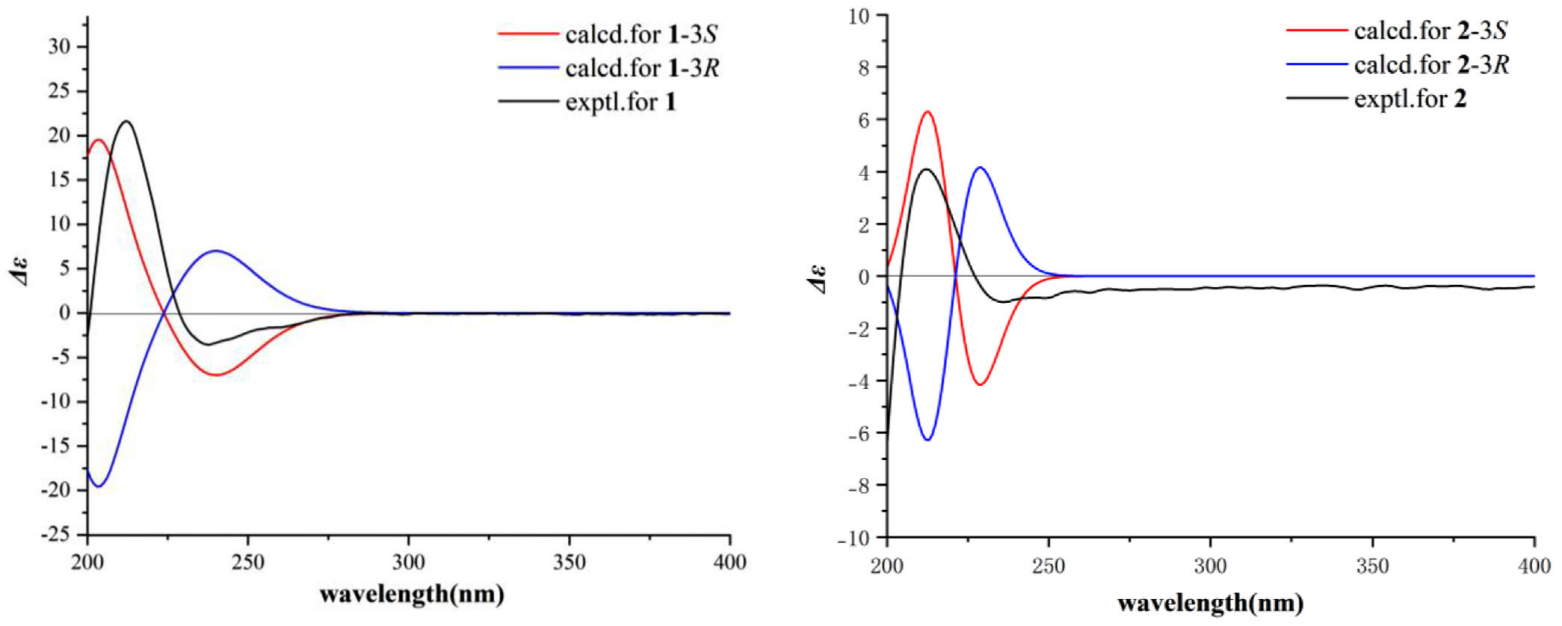
Experimental and calculated ECD spectra of compound **1** and **2**.

Compound **2** was isolated as a white amorphous solid. Its molecular formula was assigned as C_8_H_14_N_2_O_4_ on the basis of the HRESIMS data *m*/*z* 203.1023 [M+H]^+^ (calcd. for 203.1032), indicating 3° of unsaturation (Figure S19). The UV absorption maxima was reached at 200 nm (Figure S17). The IR spectrum revealed the presence of carbonyl (1740 cm^−1^) (Figure S18). A detailed comparison of ^1^H and ^13^C NMR data of compounds **2** and **1** revealed that they were analogues (Table 1, Figures S20‐S26). The key distinction was that the 2‐pyrrolidinone moiety in compound **1** underwent hydrolysis to form a *γ*‐aminobutyric acid unit, which was supported by a mass difference of 18 Da between the two compounds and the disappearance of the HMBC correlations from H_2_‐6 to C‐9. To verify the absolute configuration of **2**, the calculated ECD spectrum was compared with the experimental ECD curve (Table S6‐S7, Figures 2, S3, S4, and S16), which unambiguously assigned the 3*S* configuration to C‐3. Therefore, compound **2** was given the trivial name colombiain B.

Compound **3** was initially obtained as clear light yellow block crystals with a molecular formula of C_17_H_10_N_2_O_4_ based on its HRESIMS peak at *m*/*z* 307.0712 [M+H]^+^ (calcd. for 307.0719), indicating 14° of unsaturation (Figure S29). The UV absorption maxima were reached at 222, 250, 299, and 395 nm (Figure S27). The IR spectrum exhibited characteristic absorption bands at carbonyl (1724, 1697 cm^−1^) and aromatic ring (1620, 1462 cm^−1^) (Figure S28). Comparison of the NMR data (Figures S30‐S36) indicated that compound **3** was highly similar to the known compound Furan‐2‐yl(9H‐pyrido[3,4‐b]indol‐1‐yl) methanone [18], suggesting that **3** was a *β*‐carboline derivative. The most notable difference was the presence of an additional carboxyl carbon signal in **3**, which was consistent with the mass difference of 44 Da observed in their HRESIMS data. The key HMBC correlations from H‐4 (*δ*_H_ 9.16) to C‐19 (*δ*_C_ 166.8) indicated that the carbonyl group was attached to C‐3 (Figure S35). However, no correlation was observed between H‐16 (*δ*_H_ 8.20) with C‐14 (*δ*_C_ 178.5), thereby the planar structure of **3** couldn't be established (Figure 3). Fortunately, **3** was obtained as light yellow block crystals upon standing in MeOH at room temperature, and its structure was established by single‐crystal X‐ray diffraction using Cu K*α* radiation [CCDC 2555402] (Table S1, Figure 4). So, compound **3** was identified as colombiain C.

**FIGURE 3:**
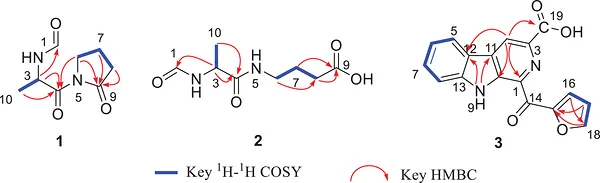
Key ^1^H‐^1^H COSY and HMBC correlations of **1**–**3**.

**FIGURE 4:**
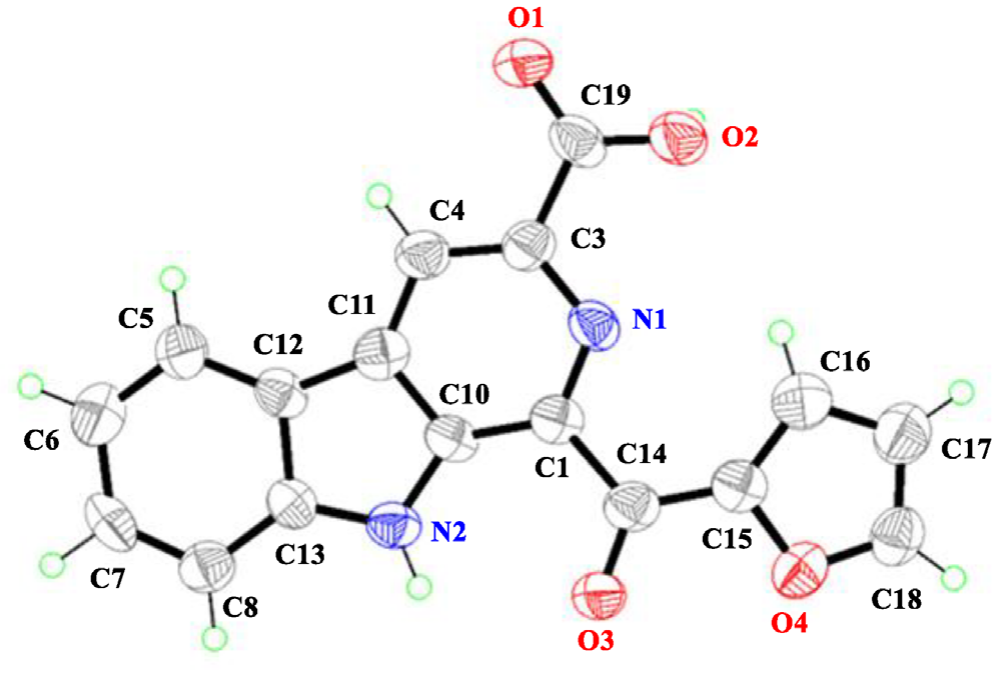
X‐ray crystal structure of **3** (Thermal ellipsoids were drawn at the 50% probability level).

The eight known compounds were identified as 1‐(Furan‐2‐yl)‐9H‐pyrido[3,4‐b]indole‐3‐carboxylic acid (**4**) [19], flazine (**5**) [19], 1‐carbomethoxy‐*β*‐carboline (**6**) [20], *N*‐acetyl‐*β*‐oxotryptamine (**7**) [21], indole‐3‐methylethanoate (**8**) [22], 1H‐indole‐3‐carbaldehyde (**9**) [23], 2‐acetylpyridine (**10**) [24], nicotinic acid (**11**) [25] by comparing their spectroscopic data with the corresponding literature data (Tables S2‐S3, Figures S37‐S44).

Cardiovascular diseases (CVDs) remain the leading cause of mortality worldwide, the pursuit of cardioprotective agents have long been a central focus in cardiovascular drug discovery and development [26]. All isolated compounds were initially evaluated for cytotoxicity against normal H9c2 cardiomyocytes at a concentration of 10 µM, and the results demonstrated that compounds **3** (87.26 ± 4.14)% and **5** (87.95 ± 3.97)% exhibited cytotoxic effects compared to the control group (Figure 5). Through the further assessment of cardioprotective effects using an OGD/R‐induced injury model in H9c2 cells, compounds **6** (71.91 ± 3.44)% and **11** (72.18 ± 2.85)% significantly improved cell viability (Figure 6), showing the same protective effects as the positive control diazoxide (71.52 ± 2.49)%.

**FIGURE 5:**
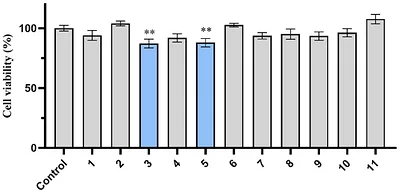
Effects on the viability of H9c2 cardiomyocytes at 10 µM. Data are presented as mean ± SD (n = 3). ^*^ *p* < 0.05, and ^**^ *p* < 0.01 versus the control group.

**FIGURE 6:**
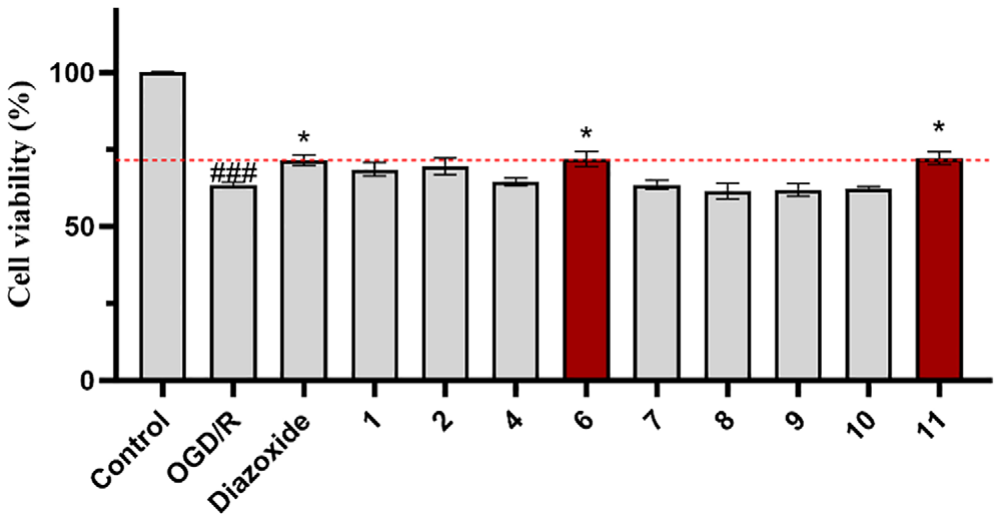
Protective effects against OGD/R‐induced injury in H9c2 cardiomyocytes at 10 µM. Data are presented as mean ± SD (*n* = 3). ^#^ *p* < 0.05, ^##^ *p* < 0.01, and ^###^ *p* < 0.001 versus the control group; ^*^ *p* < 0.05 vs the OGD/R group.

In order to investigate the possible mechanism of the cardioprotective compounds, the potential targets of compound **6** were obtained by Swiss Target Prediction (Figure 7A). In the Venn diagram, all cardioprotection‐related targets overlapped with the targets of **6** (Figure 7A). A protein‐protein interaction (PPI) network was constructed using the STRING database and subsequently visualized and analyzed in Cytoscape 3.10.4 (Figure 7B,C). Topological analysis of the PPI network identified PTGS2, SRC, GSK3B, MMP9, and MAOB as the core targets (Figure 7D). Gene Ontology (GO) functional enrichment (Figure 8A) and Kyoto Encyclopedia of Genes and Genomes (KEGG) pathway enrichment analyses (Figure 8B) indicated that the mechanism of *β*‐carboline alkaloids was primarily associated with neural regulation‐related pathways, while cardioprotection‐related pathways may involve the diabetic cardiomyopathy pathway [27, 28], the lipid and atherosclerosis pathway [29, 30, 31], and the neurotrophin signaling pathway [32, 33]. These network pharmacology results may provide a basis for future investigations into the cardioprotective mechanisms of *β*‐carboline alkaloids.

**FIGURE 7:**
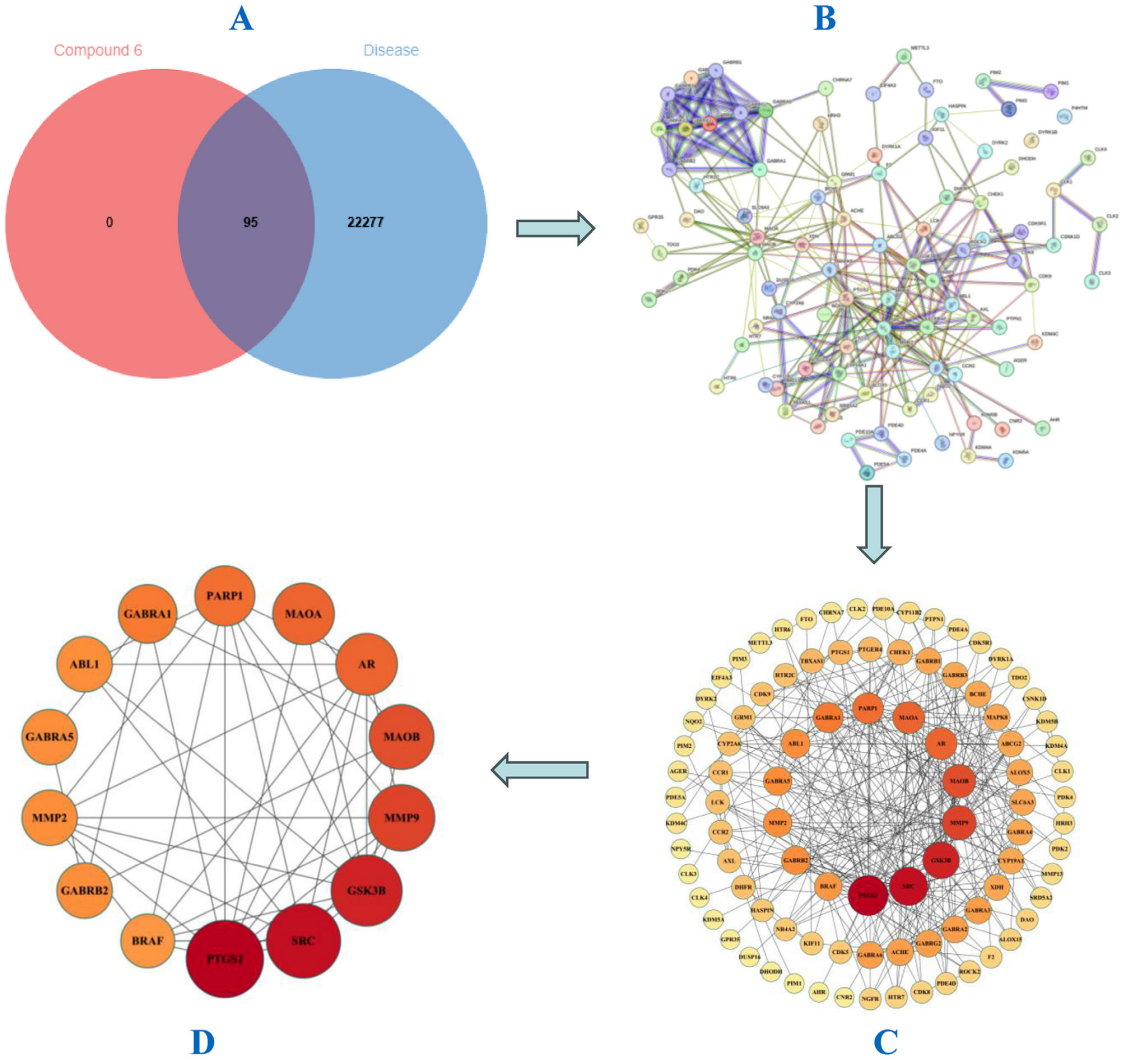
Prediction and screening of potential targets of **6**: (A) Venn diagram showing the intersection between the targets of compound **6** and CVDs targets. (B) PPI network of compound **6** in the context of CVDs treatment. (C) Visualization of the PPI network analysis using Cytoscape software. (D) The top 18 targets selected based on degree value, identified using the cytoNCA plugin.

**FIGURE 8:**
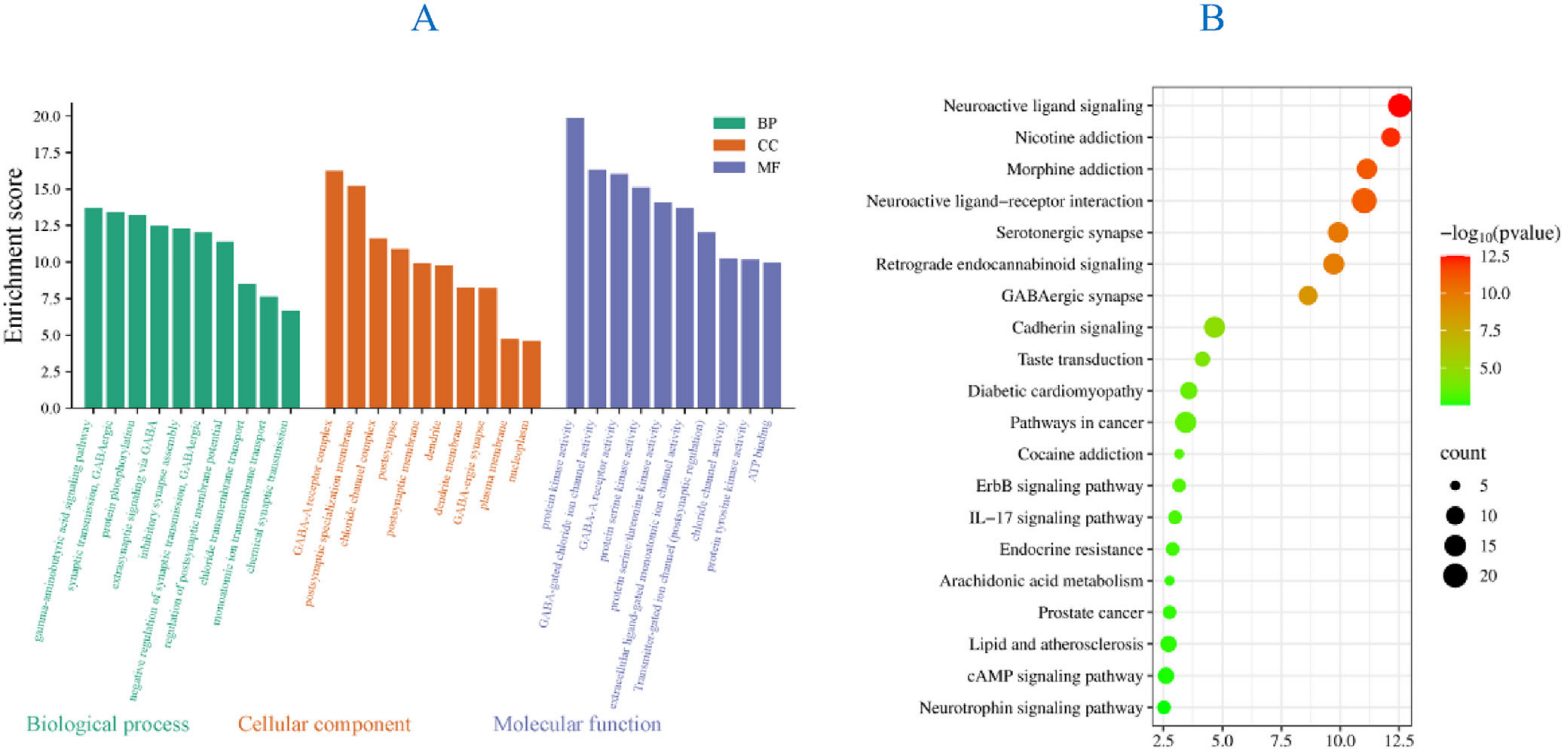
GO and KEGG enrichment analyses of the potential targets of compound **6** for CVDs treatment: (A) GO enrichment analysis and (B) KEGG pathway analysis.

## 3 Conclusions

In conclusion, two new dipeptide derivatives colombiains A (**1**) and B (**2**), one new *β*‐carboline alkaloid colombiain C (**3**), together with three known *β*‐carboline analogs (**4**–**6**), three simple indole alkaloids (**7**–**9**) and two known pyridine derivatives (**10**–**11**), were isolated from the soft coral‐derived fungus *Cladosporium colombiae* CSGL 10M10. Notably, compounds **4** ‐ **11** were isolated from the genus *Cladosporium* for the first time, thereby enriching its chemical structural diversity. Bioactivity assays demonstrated that compounds **6** and **11** exhibited cardioprotective effects comparable to the positive control diazoxide. Furthermore, network pharmacology predictions indicated that *β*‐carboline alkaloids might exert cardioprotective effects through anti‐inflammatory, lipid‐regulating, anti‐atherosclerotic, and myocardial metabolic modulation pathways.

## 4 Experimental Section

### 4.1 General Experimental Procedures

Optical rotations were measured with an MCP 200 digital polarimeter (Anton Paar, Austria). UV and CD spectra were taken using a Circular Dichroism J‐810 spectrometer (JASCO, Japan). IR spectra were recorded on a Shimadzu IRAffinity‐1 Fourier‐transform infrared spectrophotometer (Shimadzu, Japan). X‐ray crystallographic data were acquired using a Rigaku Synergy Custom diffractometer with a HyPix detector (Rigaku, Japan). 1D and 2D NMR spectra were acquired on a Bruker AVANCE III HD 400 MHz NMR spectrometer (Bruker, Germany). Triple TOF 5600+ mass spectrometry system (AB Sciex, U.S.A.). QuikSep semi‐preparative high performance liquid chromatography (HPLC) instrument (Beijing Huideyi Technology Co., Ltd., Beijing, China). Column 1: Kromasil semi‐preparation column (21.2 × 250 mm, 10 µm, Sweden). Column 2: Kromasil column (10 × 250 mm, 5 µm, Sweden). Silica gel (200−300 mesh, Qingdao Marine Chemical Factory, Qingdao, China). ODS gel (S‐50 µm, YMC Co., Ltd., Kyoto, Japan). Methanol, ethyl acetate, petroleum ether and dichloromethane were used for column chromatography (all analytically pure, Tianjin Damao Chemical Reagent Factory, Tianjin, China). Rat cardiomyocyte H9c2 cell line (RRID: CVCL_0286) was obtained from Shanghai Institute for Biological Sciences, Chinese Academy of Science (Shanghai, China).

### 4.2 Fermentation, Extraction

The fungal strain *Cladosporium colombiae* CSGL 10M10 was isolated and purified from a soft coral collected in the Beibu Gulf region of Guangxi, P.R. China. A voucher strain (CSGL 10M10) has been deposited at the Laboratory of Marine Natural Medicine, Guangzhou University of Chinese Medicine, Guangzhou, P.R. China. The fermentation of strain CSGL 10M10 was performed on Zhen‐1 medium (400 × 1 L flasks with NaBr, 100 × 1 L flasks with NaCl) and GPY medium (50 × 1 L flasks, each with NaBr and NaCl). The Zhen‐1 medium contained maltose 4.0%, yeast extract 1.3%, sodium glutamate 1.0%, sorbitol 5.0%, tryptophan 0.05%, MgSO_4_·7H_2_O 0.03%, KH_2_PO_4_ 0.05%, sea salt 3.5%. The GPY medium consisted of glucose 1.0%, yeast extract 0.2%, peptone 1.0%, sea salt 0.25%. After fermentation, the mycelia and broth were filtered and separated by gauze. The broth was concentrated at 50°C and sequentially extracted three times with EtOAc (ethyl acetate) and *n*‐BuOH (*n*‐ butanol, *v*:*v* = 1:1) in sequence. The respective organic phases were concentrated under reduced pressure to afford the EtOAc and *n*‐BuOH extracts of the broth. The mycelia were extracted with MeOH three times (each for 72 h). The combined MeOH extracts were dissolved in distilled water and then sequentially partitioned with PE (petroleum ether), EtOAc, and *n*‐BuOH (*v*:*v* = 1:1), each three times. Based on the TLC and ^1^H NMR, the EtOAc and *n*‐BuOH fractions obtained from the broth and mycelia of strain 10M10 across all tested media were combined to afford the total EtOAc extract (183.1 g) and total *n*‐BuOH extract (417.0 g), respectively.

### 4.3 Isolation and Purification

The total EtOAc extract (183.1 g) was subjected to silica gel column chromatography and elutied with a gradient of PE—EtOAc (100:0 ‐ 0:100, *v*/*v*) to yield seven fractions (Fr.e1 to Fr.e7). Similarly, the total *n*‐BuOH extract (417.0 g) was separated by silica gel column chromatography with CH_2_Cl_2_ ‐ CH_3_OH (100:0 ‐ 0:100, *v*/*v*) to afford seven fractions (Fr.n1 to Fr.n7). The TLC analysis revealed partial overlap between the polar fractions of the EtOAc extract and the nonpolar fractions of the *n*‐BuOH extract. Based on TLC and ^1^H NMR analysis, these fractions were further combined to afford eight primary fractions (Fr.1 ‐ Fr.8).

Fr.2 (24.1 g) was subjected to ODS open column chromatography eluting with a gradient of CH_3_OH‐H_2_O (30:70 ‐ 100:0, *v*/*v*) to yield 9 sub‐fractions (Fr.2‐1 to Fr.2‐9). Fr.2‐3 (2.4 g) was separated by semi‐preparative HPLC with CH_3_OH‐H_2_O (Column 2, 51:49, *v*/*v*, 2.0 mL/min) to obtain 10 sub‐fractions (Fr.2‐3‐1 to Fr.2‐3‐10). Fr.2‐3‐5 (162.4 mg) was purified by semi‐preparative HPLC (Column 2, CH_3_OH‐H_2_O, 35:65, *v*/*v*, 2.0 mL/min) to afford compound **6** (1.4 mg, *t*_R_ = 23.5 min) and **8** (18.9 mg, *t*_R_ = 52 min). Fr.2‐4 (0.4 g) was purified by semi‐preparative HPLC (Column 2, CH_3_OH‐H_2_O, 55:45, *v*/*v*, 2.0 mL/min) to yield nine sub‐fractions (Fr.2‐4‐1 to Fr.2‐4‐9). Fr.2‐4‐5 (162.4 mg) was further purified by semi‐preparative HPLC (Column 2, CH_3_CN‐H_2_O, 35:65, *v*/*v*, 2.0 mL/min) to yield compound **7** (6.8 mg, *t*_R_ = 12.8 min) and **4** (2.7 mg, *t*_R_ = 17.0 min).

Fr.3 (61.4 g) was fractionated by ODS open column chromatography eluting with a gradient of CH_3_OH‐H_2_O (30:70‐100:0, *v*/*v*) to yield seven sub‐fractions (Fr.3‐1 to Fr.3‐7). Fr.3‐2 (2.3 g) was purified by semi‐preparative HPLC (Column 1, CH_3_CN‐H_2_O, 15:85, *v*/*v*, 5.0 mL/min) to give 11 sub‐fractions (Fr.3‐2‐1 to Fr.3‐2‐11). Fr.3‐2‐2 was further purified by semi‐preparative HPLC (Column 2, CH_3_OH‐H_2_O, 6:94, *v*/*v*, 2.0 mL/min) to afford **11** (70 mg, *t*_R_ = 23.5 min). Compound **2** (3.4 mg, *t*_R_ = 16.0 min) and **1** (6.6 mg, *t*_R_ = 20.0 min) were obtained from Fr.3‐2‐8 by semi‐preparative HPLC (Column 2, CH_3_OH‐H_2_O, 15:85, *v*/*v*, 2.0 mL/min). Furthermore, Fr.3‐2‐10 afforded compound **10** (3.2 mg, *t*_R_ = 43.0 min) and **9** (3.9 mg, *t*_R_ = 54.0 min), which were purified by semi‐preparative HPLC (Column 2, CH_3_OH‐H_2_O, 20:80, *v*/*v*, 2.0 mL/min). Fr.3‐4 (6.3 g) was separated by semi‐preparative HPLC (Column 1, CH_3_OH‐H_2_O, 45:55, *v*/*v*, 5.0 mL/min) to yield 15 sub‐fractions (Fr.3‐4‐1 to Fr.3‐4‐15). Fr.3‐4‐7 (0.1 g) was purified by semi‐preparative HPLC (Column 2, CH_3_OH‐H_2_O, 38:62, *v*/*v*, 2.0 mL/min) to afford **3** (3.3 mg, *t*_R_ = 15.0 min). Fr.3‐5 (8.2 g) was separated by semi‐preparative HPLC (Column 1, CH_3_OH‐H_2_O, 60:40, *v*/*v*, 5.0 mL/min) to yield 16 sub‐fractions (Fr.3‐5‐1 to Fr.3‐5‐16). Fr.3‐5‐13 (0.9 g) was further purified by semi‐preparative HPLC (Column 2, CH_3_CN‐H_2_O, 55:45, *v*/*v*, 2.0 mL/min) to give 16 sub‐fractions (Fr.3‐5‐13‐1 to Fr.3‐5‐13‐16), and then compound **5** (5.7 mg, *t*_R_ = 10.5 min) was obtained from Fr.3‐5‐13‐13 (27.1 mg) by semi‐preparative HPLC (Column 2, CH_3_OH‐H_2_O, 51:49, *v*/*v*, 2.0 mL/min).

### 4.4 ECD Calculation

The quantum chemical ECD calculations of **1** and **2** were conducted following modified protocols as reported previously [11, 12].

### 4.5 Cardiomyocyte Protective Activity Assay

The activity assays were performed in accordance with previously reported methods [34, 35].

### 4.6 Network Pharmacology and Go/Kegg Pathway Enrichment Analysis

The experimental procedures of the network pharmacology and GO/KEGG pathway enrichment analysis were described in the Supporting Information.

### 4.7 Structural Characterizations of 1–3

*Colombiain A (* ***1***): white amorphous solid; [*α*]25 *D*‐59.60 (*c* 0.10, MeOH); HRESIMS *m/z*: 185.0925 [M+H]^+^ (calcd. for: 185.0926); UV(MeOH) *λ*_max_ 216 (2.42) nm; IR*ν*_max_: 3354, 2949, 2924, 2833, 1726, 1662, 1020 cm^−1^; ^1^H NMR and ^13^C NMR data see Table 1.

*Colombiain B (* ***2***): white amorphous solid; [*α*]25 *D*‐32.92 (*c* 0.12, MeOH); HRESIMS *m/z*: 203.1023 [M+H]^+^ (calcd. for: 203.1032); UV (MeOH) *λ*_max_ 200 (2.90) nm; IR*ν*_max_: 3269, 2924, 1740, 1681, 1531 cm^−1^; ^1^H NMR and ^13^C NMR data see Table 1.

*Colombiain C (* ***3***): light yellow block crystals; HRESIMS *m/z*: 307.0712 [M+H]^+^ (calcd. for: 307.0719); UV (MeOH) *λ*_max_ 222 (4.46) nm, 250 (2.35) nm, 299 (4.27) nm, 395 (2.31) nm; IR*ν*_max_: 3260, 2924, 2856, 1724, 1697, 1620, 1462 cm^−1^; ^1^H NMR and ^13^C NMR data see Table 1.

### 4.8 X‐ray Crystal Structure Analysis

The crystal of **3** was obtained by slow evaporation from MeOH at room temperature. Crystallographic data for **3** have been uploaded to the Cambridge Crystallographic Data Centre (CCDC 2555402).

*Crystal data for* ***3***: C_17_H_10_N_2_O_4_ (*M* = 306.27 g/mol): monoclinic, space group I2/a (no. 15), *a* = 23.900(4) Å, *b* = 4.5384(6) Å, *c* = 31.516(3) Å, *β* = 103.967(14), *V* = 3317.4(8) Å^3^, *Z* = 8, *T* = 120.00(10) K, *μ*(Cu Kα) = 0.747 mm^−1^, *Dcalc* = 1.226 g/cm^3^, 8499 reflections measured (5.78° ≤ 2θ ≤ 133.196°), 2838 unique (*R*_int_ = 0.1052, *R*_sigma_ = 0.1061) which were used in all calculations. The final *R*_1_ was 0.1367 (I > 2σ(I)) and *wR*_2_ was 0.3448 (all data).

## Author Contributions

**Jing Xie** and **Bing‐Bing Liu** prepared the manuscript. **Jing Xie** carried out the isolation and structure elucidation and wrote the article. Bing‐Bing Liu, **Yang‐Ying Zheng**, **Jun‐Feng Huan** and **Hui‐Mei Xie** participated in column chromatography of fungal extract. **Xiu‐Ping Lin** was responsible for isolation, purification and identification of the fungi. **Yuan‐Yuan Cheng** and **Xiao‐Yu Tian** supervised the smooth progress of the pharmacological experiments. **Cui‐Xian Zhang** designed the experiments, supervised the research, and revised the article.

## Funding

This work was financially supported by the Special Project for Marine Economic Development of Department of Natural Resources of Guangdong Province (GDNRC[2024]25).

## Conflicts of Interest

The authors declare no conflicts of interest.

## Supporting information




**Supporting file 1**: cbdv71769‐sup‐0001‐SuppMat.pdf

## Data Availability

The data that supports the findings of this study are available in the Supporting Information of this article.
