# Supplementary material for: Colombiains A–C, Three New Alkaloids From the Soft Coral‐Derived Fungus Cladosporium colombiae CSGL 10M10
Source: Chem Biodivers. 2026 Sep 30;23(10):e71769. doi: 10.1002/cbdv.71769 (PMC13626256; doi:10.1002/cbdv.71769)
Supplement: Supplementary file 1 [file CBDV-23-e71769-s001.pdf]

Supporting Information

Colombiains A - C, Three New Alkaloids from the Soft Coral-derived Fungus *Cladosporium colombiae* CSGL 10M10

Jing Xie[#, 1] | Bing-Bing Liu[#, 1] | Yang-Ying Zheng[1] | Jun-Feng Huan[1] | Xiao-Yu Tian[1] | Xiu-Ping Lin[2] | Hui-Mei Xie[1] | Yuan-Yuan Cheng*[, 1] | Cui-Xian Zhang*[, 1]

1. School of Pharmaceutical Sciences, Guangzhou University of Chinese Medicine, Guangzhou, Guangdong 510006, People's Republic of China.

2. State Key Laboratory of Tropical Oceanography/Guangdong Key Laboratory of Marine Materia Medica, South China Sea Institute of Oceanology, Chinese Academy of Sciences, Guangzhou, 510301, People's Republic of China.

[#] Jing Xie and Bing-Bing Liu[#] contributed equally to this work.

[*] **Correspondence**: Cui-Xian Zhang (zhangcuixian@gzucm.edu.cn) | Yuan-Yuan Cheng (chengyuanyuan@gzucm.edu.cn)

Contents

1. Crystal data of 3 4

2. Structural characterizations of 4 - 11 5

3. Network Pharmacology and GO/KEGG Pathway Enrichment Analysis 6

Table S1 Unit cell parameters of **3** 4

Table S2 ^{1}H (400MHz) and ^{13}C (100MHz) NMR Data of **4 - 6** 6

Table S3 ^{1}H (400MHz) and ^{13}C (100MHz) NMR Data of **7 - 9** in CD_3OD 6

Table S4 The energies and boltzmann distributions for stable conformers of **1**-3*S* 7

Table S5 Cartesian coordinates of all conformers of compound **1** 8

Table S6 The energies and boltzmann distributions for stable conformers of **2**-3*S* 13

Table S7 Cartesian coordinates of all conformers of compound **2** 13

Figure S1 **1**-3*S* and **1**-3*R* 7

Figure S2 Stable conformers of **1**-3*S* at the B3LYP/6-31+G(d) level in methanol 7

Figure S3 **2**-3*S* and **2**-3*R* 13

Figure S4 Stable conformers of **2**-3*S* at the B3LYP/6-31+G(d) level in methanol 13

Figure S5 ECD spectrum of **1** 19

Figure S6 UV spectrum of **1** 19

Figure S7 IR spectrum of **1** 20

Figure S8 HRESIMS spectrum of **1** 20

Figure S9 ^{1}H NMR (400MHz) spectrum of **1** in Pyridine-d_5 20

Figure S10 ^{13}C NMR (100MHz) spectrum of **1** in Pyridine-d_5 21

Figure S11 DEPT 135 (100MHz) spectrum of compound **1** in Pyridine-d_5 21

Figure S12 ^{1}H-^{1}H COSY spectrum of **1** in Pyridine-d_5 22

Figure S13 HSQC spectrum of **1** in Pyridine-d_5 22

Figure S14 HMBC spectrum of **1** in Pyridine-d_5 23

Figure S15 NOESY spectrum of **1** in Pyridine-d_5 23

Figure S16 ECD spectrum of **2** 24

Figure S17 UV spectrum of **2** 24

Figure S18 IR spectrum of **2** 25

Figure S19 HRESIMS spectrum of **2** 25

Figure S20 ^{1}H NMR (400MHz) spectrum of **2** in CD_3OD 25

Figure S21 ^{13}C NMR (100MHz) spectrum of **2** in CD_3OD 26

Figure S22 DEPT 135 (100MHz) spectrum of compound **2** in CD_3OD 26

Figure S23 ^{1}H-^{1}H COSY spectrum of **2** in CD_3OD 27

Figure S24 HSQC spectrum of **2** in CD_3OD 27

Figure S25 HMBC spectrum of **2** in CD_3OD 28

Figure S26 NOESY spectrum of **2** 28

Figure S27 UV spectrum of **3** 29

Figure S28 IR spectrum of **3** 29

Figure S29 HRESIMS spectrum of **3** 29

Figure S30 ^{1}H NMR (400MHz) spectrum of **3** in DMSO-d_6 30

Figure S31 ^{13}C NMR (100MHz) spectrum of **3** in DMSO-d_6 30

Figure S32 DEPT 135 (100MHz) spectrum of **3** in DMSO-d_6 31

Figure S33 ^{1}H-^{1}H COSY spectrum of **3** in DMSO-d_6 31

Figure S34 HSQC spectrum of **3** in DMSO-d_6 32

Figure S35 HMBC spectrum of **3** in DMSO-d_6 32

Figure S36 NOESY spectrum of **3** in DMSO-d_6 33

Figure S37 ^{1}H NMR (400MHz) spectrum of **4** in DMSO-d_6 33

Figure S38 ^{1}H NMR (400MHz) spectrum of **5** in DMSO-d_6 34

Figure S39 ^{1}H NMR (400MHz) spectrum of **6** in CD_3OD 34

Figure S40 ^{1}H NMR (400MHz) spectrum of **7** in CD_3OD 35

Figure S41 ^{1}H NMR (400MHz) spectrum of **8** in CD_3OD 35

Figure S42 ^{1}H NMR (400MHz) spectrum of **9** in CD_3OD 36

Figure S43 ^{1}H NMR (400MHz) spectrum of **10** in CD_3OD 36

Figure S44 ^{1}H NMR (400MHz) spectrum of **11** in CD_3OD 37

References 37

1. Crystal data of 3

Crystal parameters of **3** are listed in Table S1. Crystallographic data for **3** have been deposited in the Cambridge Crystallographic Data Center as supplementary publication no. CCDC 2555402 Copies of the data can be obtained, free of charge, on application to the Director, CCDC, 12 Union Road, Cambridge CB2 1EZ, UK (fax: + 44 - (0)1223-336033, or e-mail: deposit@ccdc.cam.ac. uk).

Table S1 Unit cell parameters of **3**

Identification code	cc6_50
Empirical formula	$C_{17}H_{10}N_2O_4$
Formula weight	306.27
Temperature/K	120.00(10)
Crystal system	monoclinic
Space group	I2/a
a/Å	23.900(4)
b/Å	4.5384(6)
c/Å	31.516(3)
α/°	90
β/°	103.967(14)
γ/°	90
Volume/Å^3	3317.4(8)
Z	8
ρ_{calc}g/cm^3	1.226
μ/mm^{-1}	0.747
F(000)	1264.0
Crystal size/mm^3	0.14 × 0.13 × 0.12
Radiation	Cu Kα (λ = 1.54184)
2θ range for data collection/°	5.78 to 133.196
Index ranges	$-26 \le h \le 28$, $-5 \le k \le 3$, $-37 \le l \le 37$
Reflections collected	8499
Independent reflections	2838 [R_{int} = 0.1052, R_{sigma} = 0.1061]
Data/restraints/parameters	2838/0/208
Goodness-of-fit on F^2	1.164
Final R indexes [I>=2σ (I)]	R_1 = 0.1367, wR_2 = 0.3250
Final R indexes [all data]	R_1 = 0.1658, wR_2 = 0.3448
Largest diff. peak/hole / e Å^{-3}	0.53/-0.47

2. Structural characterizations of 4 - 11

1-(Furan-2-yl)-9H-pyrido[3,4-b]indole-3-carboxylic acid (**4**): light yellow solid; HRESIMS *m/z*: 277.0616 $[M-H]^-$ (calcd. for 277.0613); ^1H and ^{13}C NMR data see Table S2.

flazine (**5**): light yellow solid; HRESIMS *m/z*: 307.0785 $[M-H]^-$ (calcd. for 307.0719); ^1H and ^{13}C NMR data see Table S2.

1-carbomethoxy-*β*-carboline (**6**): white solid; HRESIMS *m/z*: 227.0804 $[M+H]^+$ (calcd. for 227.0821), 249.0627 $[M+Na]^+$ (calcd. for 249.0640); ^1H and ^{13}C NMR data see Table S2.

N-acetyl-*β*-oxotryptamine (**7**): white solid; HRESIMS *m/z*: 217.0965 $[M+H]^+$ (calcd. for 217.0977), 239.0790 $[M+Na]^+$ (calcd. for 239.0796); ^1H and ^{13}C NMR data see Table S3.

indole-3-methylethanoate (**8**): light yellow solid; HRESIMS *m/z*: 190.0860 $[M+H]^+$ (calcd. for 190.0868); ^1H and ^{13}C NMR data see Table S3.

1*H*-indole-3-carbaldehyde (**9**): white solid; HRESIMS *m/z*: 146.0625 $[M+H]^+$ (calcd. for 146.0606); ^1H and ^{13}C NMR data see Table S3.

2-acetylpyridine (**10**): white solid; HRESIMS *m/z*: 122.0601 $[M+H]^+$ (calcd. for 122.0606); ^1H NMR (400 MHz, CD_3OD) δ_H 8.52 (d, 8.3, H-2), 8.07 (d, 7.7, H-3), 7.51 (t, 7.7, H-5), 7.12, (t, 7.4, H-4), 2.19 (s, H_3-7); ^{13}C NMR (100 MHz, CD_3OD) δ_C 171.4 (C, C-6), 142.2 (C, C-1), 134.6 (CH, C-5), 132.5 (CH, C-3), 123.9 (CH, C-4), 121.2 (CH, C-2), 25.0 (CH_3, C-7).

nicotinic acid (**11**): light yellow solid; HRESIMS *m/z*: 122.0230 $[M-H]^-$ (calcd. for 122.0242); ^1H NMR (400 MHz, CD_3OD) δ_H 9.07, (s, H-5),8.79 (d, 4.8, H-4), 8.27 (d, 8.0, H-2), 7.54 (dd, 7.9, 4.9, H-3); ^{13}C NMR (100 MHz, CD_3OD) δ_C 166.3 (C, C-6), 153.3 (CH, C-5), 150.2 (CH, C-4), 137.0 (CH, C-2), 126.6 (C, C-1), 123.9 (CH, C-3).

Table S2 ^{1}H (400MHz) and ^{13}C (100MHz) NMR Data of **4 - 6**

No.	4[a]		5[a]		6[b]	
	**δ_H, mult (*J* in Hz)**	**δ_C, type**	**δ_H, mult (*J* in Hz)**	**δ_C, type**	**δ_H, mult (*J* in Hz)**	**[b2]δ_C, type**
1	-	137.8, C	-	131.6, C		143.3, C
3	-	132.4, C	-	137.8, C	8.44, d, 5.0	138.8, CH
4	8.84, s	115.9, CH	8.81, s	115.5, CH	8.34, d, 5.0	120.0, CH
5	8.41, d, 7.9	122.0, CH	8.38, d, 7.8	122.0, CH	8.24, d, 7.9	122.8, CH
6	7.33, t, 7.5	120.6, CH	7.33, t, 7.4	120.5, CH	7.33, t, 7.5	130.5, CH
7	7.63, t, 7.6	128.9, CH	7.63, t, 7.6	128.8, CH	7.62, t, 7.7	121.8, CH
8	7.81, d, 8.2	113.0, CH	7.82, d, 8.2	112.8, CH	7.73, d, 8.3	113.5, CH
10	-	132.1, C	-	132.1, C	-	133.6, C
11	-	129.9, C	-	129.9, C	-	121.7, C
12	-	120.9, C	-	121.0, C	-	130.7, C
13	-	141.7, C	-	141.5, C	-	137.8, C
14	-	152.4, C	-	151.1, C	-	167.3, C
15	7.49, d, 3.4	110.2, CH	7.44, d, 3.2	111.0, CH	4.12, s	53.0, CH_3
16	6.83, dd, 3.3, 3.4	112.5, CH	6.62, d, 3.2	109.2, CH	-	-
17	8.02, d, 3.3	144.3, CH	-	157.2, C	-	-
18	-	166.6, C	4.68, s	56.0, CH_2	-	-
19	-	-	-	166.7, C	-	-
9-NH	11.88, s	-	11.61, s		-	-

[a]Recorded in DMSO-d_6. [b]Recorded in CD_3OD.

Table S3 ^{1}H (400MHz) and ^{13}C (100MHz) NMR Data of **7 - 9** in CD_3OD

No.	7		8		9	
	**δ_H, mult (*J* in Hz)**	**δ_C, type**	**δ_H, mult (*J* in Hz)**	**δ_C, type**	**δ_H, mult (*J* in Hz)**	**δ_C, type**
2	8.21, m, overlapped	134.3, CH	7.15, s	124.7, CH	8.1, s	139.7, CH
3	-	115.8, C	-	108.5, C	-	120.1, C
4	-	126.9, C	-	128.6, C	-	125.7, C
5	8.21, m, overlapped	122.7, CH	7.51, d, 7.9	119.3, CH	8.16, d, 7.1	125.0, CH
6	7.22, m, overlapped	123.3, CH	7.01, t, 8.0	119.9, CH	7.24, t, 7.0	122.4, CH
7	7.22, m, overlapped	124.4, CH	7.1, t, 8.2	122.5, CH	7.28, t, 7.2	123.6, CH
8	7.45, d, 7.1	113.0, CH	7.34, d, 8.1	112.3, CH	7.48, d, 7.8	113.1, CH
9	-	138.3, C	-	138.0, C	-	138.9, C
10	-	191.9, C	3.77, s	31.9, CH_2	9.88, s	187.4, C
11	4.59, s	47.1, CH_2	-	174.8, C	-	-
12	-	173.7, C	3.68, s	52.3, CH_3	-	-
13	2.07, s	22.5, CH_3	-	-	-	-

3. Network Pharmacology and GO/KEGG Pathway Enrichment Analysis

The prediction of potential targets of compound **6** and disease targets for network pharmacology was performed in accordance with previously reported methods [1, 2]. GO functional enrichment analysis yielded 241 biological process (BP) terms, with the most significantly enriched terms including the gamma-aminobutyric acid signaling pathway, synaptic transmission (GABAergic), and protein phosphorylation, among others. A total of 240 cellular component (CC) terms were obtained, primarily

involving the GABA-A receptor complex, postsynaptic specialization membrane, and chloride channel complex. Additionally, 238 molecular function (MF) terms were identified, mainly associated with protein kinase activity, GABA-gated chloride ion channel activity, and GABA-A receptor activity. The top 10 enriched terms in each of the three GO categories (BP, CC, and MF) ranked by enrichment significance are presented in Figure 8A. KEGG pathway enrichment analysis identified a total of 50 enriched pathways, with the core pathways encompassing neuroactive ligand signaling, nicotine addiction, morphine addiction, and neuroactive ligand–receptor interaction. The top 20 KEGG pathways ranked by *P*-value (from the most to the least significant) are shown in Figure 8B.

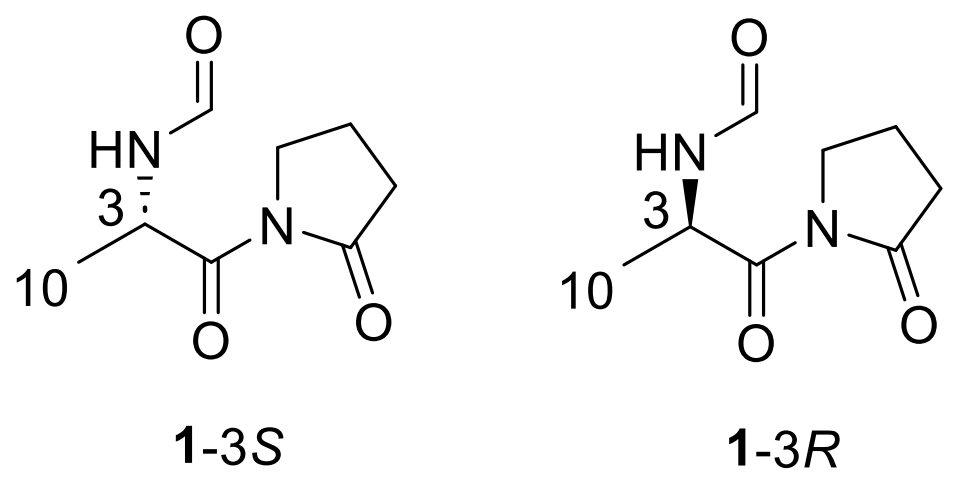

Figure S1 **1**-3*S* and **1**-3*R*

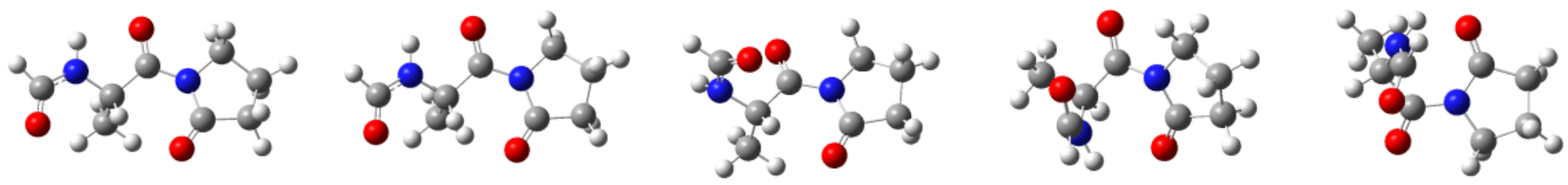

Figure S2 Stable conformers of **1**-3*S* at the B3LYP/6-31+G(d) level in methanol

Table S4 The energies and boltzmann distributions for stable conformers of **1**-3*S*

Conformations	Energy (a.u)	Δ G(kcal/mol)	%
1	-647.170941	0	42.97%
2	-647.170729	0.13	34.33%
3	-647.169704	0.78	11.59%
4	-647.169058	1.18	5.85%
5	-647.168956	1.25	5.25%

Table S5 Cartesian coordinates of all conformers of compound **1**

Conformer **1**-c1				
Number	Element	Coordinates (Angstroms)		
		X	Y	Z
1	N	2.20806	-1.038451	-1.471298
2	C	1.422819	-0.126692	-0.657511
3	C	0.010638	-0.694273	-0.605602
4	N	-0.88567	-0.194232	0.335772
5	C	-2.277804	-0.677569	0.31075
6	C	3.562948	-1.039173	-1.446114
7	C	-0.616681	0.600097	1.46646
8	C	-1.912788	0.69354	2.261334
9	C	-3.002978	0.284659	1.264083
10	O	4.250516	-0.284503	-0.775099
11	O	-0.373608	-1.540753	-1.4013
12	O	0.451695	1.095891	1.754547
13	C	1.410266	1.298773	-1.244637
14	H	1.692143	-1.694732	-2.044194
15	H	1.850413	-0.077645	0.345993
16	H	-2.311438	-1.716117	0.659754
17	H	-2.655115	-0.658471	-0.712329
18	H	3.993311	-1.812387	-2.112844
19	H	-1.838341	-0.013341	3.098334
20	H	-2.022687	1.69374	2.685576
21	H	-3.35118	1.162436	0.709609
22	H	-3.871088	-0.182556	1.734414
23	H	0.859183	1.982992	-0.596063
24	H	2.444295	1.643046	-1.315522
25	H	0.963091	1.295698	-2.244113

Conformer **1**-c2				
Number	Element	Coordinates (Angstroms)		
		X	Y	Z
1	N	1.862401	0.493077	-2.065352
2	C	1.239518	0.589333	-0.75601
3	C	-0.150906	-0.01939	-0.891168
4	N	-0.828602	-0.433593	0.252524
5	C	-2.199885	-0.957744	0.116492
6	C	3.203566	0.595091	-2.231754
7	C	-0.382459	-0.440435	1.58728
8	C	-1.523652	-0.988314	2.4344
9	C	-2.426878	-1.717514	1.433335
10	O	4.008165	0.777704	-1.330873
11	O	-0.700147	-0.119447	-1.980097
12	O	0.699421	-0.061362	1.982564
13	C	1.136802	2.052471	-0.280189
14	H	1.243266	0.356212	-2.854501
15	H	1.838368	0.033637	-0.033175
16	H	-2.265616	-1.583417	-0.773716
17	H	-2.897549	-0.121361	-0.008378
18	H	3.502307	0.491254	-3.293638
19	H	-1.128042	-1.608992	3.240951
20	H	-2.03306	-0.13208	2.89632
21	H	-3.479972	-1.72776	1.723197
22	H	-2.098198	-2.756014	1.321268
23	H	0.710843	2.109193	0.723733
24	H	2.145737	2.470163	-0.252913
25	H	0.524681	2.635788	-0.975848

Conformer **1**-c3				
Number	Element	Coordinates (Angstroms)		
		X	Y	Z
1	N	2.114477	-0.008722	-1.976002
2	C	1.502184	0.411456	-0.725965
3	C	-0.023833	0.28029	-0.84901
4	N	-0.772641	0.007223	0.296952
5	C	-2.221651	-0.222131	0.165237
6	C	2.093811	-1.332317	-2.315681
7	C	-0.339286	-0.063969	1.628297
8	C	-1.556004	-0.411277	2.477483
9	C	-2.584987	-0.94337	1.473173
10	O	1.761211	-2.231691	-1.563211
11	O	-0.591343	0.412562	-1.919687
12	O	0.787349	0.150541	2.029349
13	C	1.882081	1.858994	-0.403018
14	H	2.099069	0.65525	-2.740298
15	H	1.862494	-0.247531	0.061157
16	H	-2.416719	-0.806177	-0.734437
17	H	-2.738587	0.739275	0.061155
18	H	2.448594	-1.510522	-3.348879
19	H	-1.273864	-1.111026	3.26722
20	H	-1.895832	0.513109	2.963181
21	H	-3.620092	-0.758684	1.769904
22	H	-2.457388	-2.023089	1.344414
23	H	1.484839	2.140948	0.574156
24	H	2.971198	1.956356	-0.374338
25	H	1.48494	2.544479	-1.161132

Conformer **1**-c4				
Number	Element	Coordinates (Angstroms)		
		X	Y	Z
1	N	2.084102	0.098995	-0.374536
2	C	1.284719	1.323408	-0.35524
3	C	-0.212808	1.027289	-0.513276
4	N	-0.763208	0.007482	0.287767
5	C	-2.126549	-0.458222	-0.017828
6	C	1.9253	-0.837154	-1.348466
7	C	-0.289902	-0.49187	1.499812
8	C	-1.356687	-1.432827	2.046598
9	C	-2.25977	-1.724407	0.842159
10	O	1.218216	-0.706908	-2.335495
11	O	-0.934886	1.629123	-1.280996
12	O	0.763534	-0.204459	2.044076
13	C	1.744516	2.326842	-1.411493
14	H	2.434425	-0.215199	0.520814
15	H	1.417435	1.740857	0.647869
16	H	-2.215619	-0.636546	-1.090113
17	H	-2.853011	0.314567	0.258458
18	H	2.527437	-1.747013	-1.157653
19	H	-0.889646	-2.309816	2.500629
20	H	-1.893098	-0.898463	2.841881
21	H	-3.297603	-1.929427	1.114702
22	H	-1.878008	-2.587874	0.287512
23	H	1.157416	3.24583	-1.344833
24	H	2.801039	2.56069	-1.25298
25	H	1.612636	1.905104	-2.409367

Conformer **1**-c5				
Number	Element	Coordinates (Angstroms)		
		X	Y	Z
1	N	2.185846	-0.430492	-0.038422
2	C	1.716357	0.836319	-0.570332
3	C	0.330351	1.264158	-0.069628
4	N	-0.680478	0.30703	0.156653
5	C	-1.883255	0.750684	0.88876
6	C	2.41707	-0.563124	1.298261
7	C	-0.846866	-0.961254	-0.398391
8	C	-2.204622	-1.481667	0.0561
9	C	-2.593241	-0.568162	1.223324
10	O	2.389142	0.353868	2.102916
11	O	0.087144	2.432566	0.160578
12	O	-0.077387	-1.542294	-1.146109
13	C	1.780896	0.85576	-2.101158
14	H	1.92014	-1.258341	-0.558346
15	H	2.365227	1.61826	-0.169681
16	H	-2.487452	1.400378	0.245409
17	H	-1.58297	1.330309	1.762126
18	H	2.660348	-1.605768	1.581355
19	H	-2.895483	-1.381197	-0.791484
20	H	-2.138087	-2.544967	0.297684
21	H	-2.201034	-0.970742	2.163011
22	H	-3.672077	-0.437612	1.335231
23	H	1.461664	1.834259	-2.471246
24	H	2.810706	0.678538	-2.425724
25	H	1.138071	0.083509	-2.530906

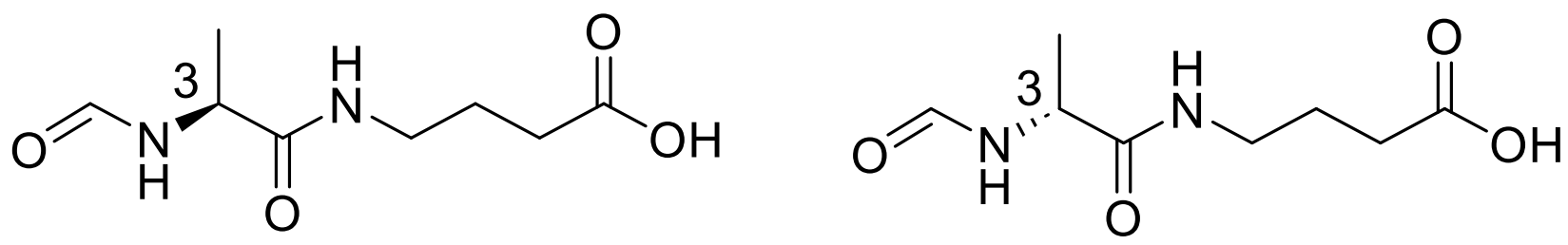

2-3*S* **2**-3*R*

Figure S3 **2**-3*S* and **2**-3*R*

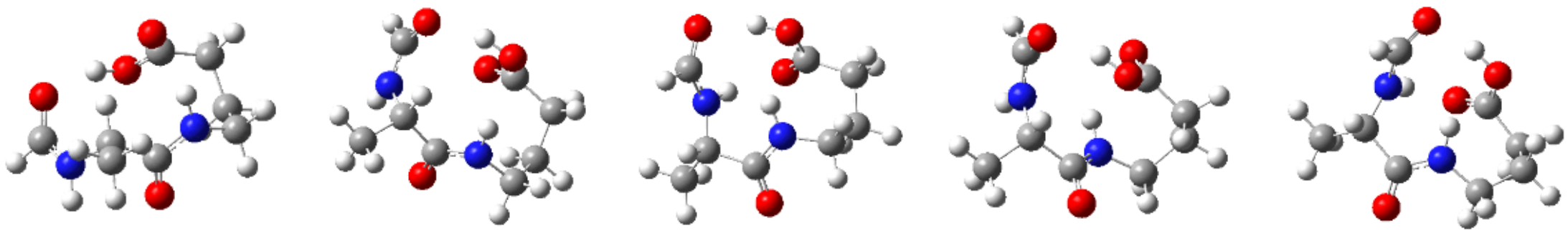

2-c1 **2**-c2 **2**-c3 **2**-c4 **2**-c5

Figure S4 Stable conformers of **2**-3*S* at the B3LYP/6-31+G(d) level in methanol

Table S6 The energies and boltzmann distributions for stable conformers of **2**-3*S*

Conformations	Energy (a.u)	Δ G(kcal/mol)	%
1	-723.594110	0	27.98%
2	-723.594108	0	27.92%
3	-723.594036	0.05	25.87%
4	-723.593203	0.57	10.71%
5	-723.592871	0.78	7.53%

Table S7 Cartesian coordinates of all conformers of compound **2**

Conformer **2**-c1				
Number	Element	Coordinates (Angstroms)		
		X	Y	Z
1	C	0.475014	1.678047	-0.320705
2	C	1.685787	2.587999	-0.080805
3	C	0.248775	0.74534	0.88476
4	N	-0.73139	2.467562	-0.524504
5	C	-1.64717	2.190469	-1.476783
6	O	-1.531009	1.34986	-2.368213
7	O	-0.543656	1.035996	1.774733
8	N	1.019254	-0.376517	0.882401

9	C	0.699789	-1.507072	1.750959
10	C	-0.515149	-2.309134	1.256179
11	C	-0.367937	-2.862993	-0.178427
12	C	-0.331498	-1.762001	-1.222187
13	O	0.67127	-1.420175	-1.830688
14	O	-1.51696	-1.16286	-1.35406
15	H	0.622476	1.086147	-1.227538
16	H	1.835903	3.253763	-0.935438
17	H	2.589493	1.988331	0.063145
18	H	1.536629	3.196374	0.818378
19	H	-1.049218	2.992853	0.281924
20	H	-2.537612	2.839123	-1.409049
21	H	1.471992	-0.610209	0.006204
22	H	1.588851	-2.144484	1.803585
23	H	0.501319	-1.115329	2.750979
24	H	-0.676113	-3.149795	1.941934
25	H	-1.404611	-1.673371	1.308645
26	H	0.549169	-3.451258	-0.278962
27	H	-1.222806	-3.512982	-0.3935
28	H	-1.420582	-0.353653	-1.922988

Conformer **2**-c2

Number	Element	Coordinates (Angstroms)		
		X	Y	Z
1	C	-1.278676	0.364874	-1.331179
2	C	-1.687457	1.047758	-2.637445
3	C	0.090087	0.889717	-0.868601
4	N	-2.241429	0.646087	-0.268777
5	C	-2.772524	-0.316695	0.512556
6	O	-2.634376	-1.529051	0.333877

7	O	0.237954	2.073854	-0.5846
8	N	1.086463	-0.033992	-0.823236
9	C	2.385023	0.27283	-0.2296
10	C	2.367818	0.278625	1.30576
11	C	1.833483	-1.028932	1.921597
12	C	0.327648	-1.123092	1.763762
13	O	-0.443214	-0.315942	2.245341
14	O	-0.063387	-2.163982	1.001616
15	H	-1.272887	-0.719964	-1.451851
16	H	-2.686908	0.722032	-2.939151
17	H	-0.978288	0.798463	-3.43385
18	H	-1.684457	2.134183	-2.507726
19	H	-2.258254	1.601263	0.070077
20	H	-3.390227	0.086813	1.330193
21	H	0.819353	-1.00853	-0.871501
22	H	3.096835	-0.471417	-0.602774
23	H	2.692867	1.253855	-0.59909
24	H	3.390656	0.455402	1.658991
25	H	1.749985	1.111059	1.65587
26	H	2.319276	-1.906725	1.483435
27	H	2.041178	-1.026693	2.997004
28	H	-1.04652	-2.09178	0.869313
Conformer **2**-c3				
Number	Element	Coordinates (Angstroms)		
		X	Y	Z
1	C	-1.23554	0.503197	-1.606636
2	C	-1.844965	1.868438	-1.912963
3	C	0.298168	0.623296	-1.393239
4	N	-1.846084	-0.115057	-0.434339

5	C	-2.417432	-1.333952	-0.40019
6	O	-2.655374	-1.96209	0.63475
7	O	0.91942	1.609028	-1.765329
8	N	0.88754	-0.444596	-0.780962
9	C	2.255119	-0.361432	-0.278279
10	C	2.358292	0.339734	1.085364
11	C	1.687675	-0.418935	2.249227
12	C	0.185699	-0.486786	2.075906
13	O	-0.532862	0.501097	2.046242
14	O	-0.26047	-1.736214	1.891558
15	H	-1.401733	-0.177189	-2.452582
16	H	-2.911545	1.760504	-2.129213
17	H	-1.338883	2.322485	-2.766856
18	H	-1.724533	2.540367	-1.057649
19	H	-1.628463	0.30083	0.476934
20	H	-2.658353	-1.744198	-1.39715
21	H	0.296131	-1.16651	-0.395015
22	H	2.655165	-1.379593	-0.221191
23	H	2.837208	0.193586	-1.016975
24	H	3.420094	0.471265	1.32561
25	H	1.917478	1.338513	1.003319
26	H	2.094537	-1.429727	2.345894
27	H	1.883683	0.121391	3.181696
28	H	-1.239962	-1.737471	1.692067
Conformer **2**-c4				
Number	Element	Coordinates (Angstroms)		
		X	Y	Z
1	C	0.770915	1.639779	-0.368892
2	C	1.587747	2.887585	-0.024986

3	C	1.023113	0.533138	0.674671
4	N	-0.638758	1.971045	-0.571121
5	C	-1.301261	1.768914	-1.737551
6	O	-0.854952	1.179714	-2.717018
7	O	2.175649	0.245016	0.974294
8	N	-0.077441	-0.067946	1.20415
9	C	0.027938	-1.284949	2.001834
10	C	-0.647253	-2.491767	1.332729
11	C	-0.186358	-2.713405	-0.113873
12	C	-0.768898	-1.660499	-1.038921
13	O	-1.887267	-1.189473	-0.909091
14	O	0.083732	-1.28423	-1.999687
15	H	1.116521	1.226338	-1.32007
16	H	1.54299	3.611885	-0.843364
17	H	2.625153	2.598802	0.152358
18	H	1.208244	3.364627	0.887236
19	H	-1.087382	2.561778	0.118456
20	H	-2.316987	2.198776	-1.730283
21	H	-0.971577	0.137371	0.776943
22	H	1.097888	-1.461237	2.1355
23	H	-0.410441	-1.117822	2.993514
24	H	-0.426795	-3.380448	1.9351
25	H	-1.735185	-2.363384	1.331134
26	H	0.904257	-2.70766	-0.199021
27	H	-0.537752	-3.690654	-0.46902
28	H	-0.315842	-0.511338	-2.475013
Conformer **2**-c5				
Number	Element	Coordinates (Angstroms)		
		X	Y	Z

1	C	-1.924078	-0.853855	-0.239937
2	C	-3.025493	-0.547842	-1.251398
3	C	-0.578813	-1.13467	-0.965081
4	N	-1.756841	0.228797	0.723235
5	C	-1.779411	0.097248	2.0633
6	O	-1.338181	0.936594	2.852547
7	O	-0.54434	-1.42316	-2.153134
8	N	0.533799	-1.037151	-0.183876
9	C	1.882583	-1.100608	-0.732028
10	C	2.808557	-0.051709	-0.105419
11	C	2.349982	1.40626	-0.299939
12	C	1.06116	1.741355	0.420757
13	O	0.091423	2.256564	-0.112221
14	O	1.092535	1.383929	1.713572
15	H	-2.185232	-1.754971	0.331423
16	H	-3.982358	-0.427438	-0.735709
17	H	-3.098216	-1.354926	-1.982469
18	H	-2.798486	0.374955	-1.794126
19	H	-1.367633	1.107941	0.369061
20	H	-2.229673	-0.849289	2.412733
21	H	0.432858	-0.766293	0.783833
22	H	2.311092	-2.100367	-0.580506
23	H	1.785332	-0.951827	-1.811016
24	H	2.928301	-0.248338	0.966168
25	H	3.799967	-0.170184	-0.557655
26	H	3.130295	2.074388	0.085226
27	H	2.199127	1.637581	-1.357542
28	H	0.201746	1.527026	2.140211

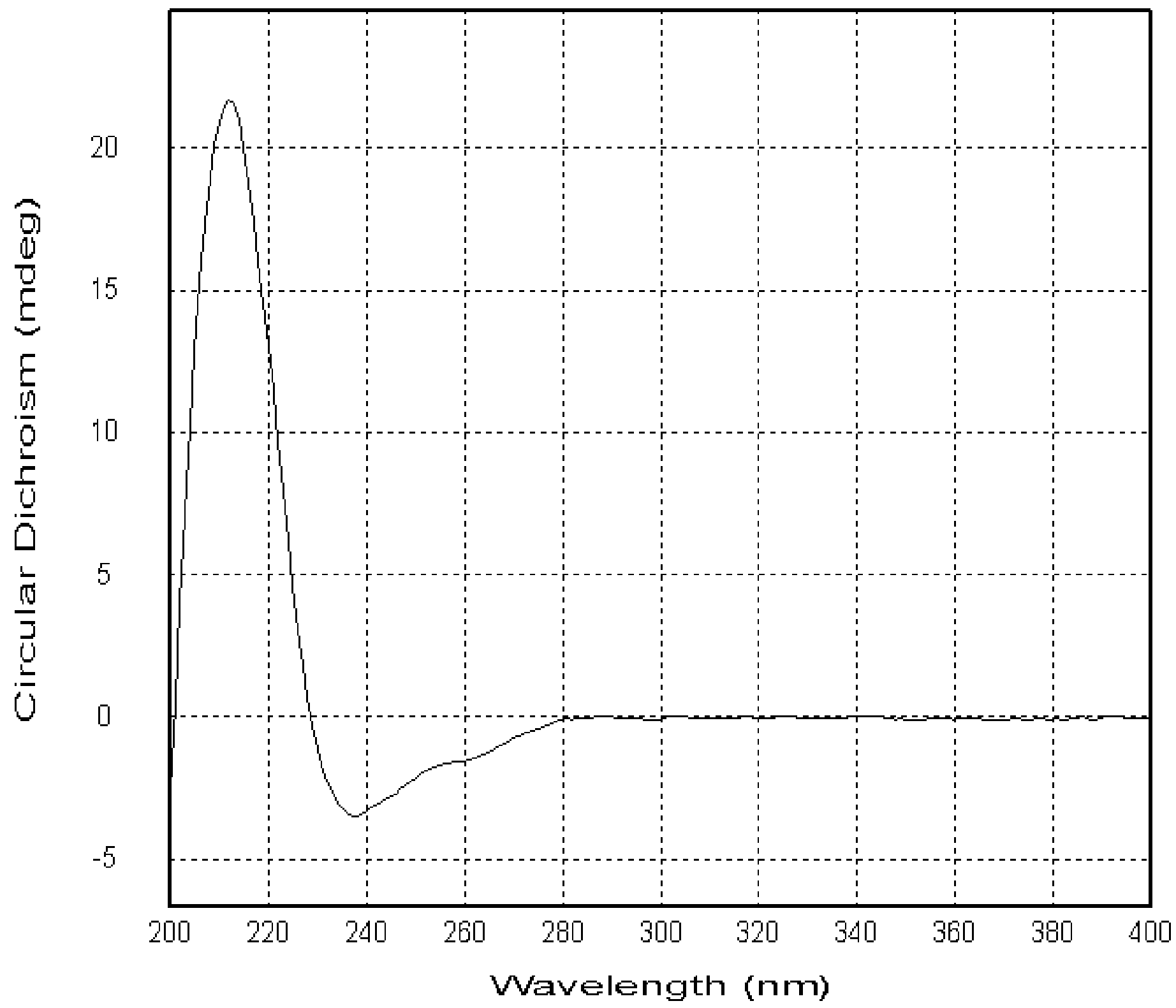

Figure S5 ECD spectrum of **1**

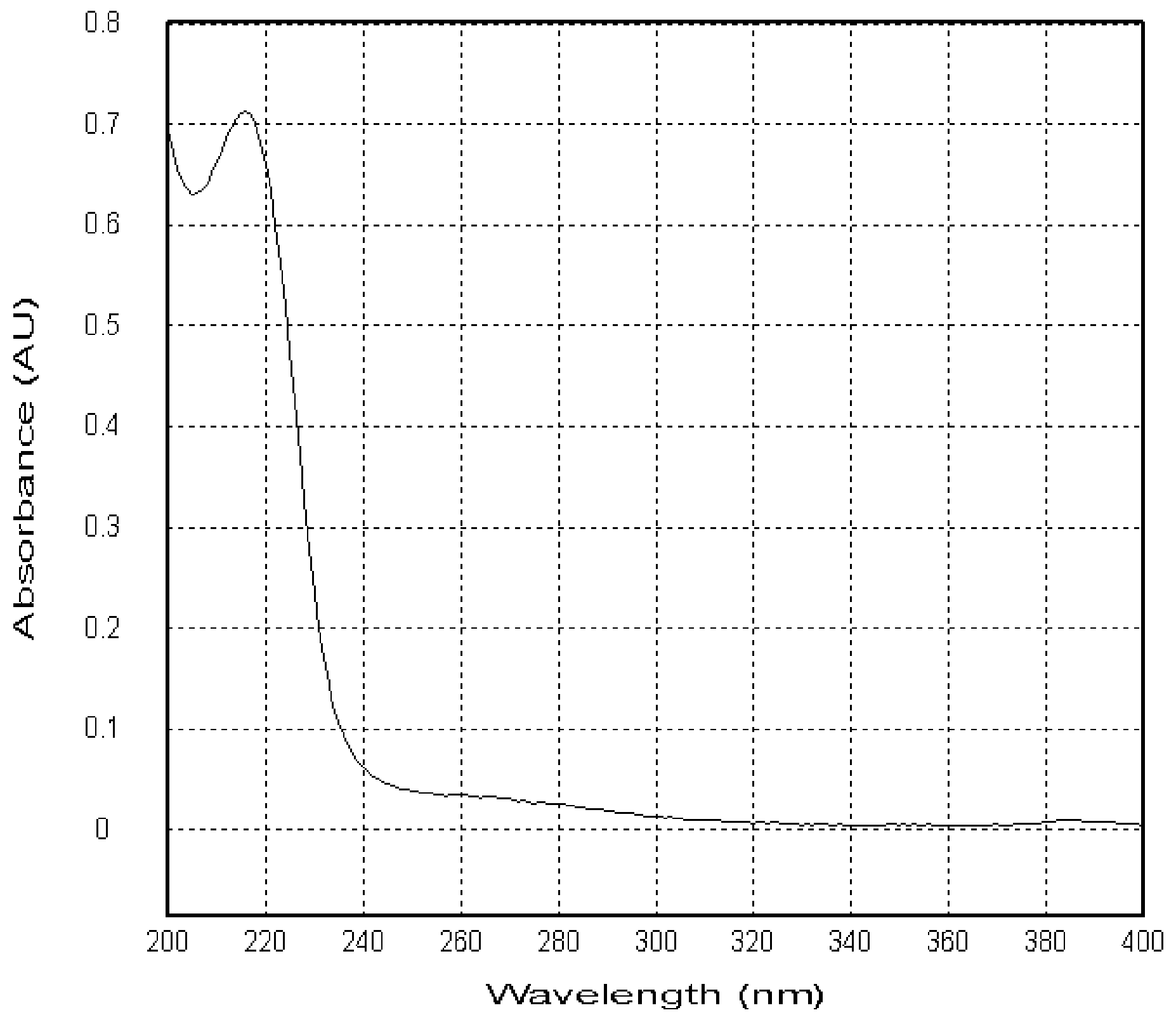

Figure S6 UV spectrum of **1**

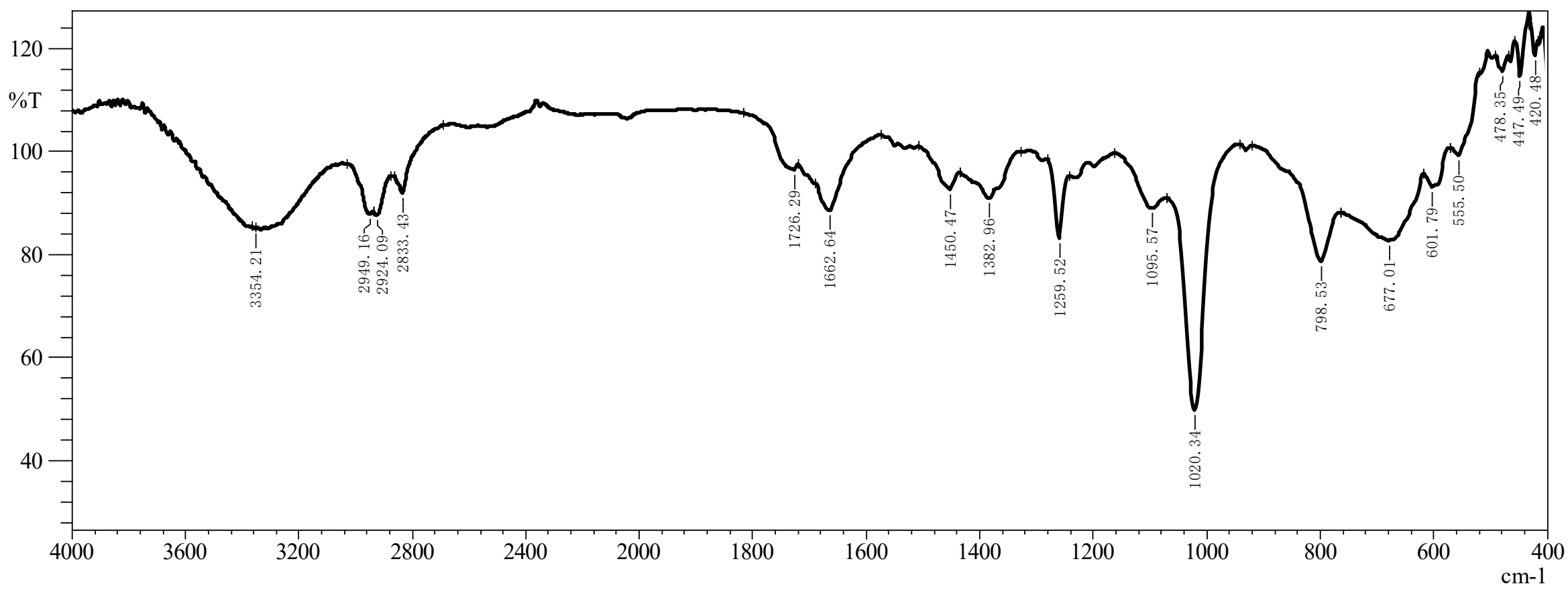

Figure S7 IR spectrum of **1**

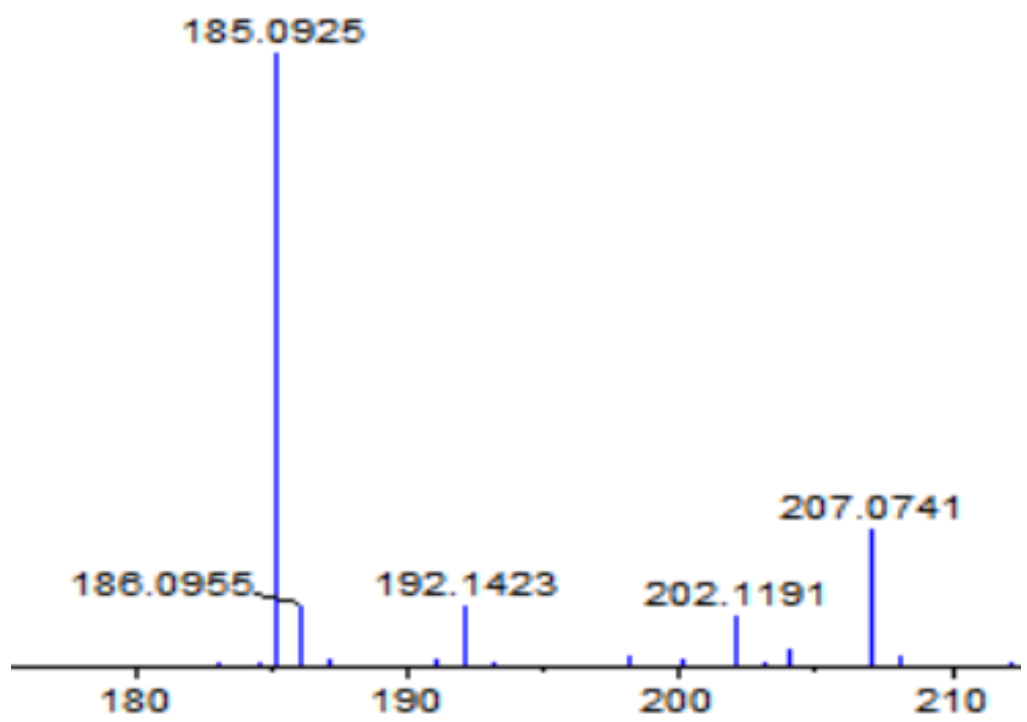

Figure S8 HRESIMS spectrum of **1**

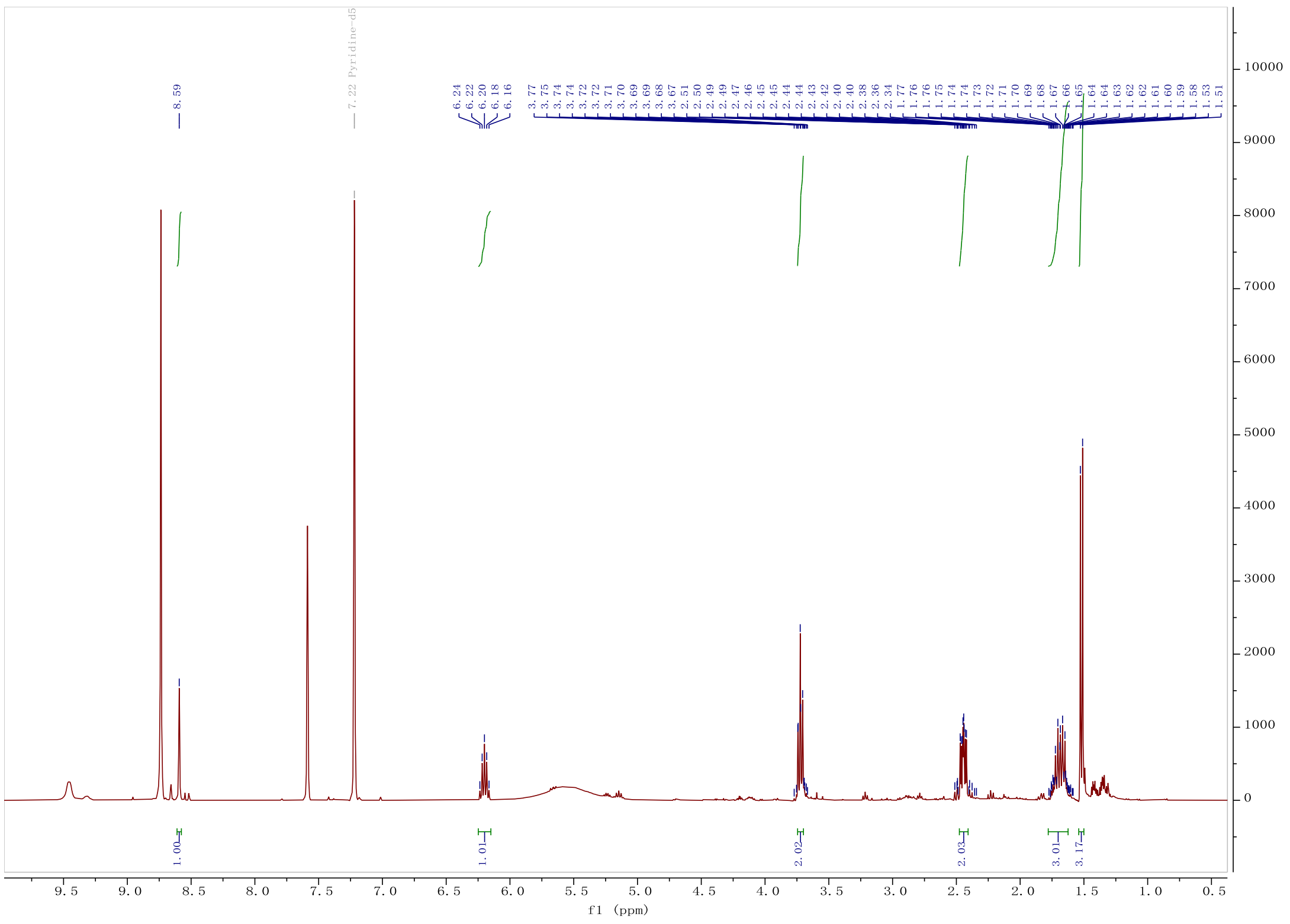

Figure S9 ^{1}H NMR (400MHz) spectrum of **1** in Pyridine-d_5

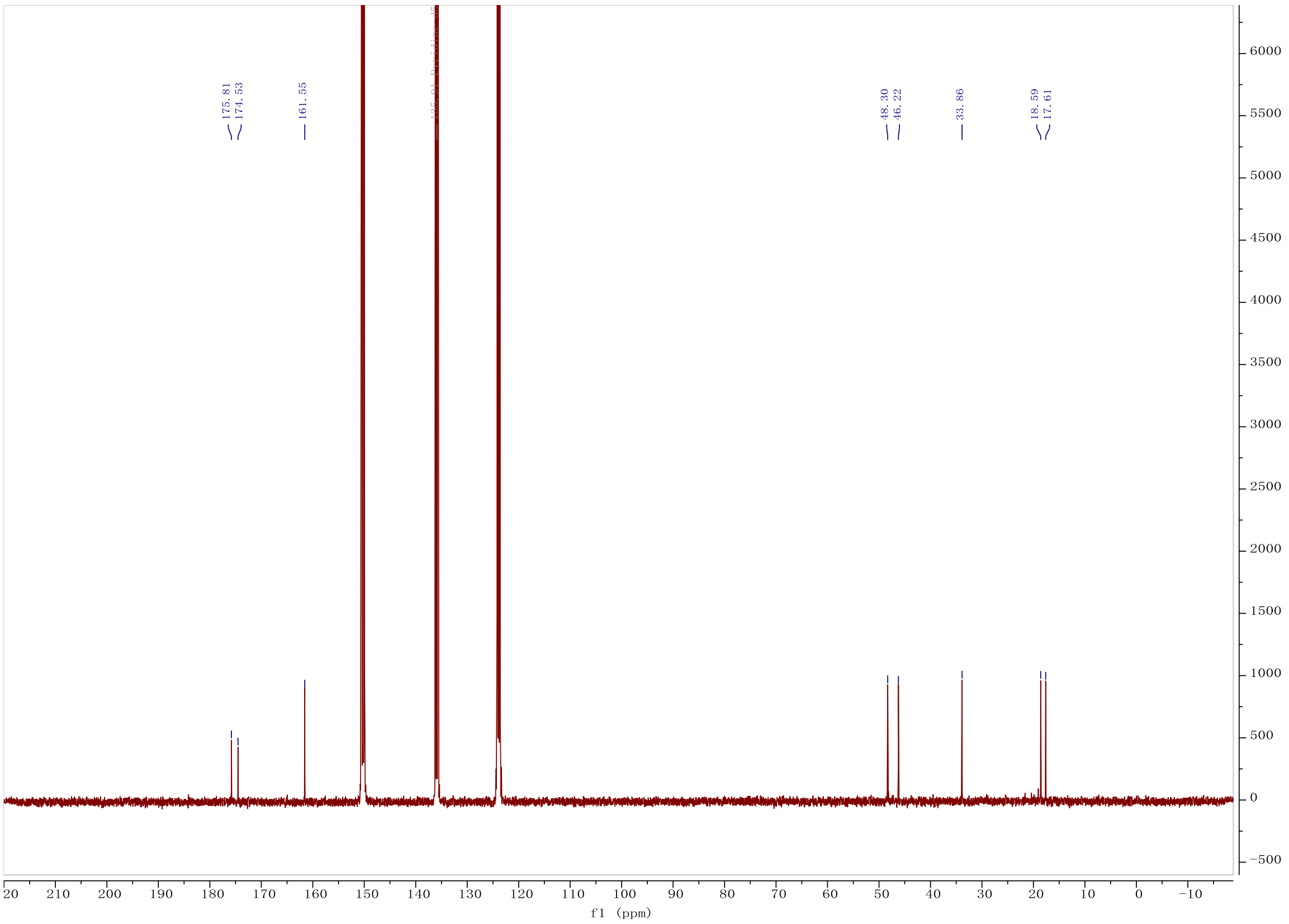

Figure S10 ^{13}C NMR (100MHz) spectrum of **1** in Pyridine-d_5

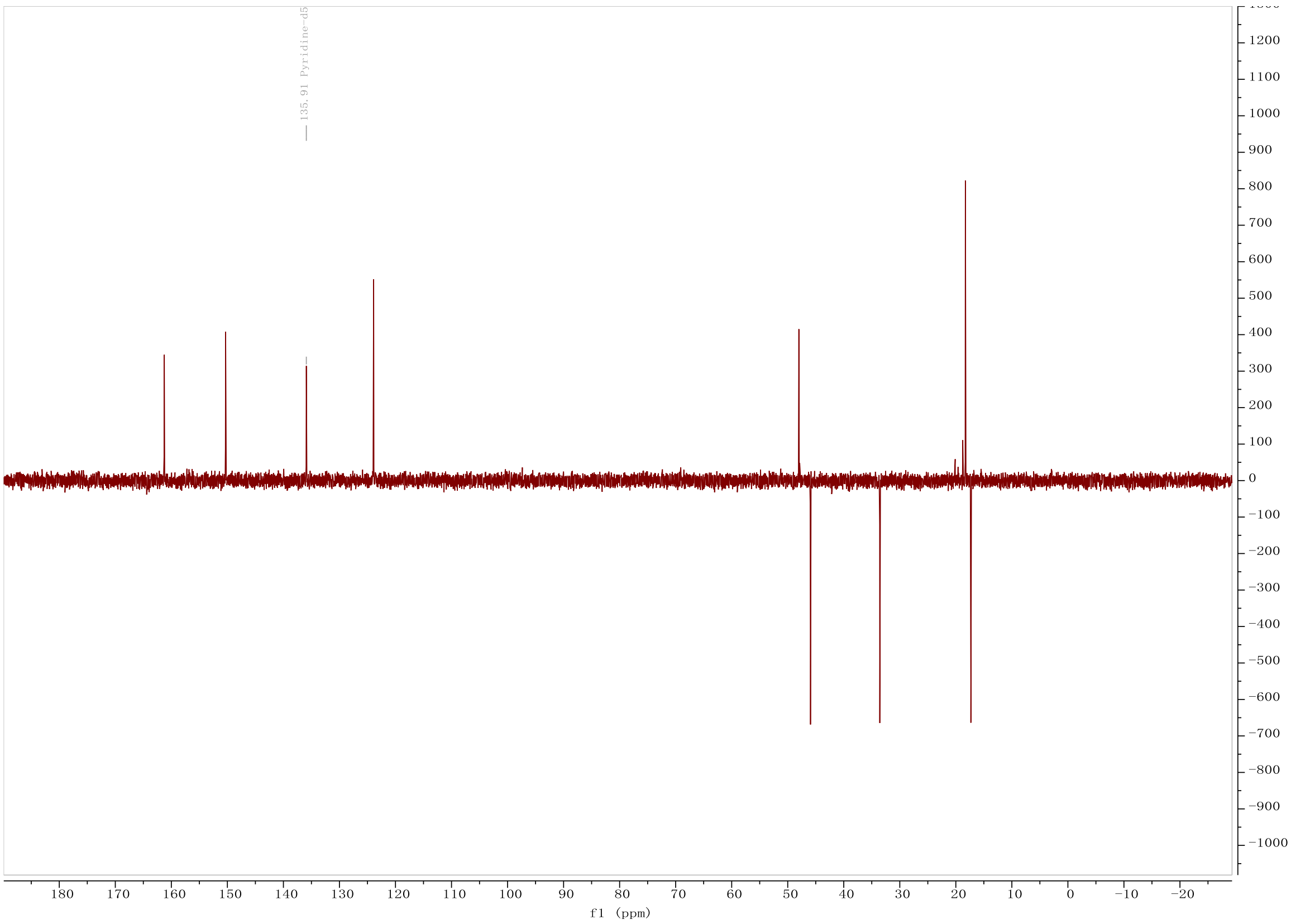

Figure S11 DEPT 135 (100MHz) spectrum of compound **1** in Pyridine-d_5

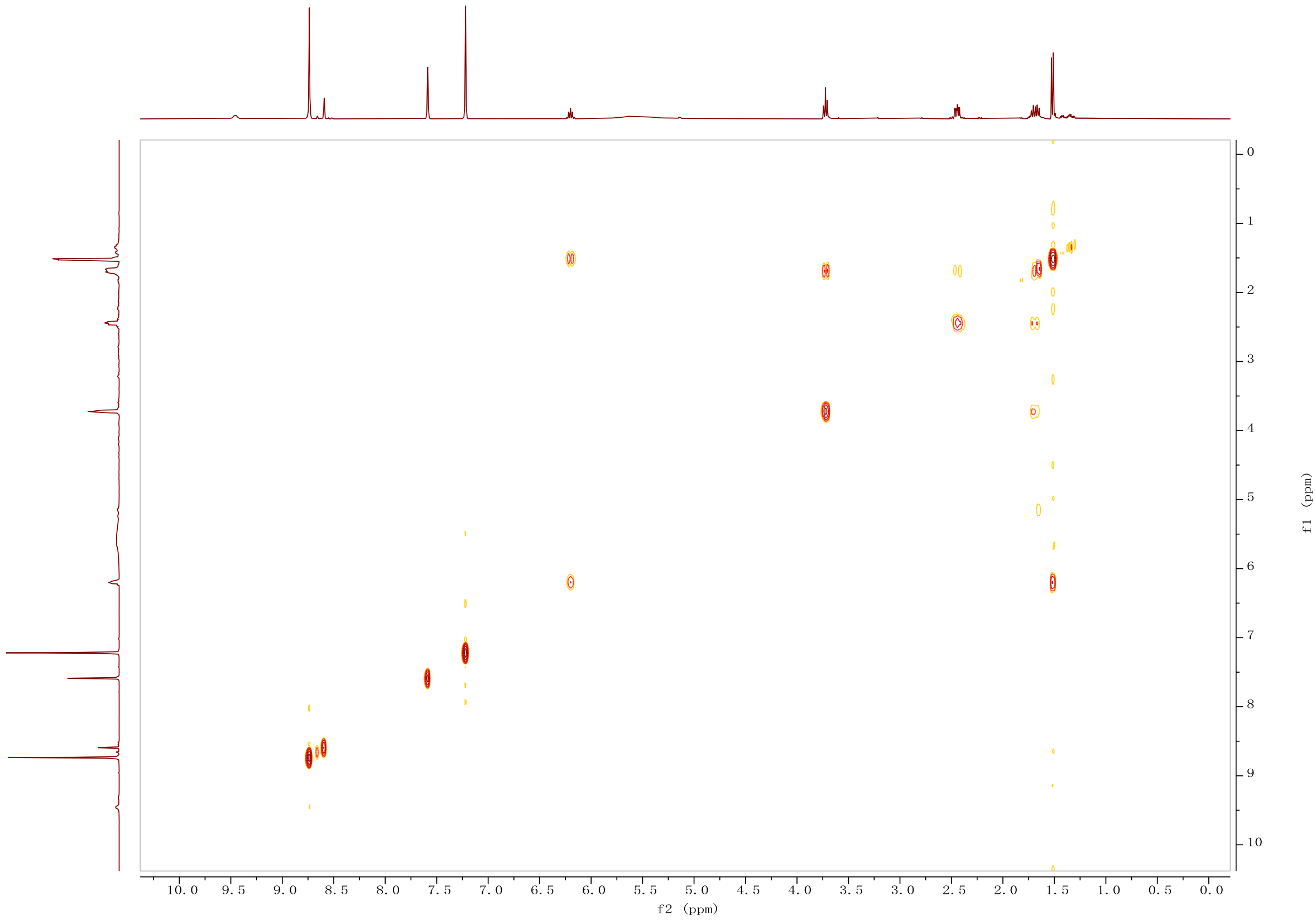

Figure S12 ^{1}H-^{1}H COSY spectrum of **1** in Pyridine-d_5

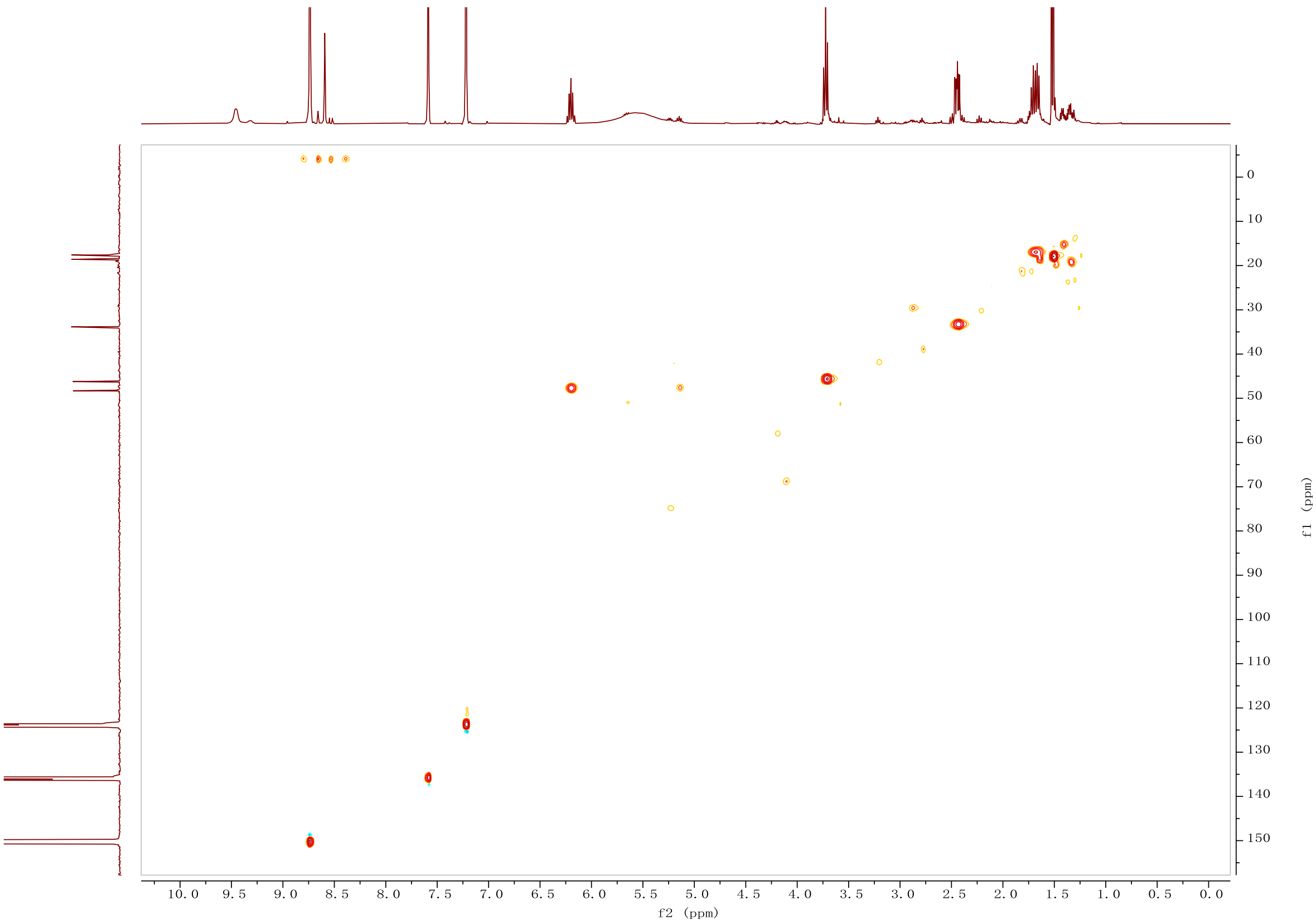

Figure S13 HSQC spectrum of **1** in Pyridine-d_5

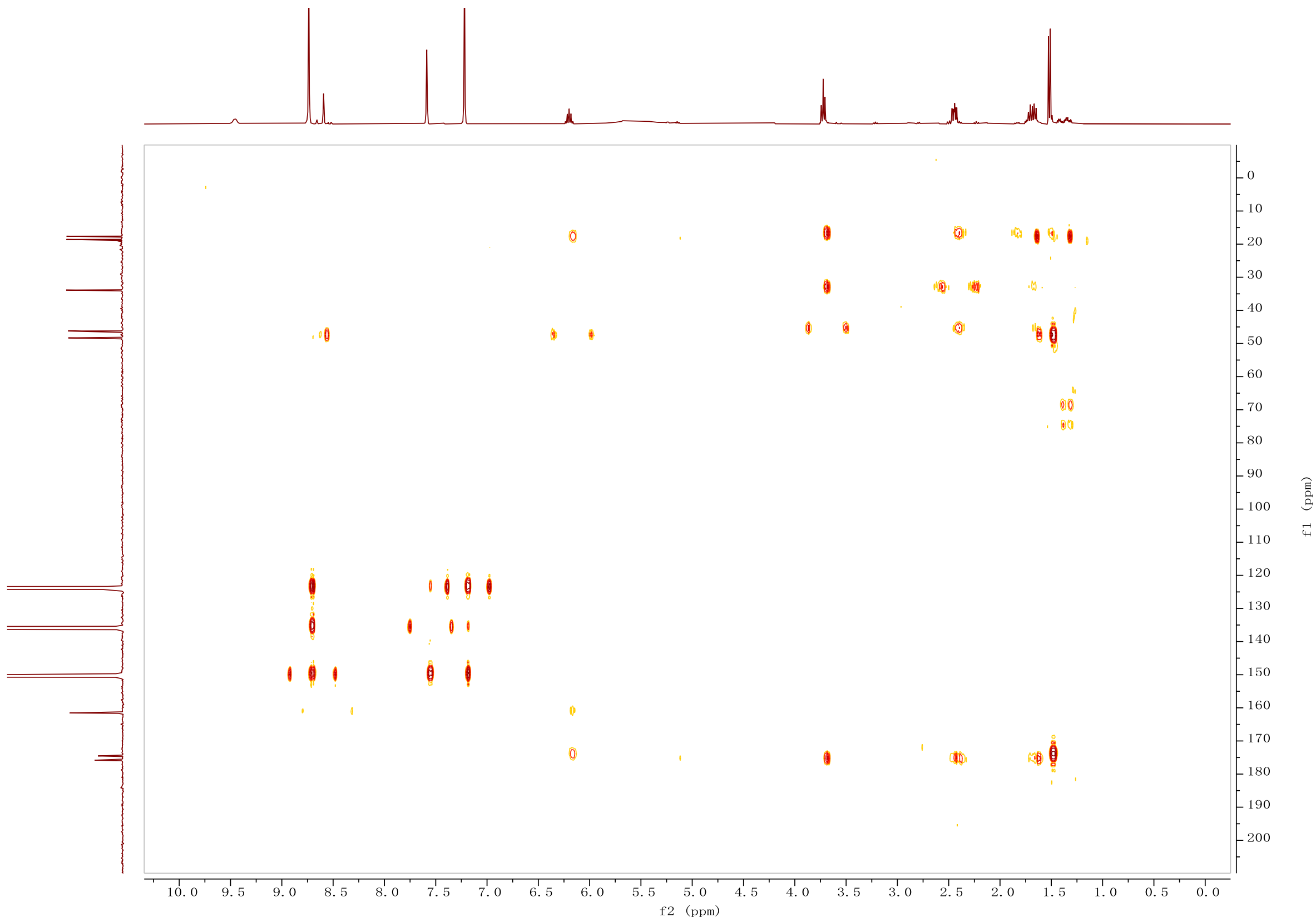

Figure S14 HMBC spectrum of **1** in Pyridine-d_5

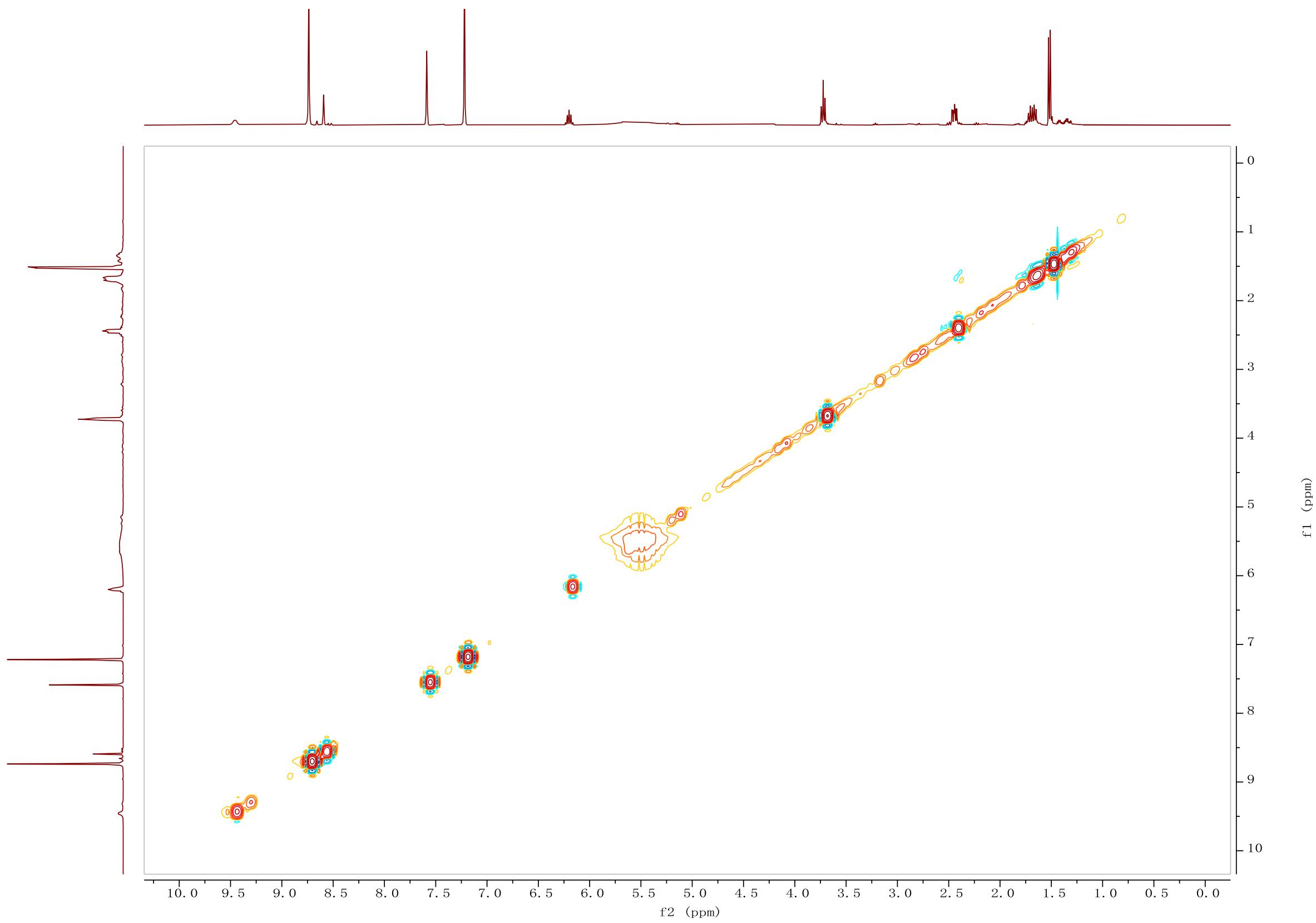

Figure S15 NOESY spectrum of **1** in Pyridine-d_5

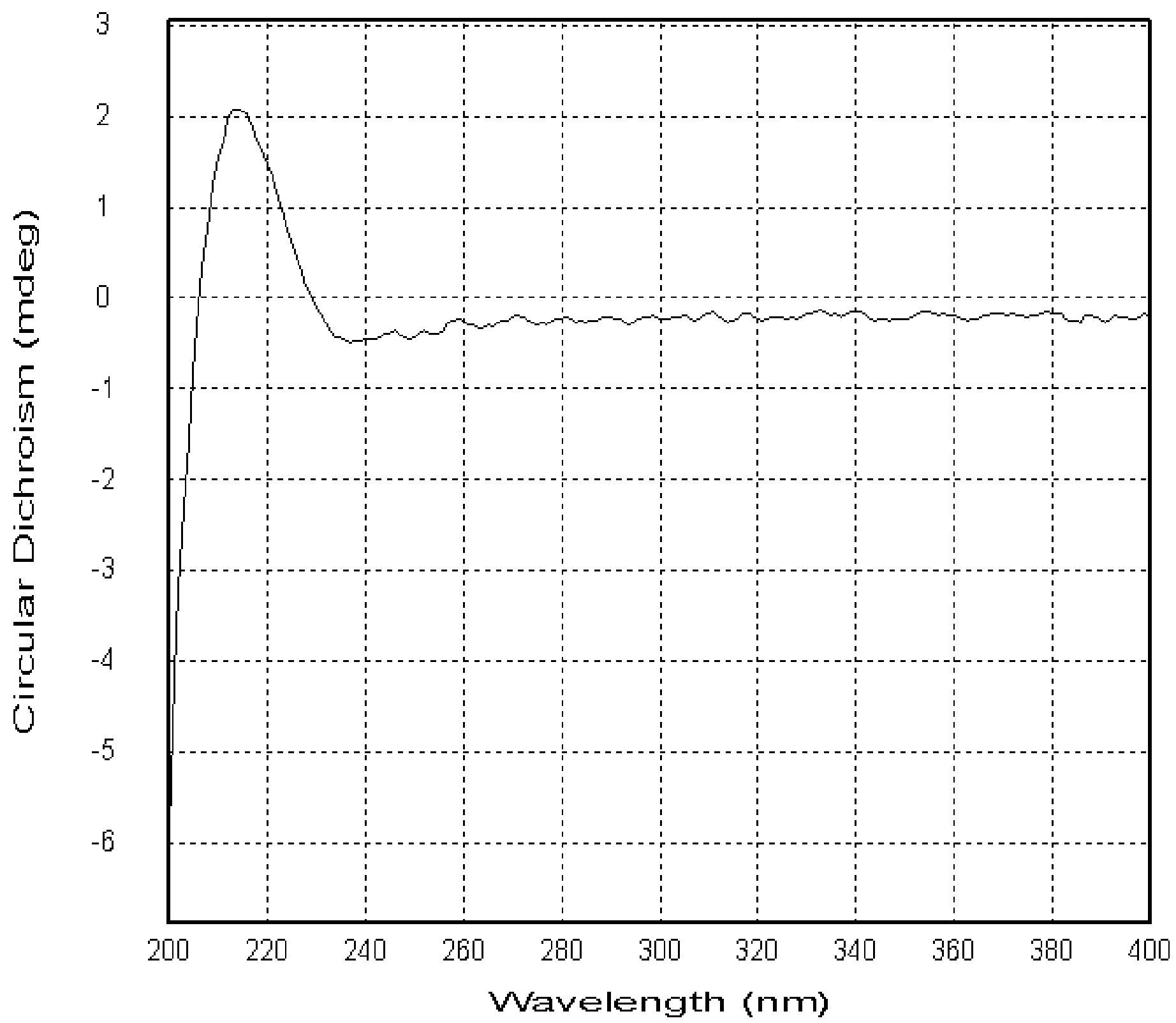

Figure S16 ECD spectrum of **2**

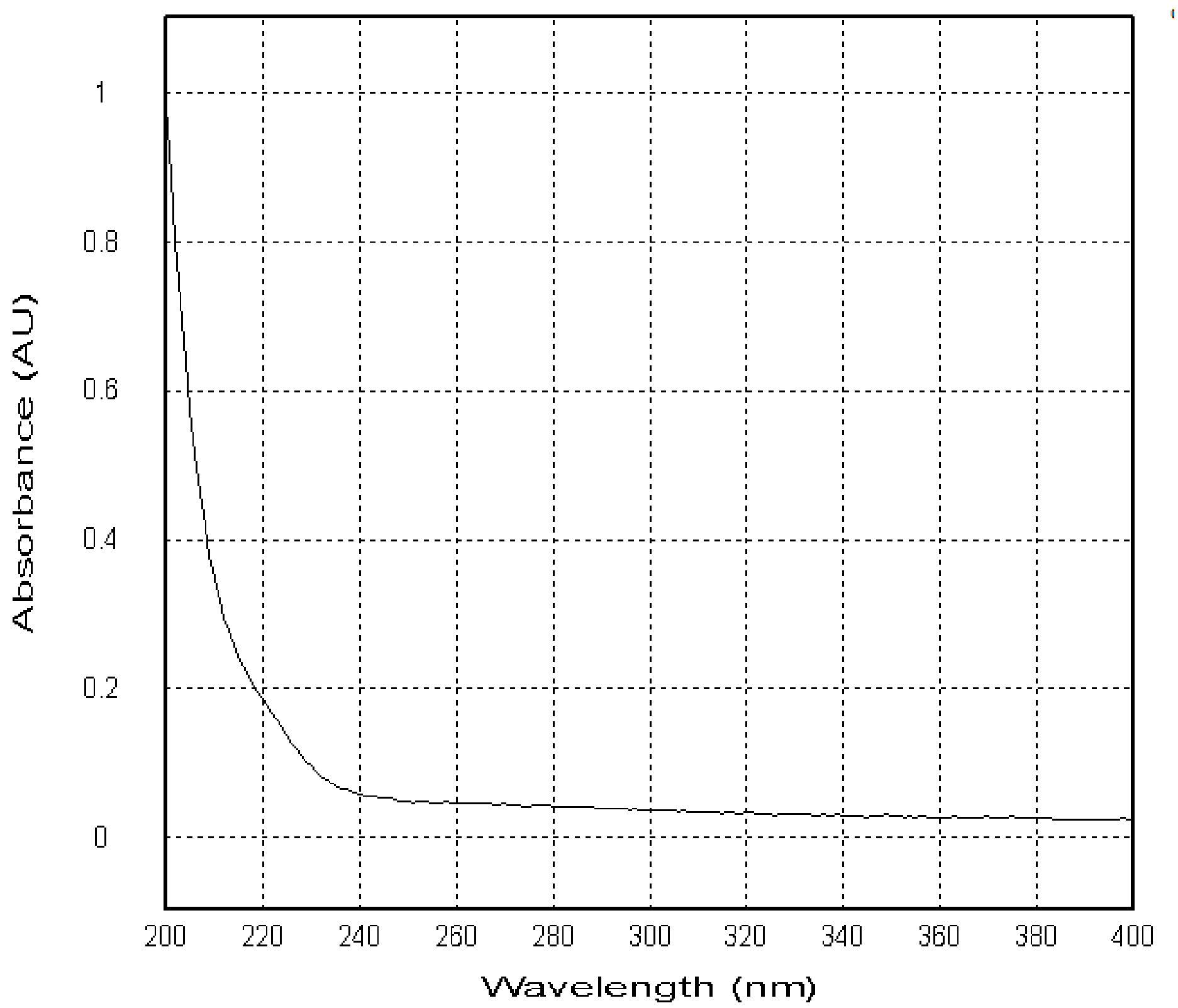

Figure S17 UV spectrum of **2**

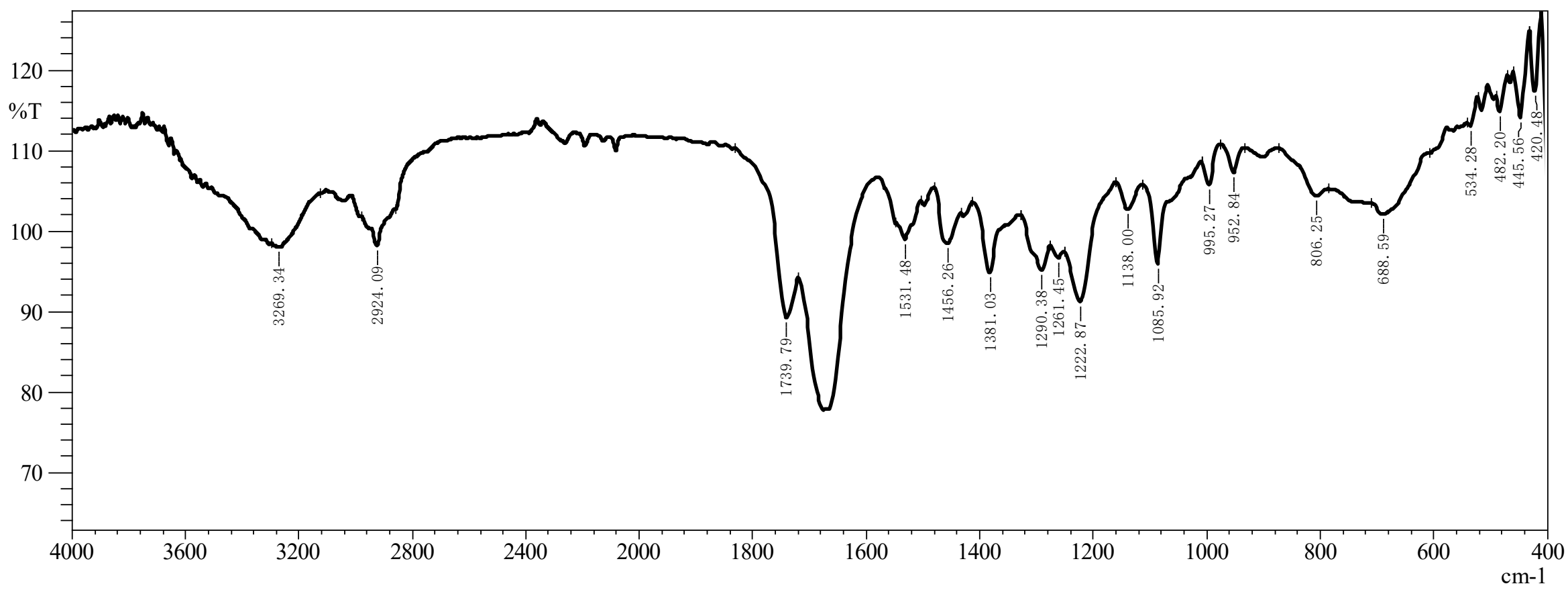

Figure S18 IR spectrum of **2**

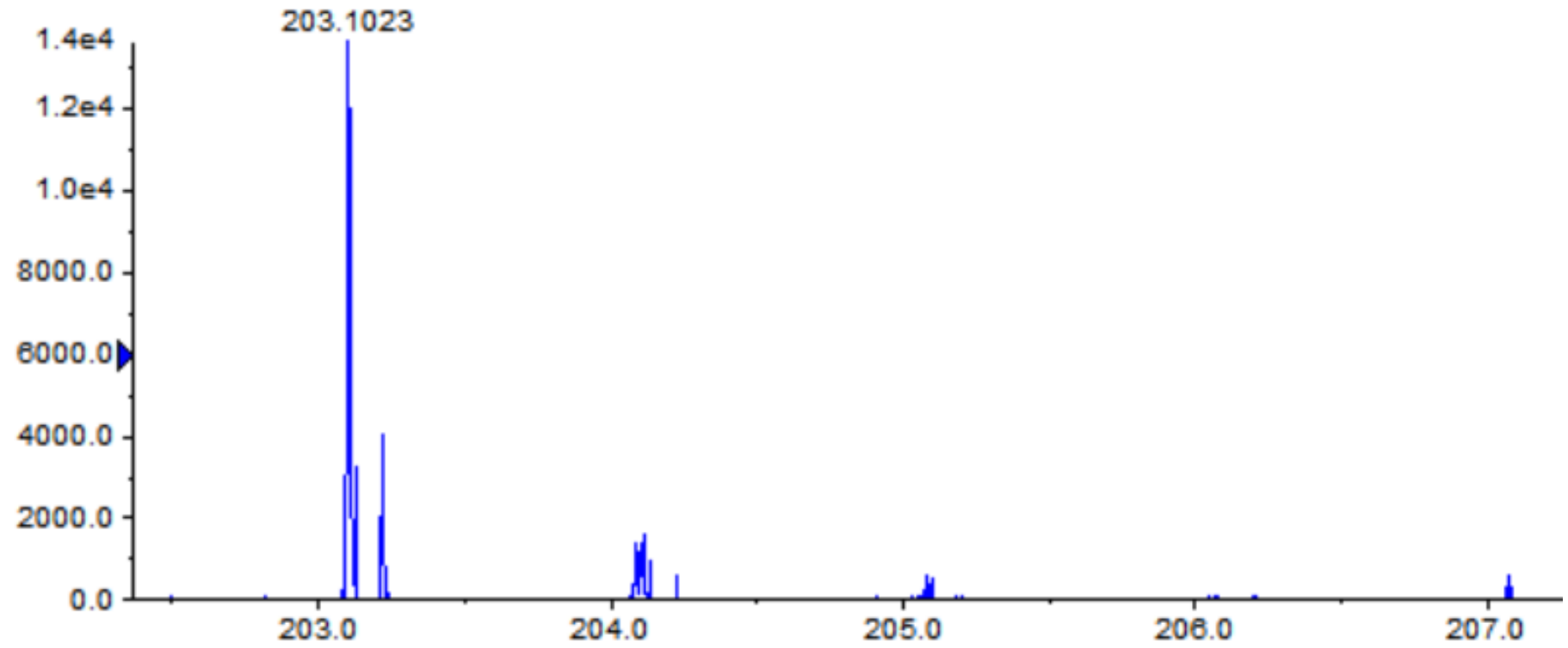

Figure S19 HRESIMS spectrum of **2**

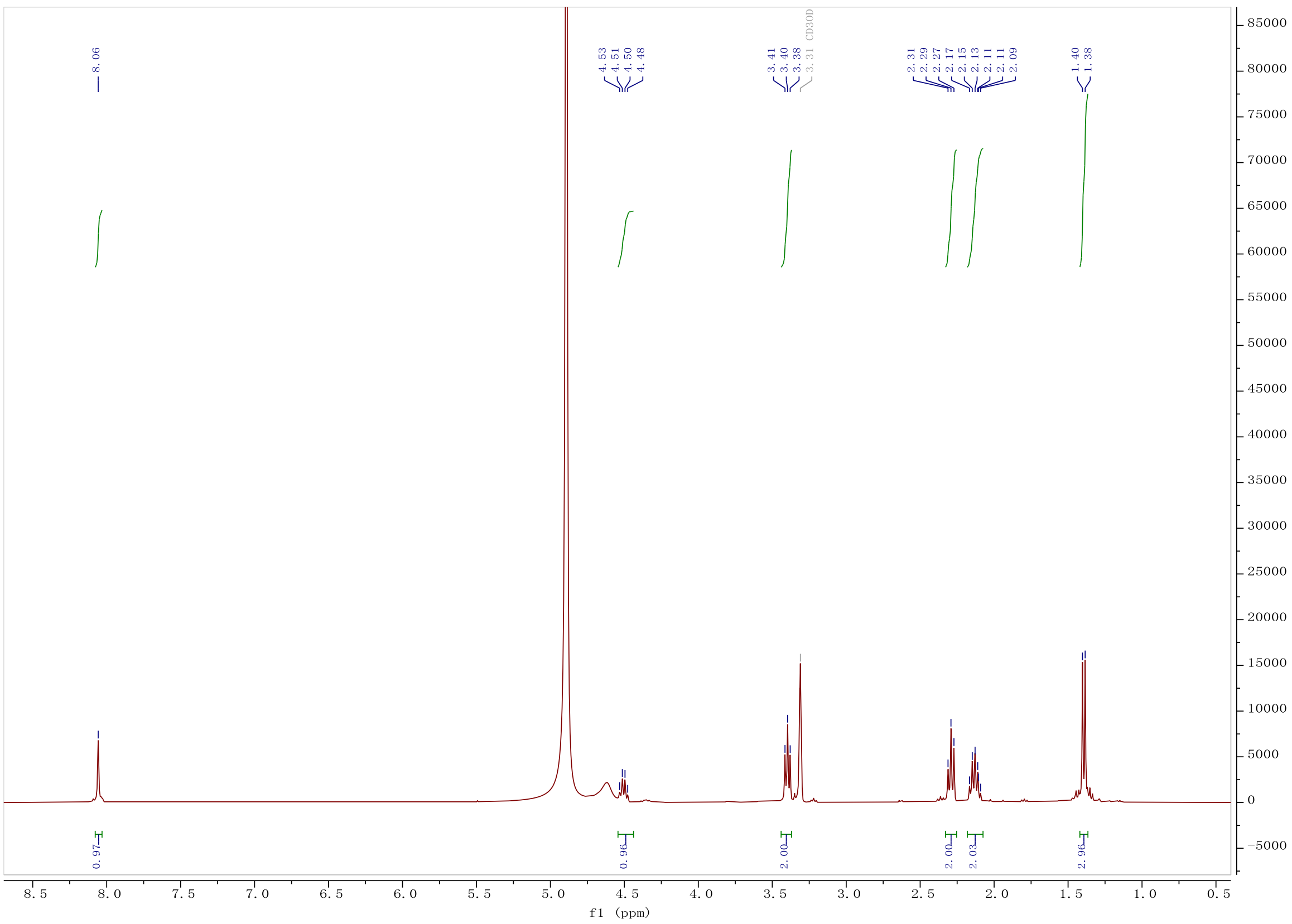

Figure S20 ^{1}H NMR (400MHz) spectrum of **2** in CD_3OD

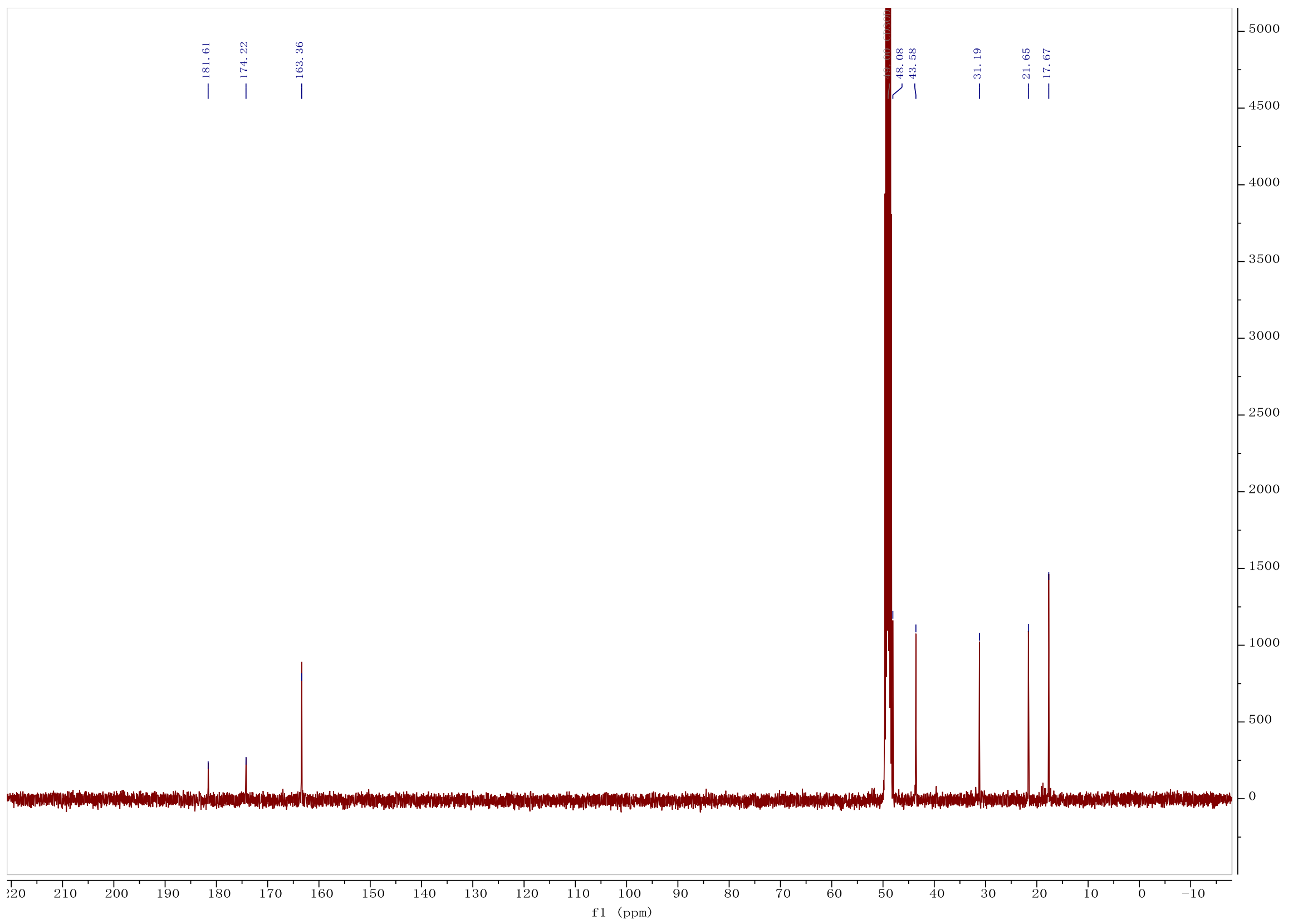

Figure S21 ^{13}C NMR (100MHz) spectrum of **2** in CD_3OD

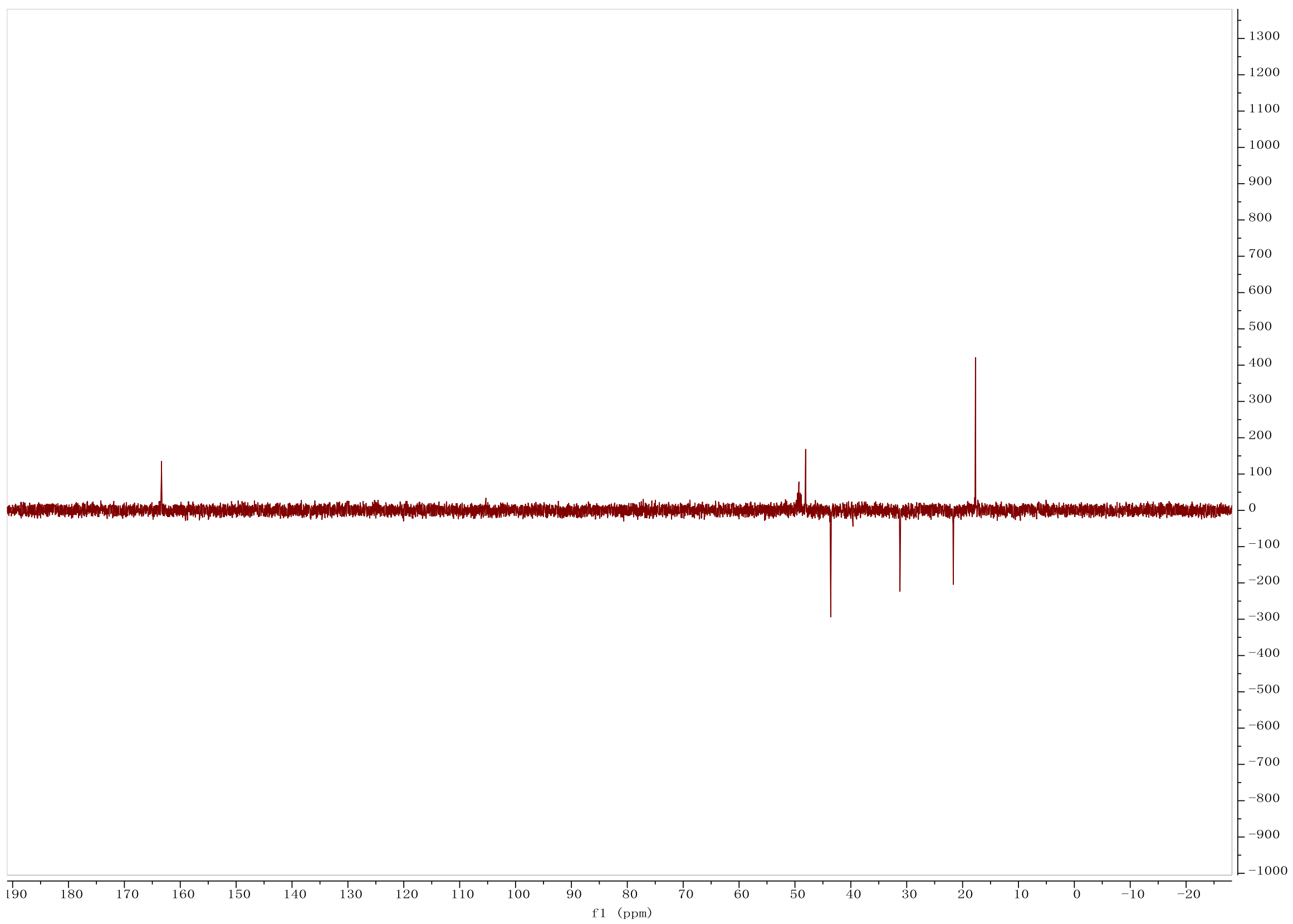

Figure S22 DEPT 135 (100MHz) spectrum of compound **2** in CD_3OD

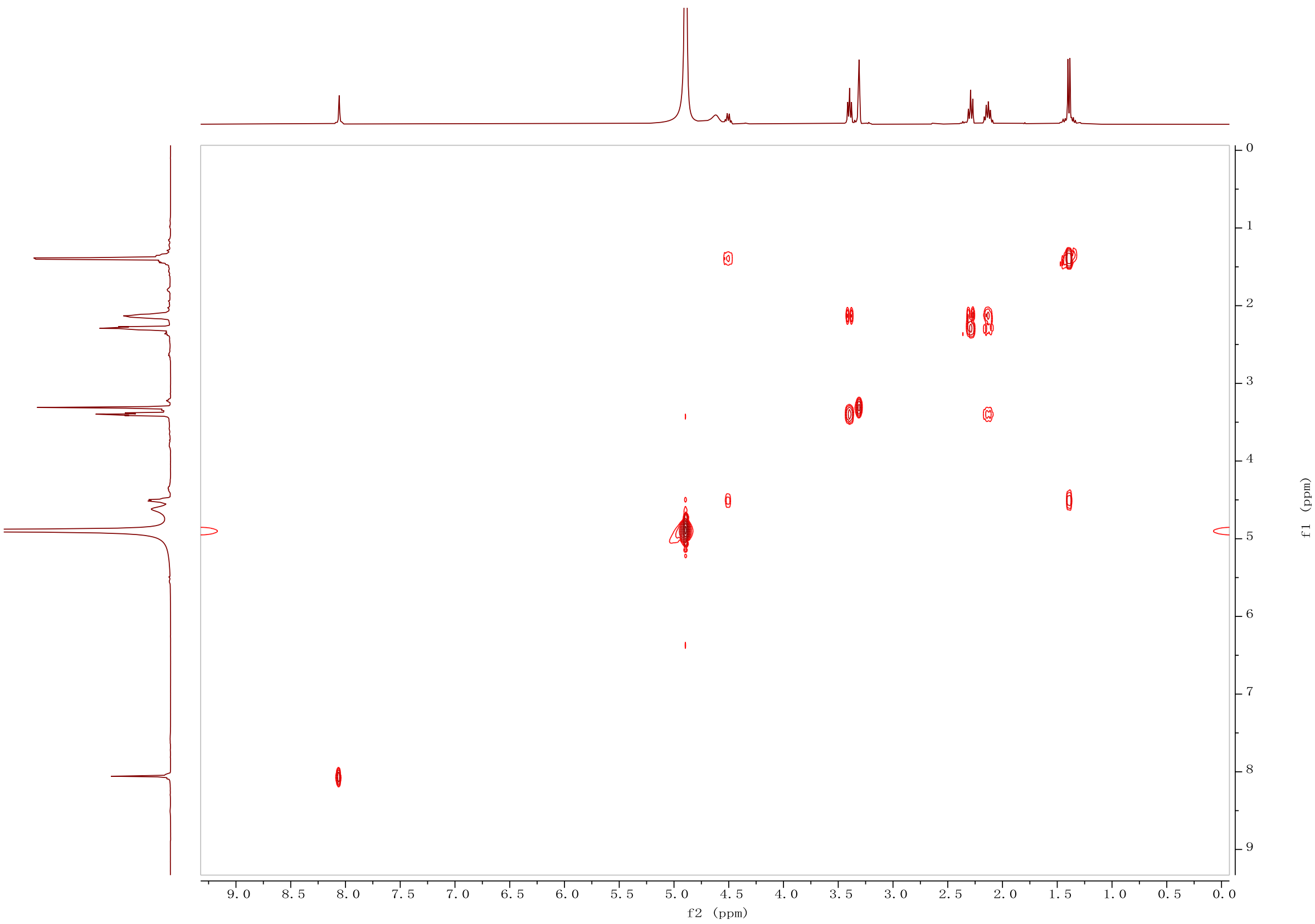

Figure S23 ^{1}H-^{1}H COSY spectrum of **2** in CD_3OD

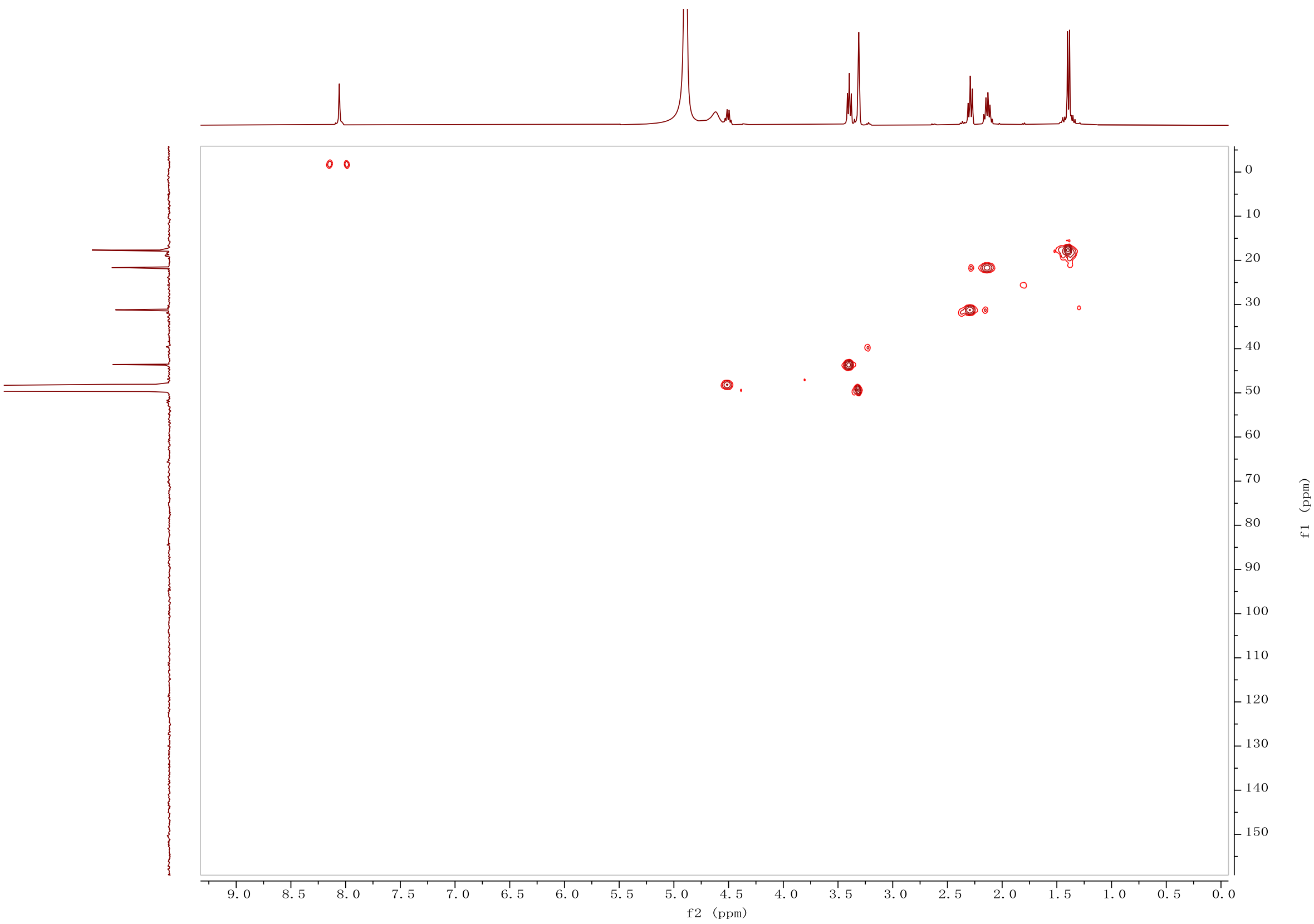

Figure S24 HSQC spectrum of **2** in CD_3OD

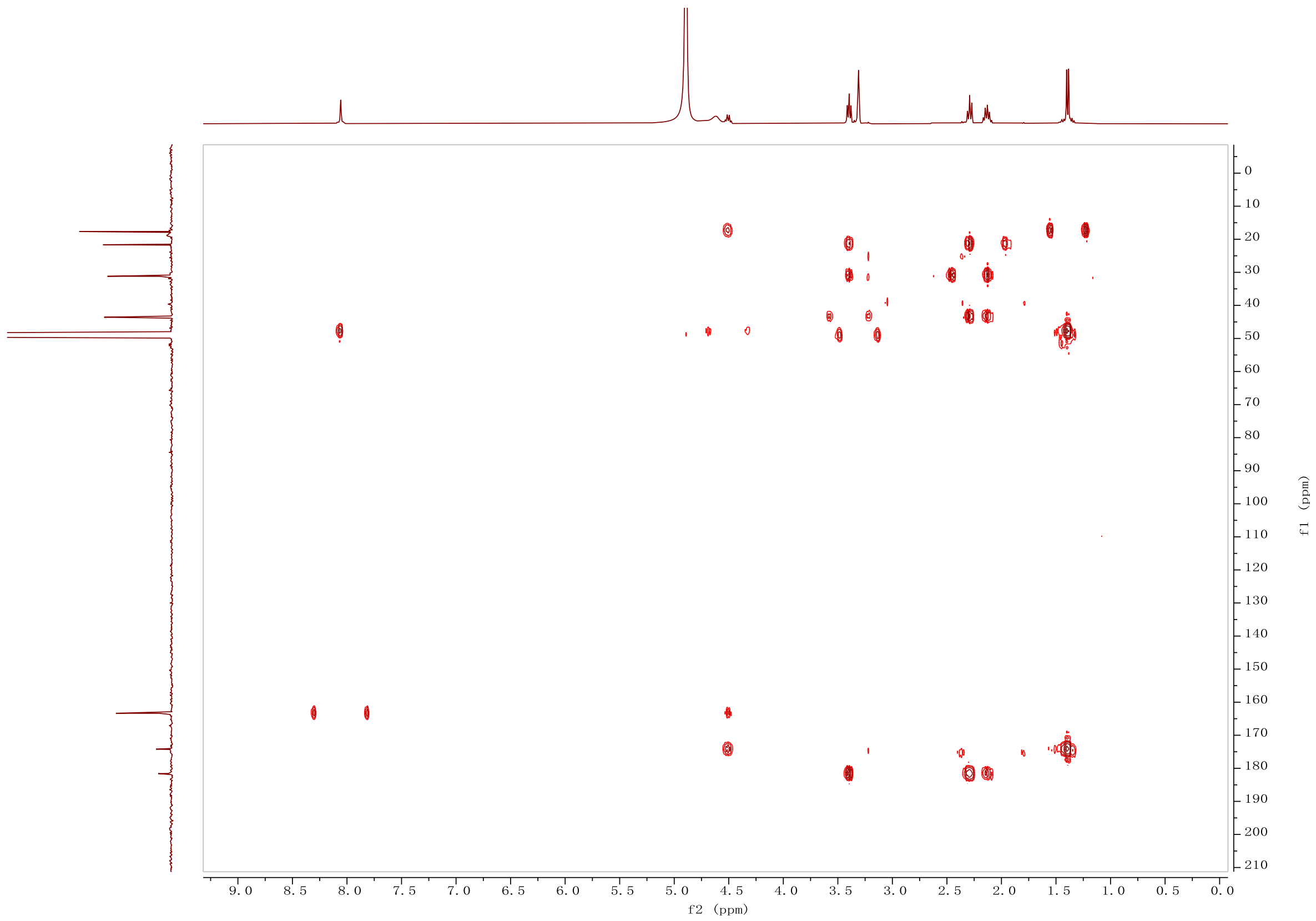

Figure S25 HMBC spectrum of **2** in CD_3OD

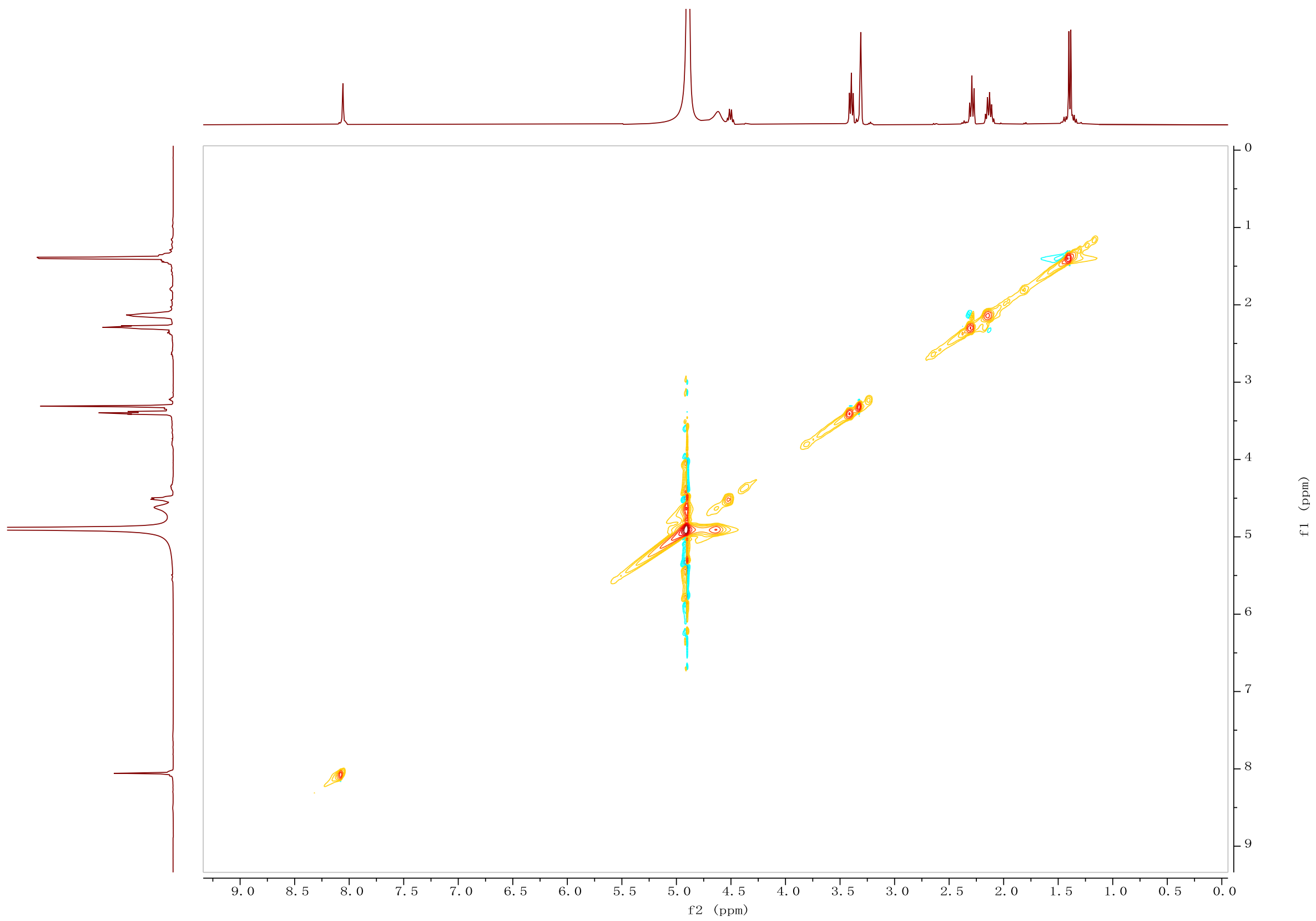

Figure S26 NOESY spectrum of **2**

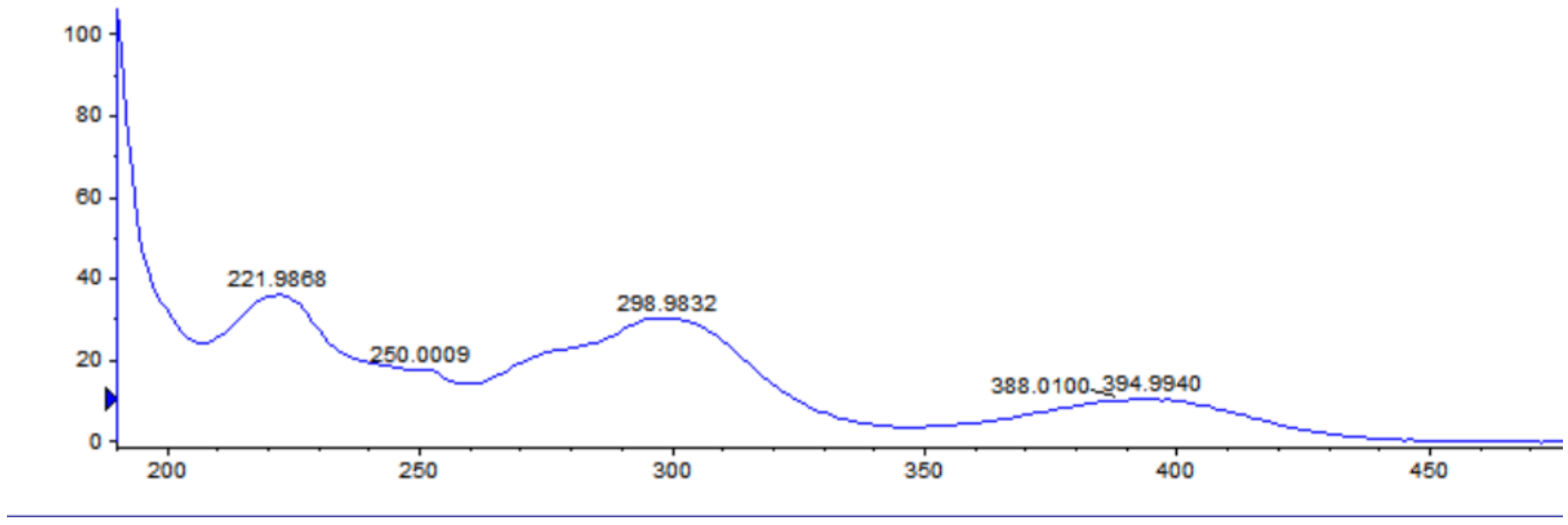

Figure S27 UV spectrum of **3**

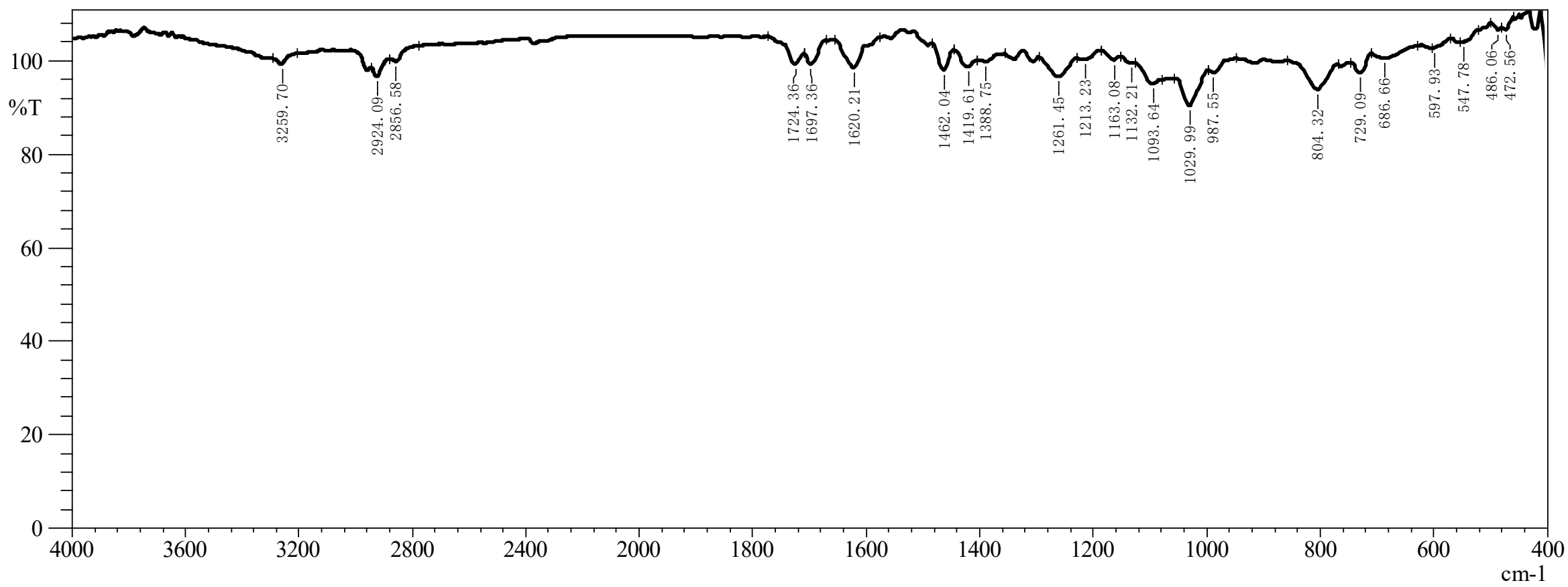

Figure S28 IR spectrum of **3**

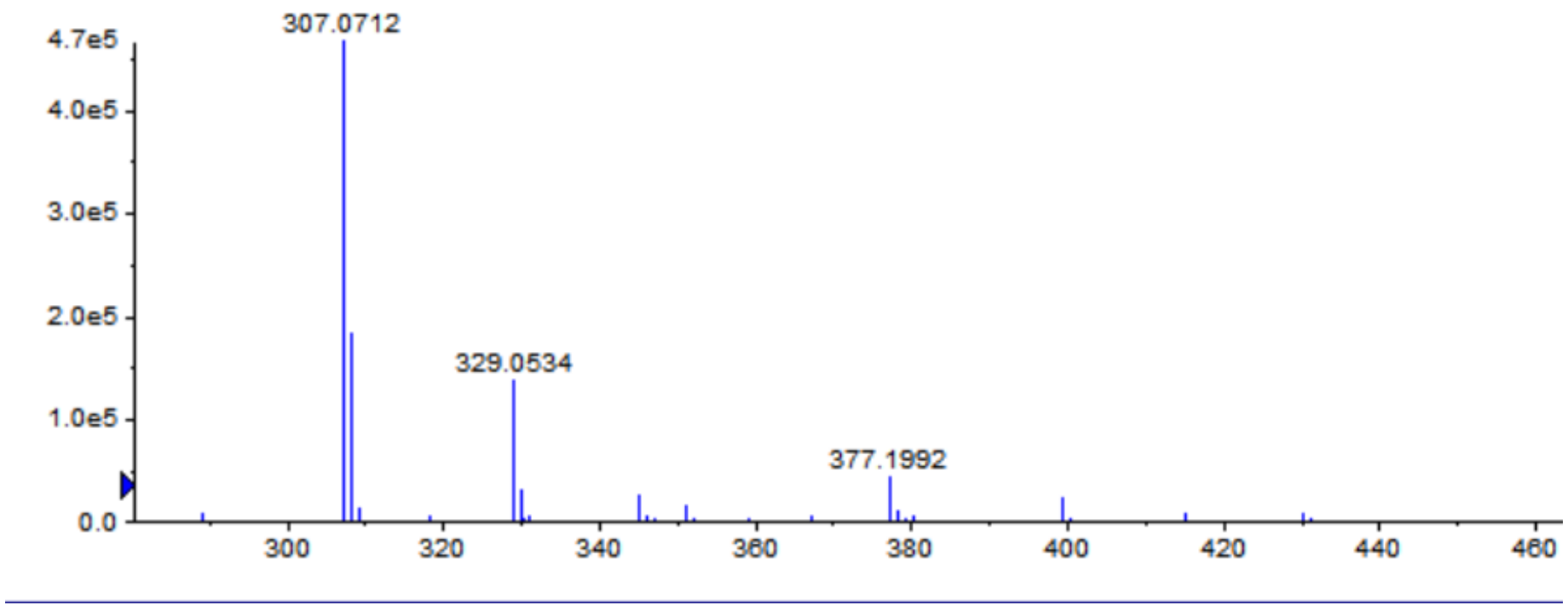

Figure S29 HRESIMS spectrum of **3**

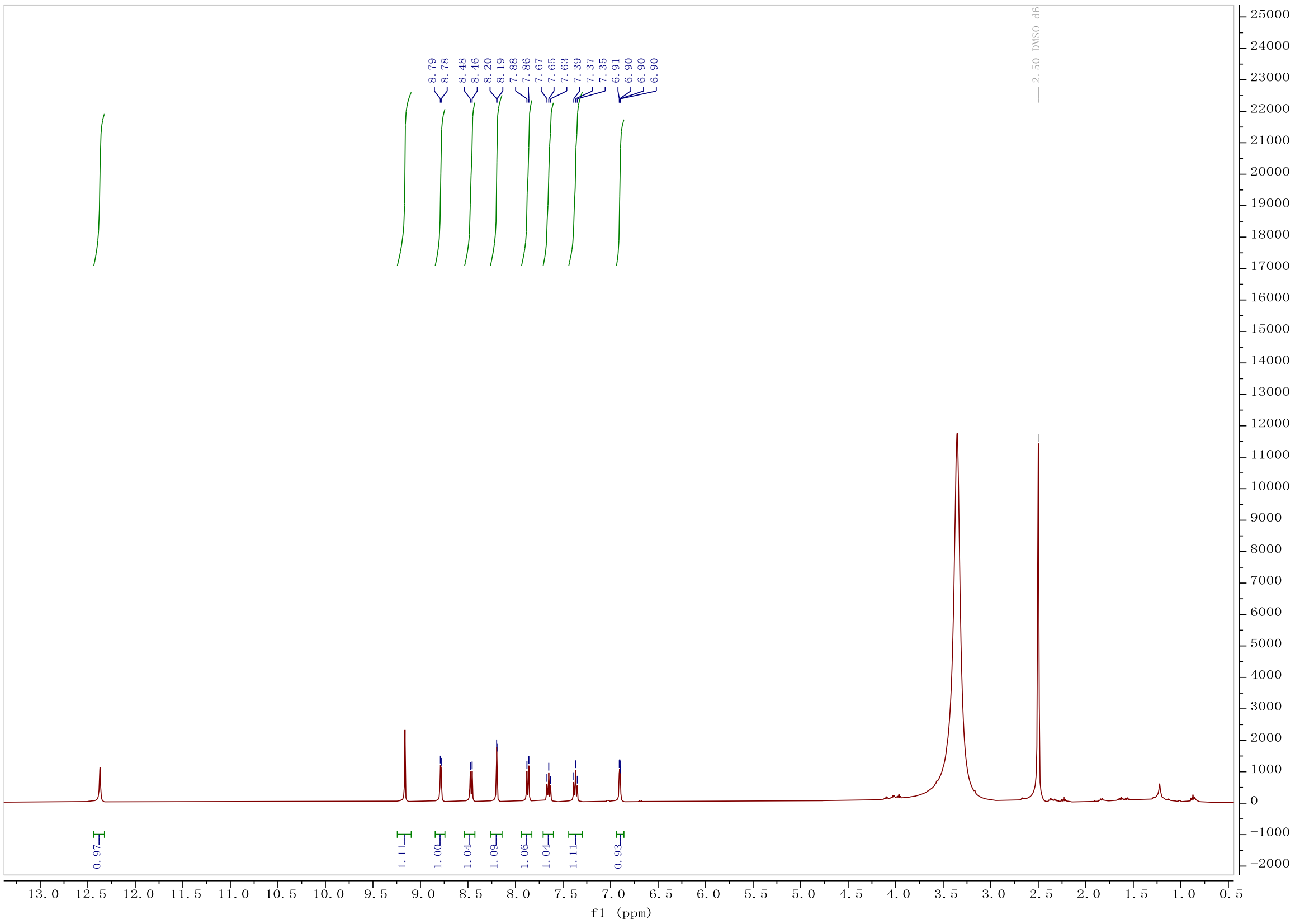

Figure S30 ^{1}H NMR (400MHz) spectrum of **3** in DMSO-d_6

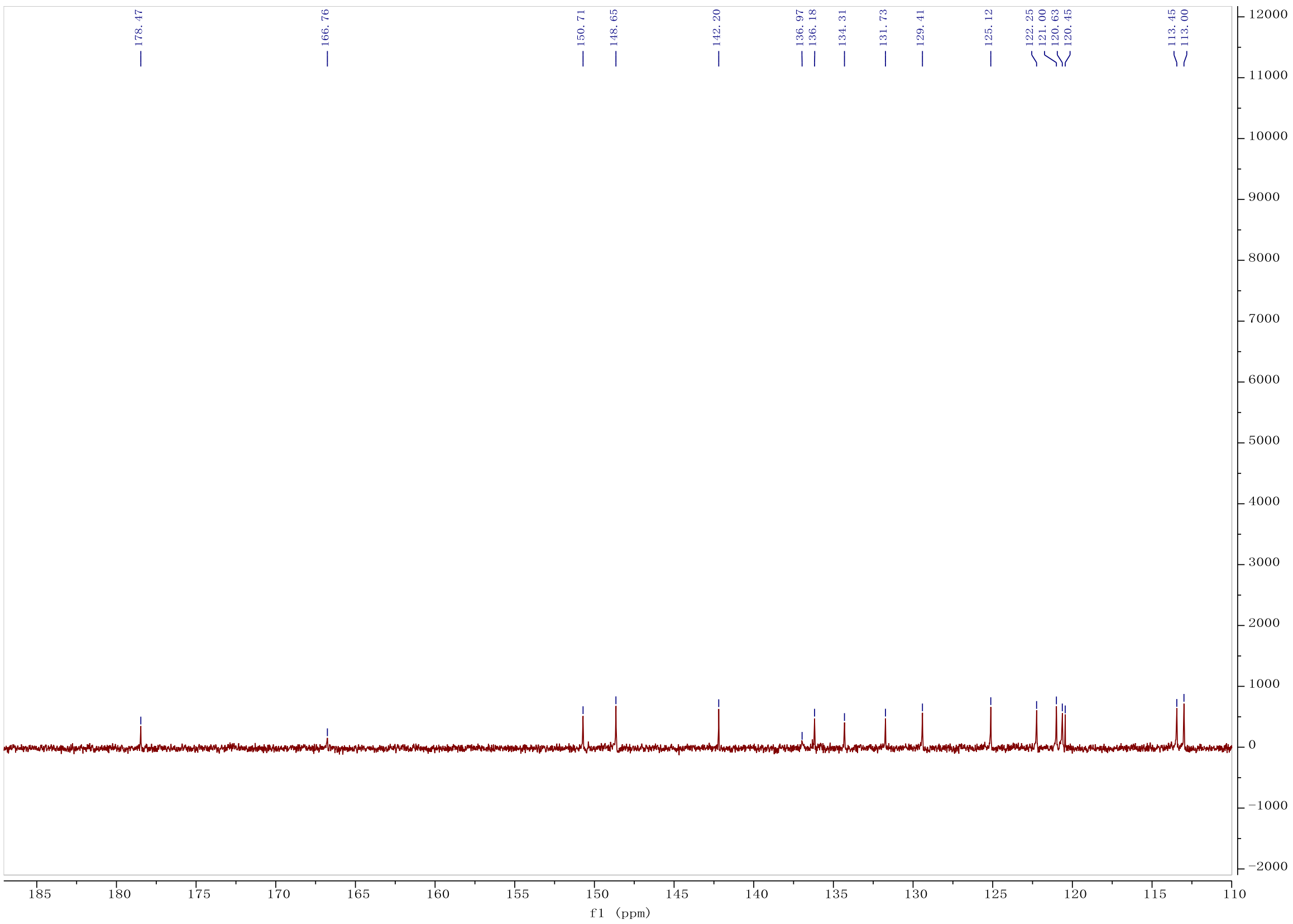

Figure S31 ^{13}C NMR (100MHz) spectrum of **3** in DMSO-d_6

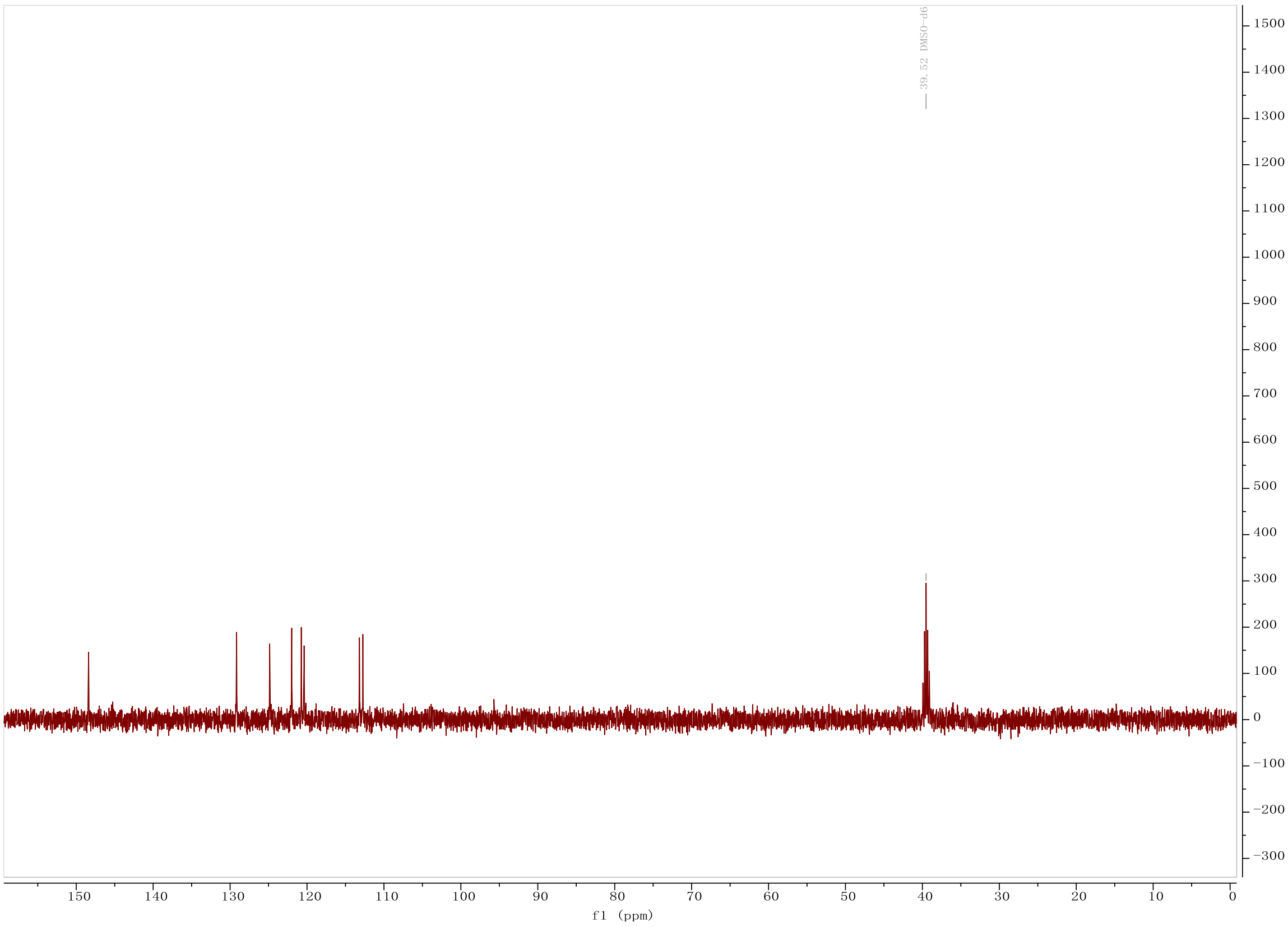

Figure S32 DEPT 135 (100MHz) spectrum of **3** in DMSO-d_6

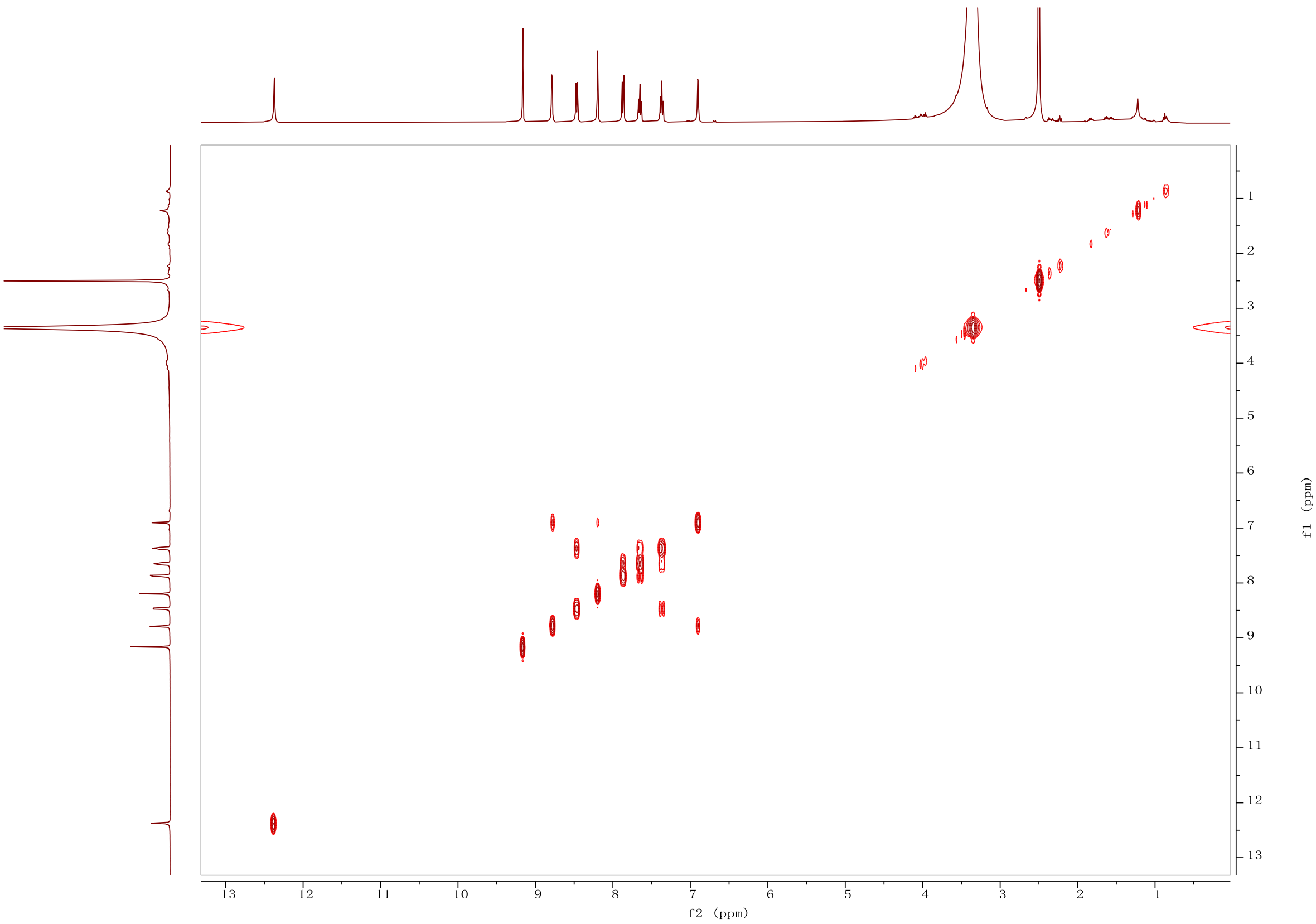

Figure S33 ^{1}H-^{1}H COSY spectrum of **3** in DMSO-d_6

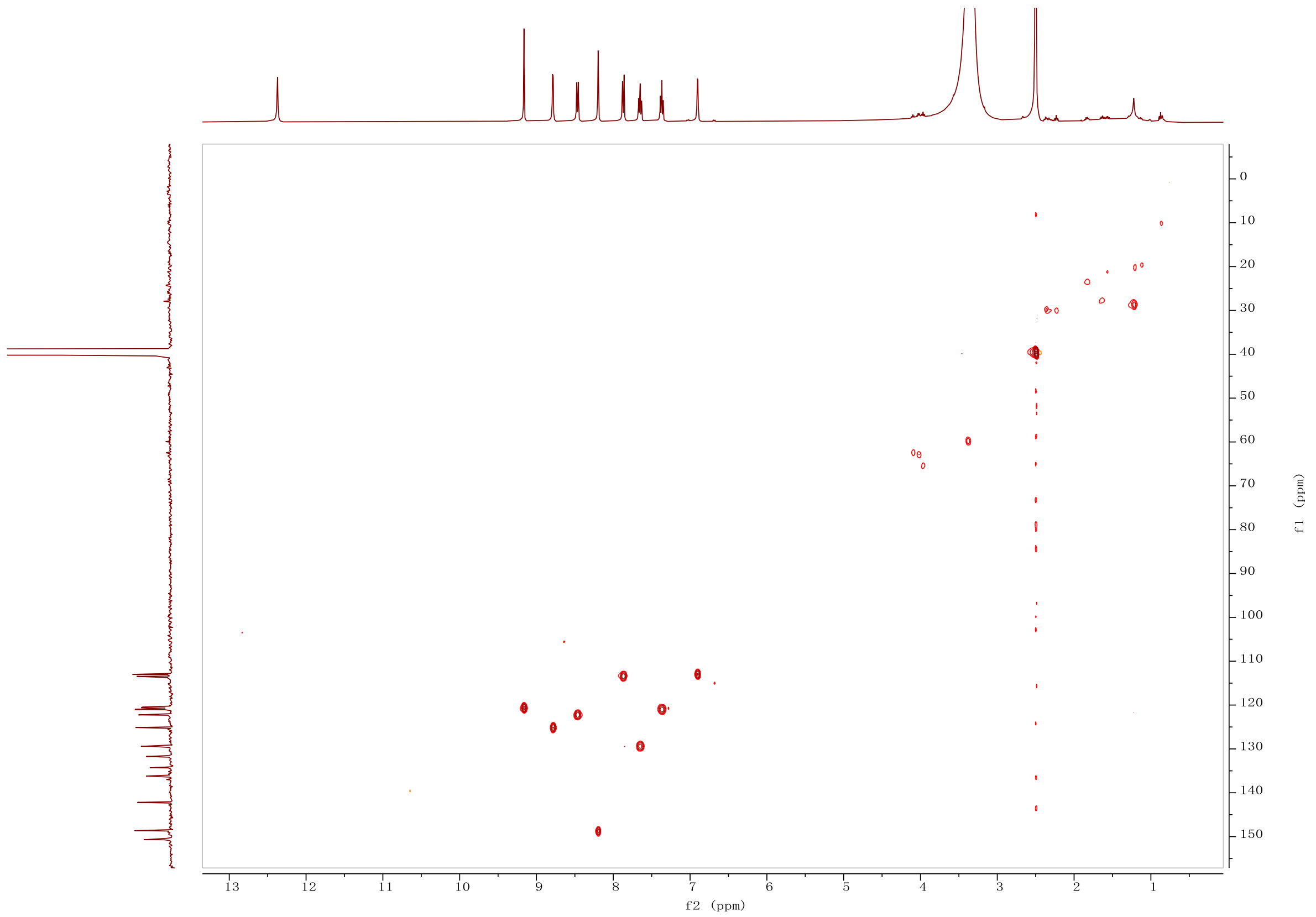

Figure S34 HSQC spectrum of **3** in DMSO-d_6

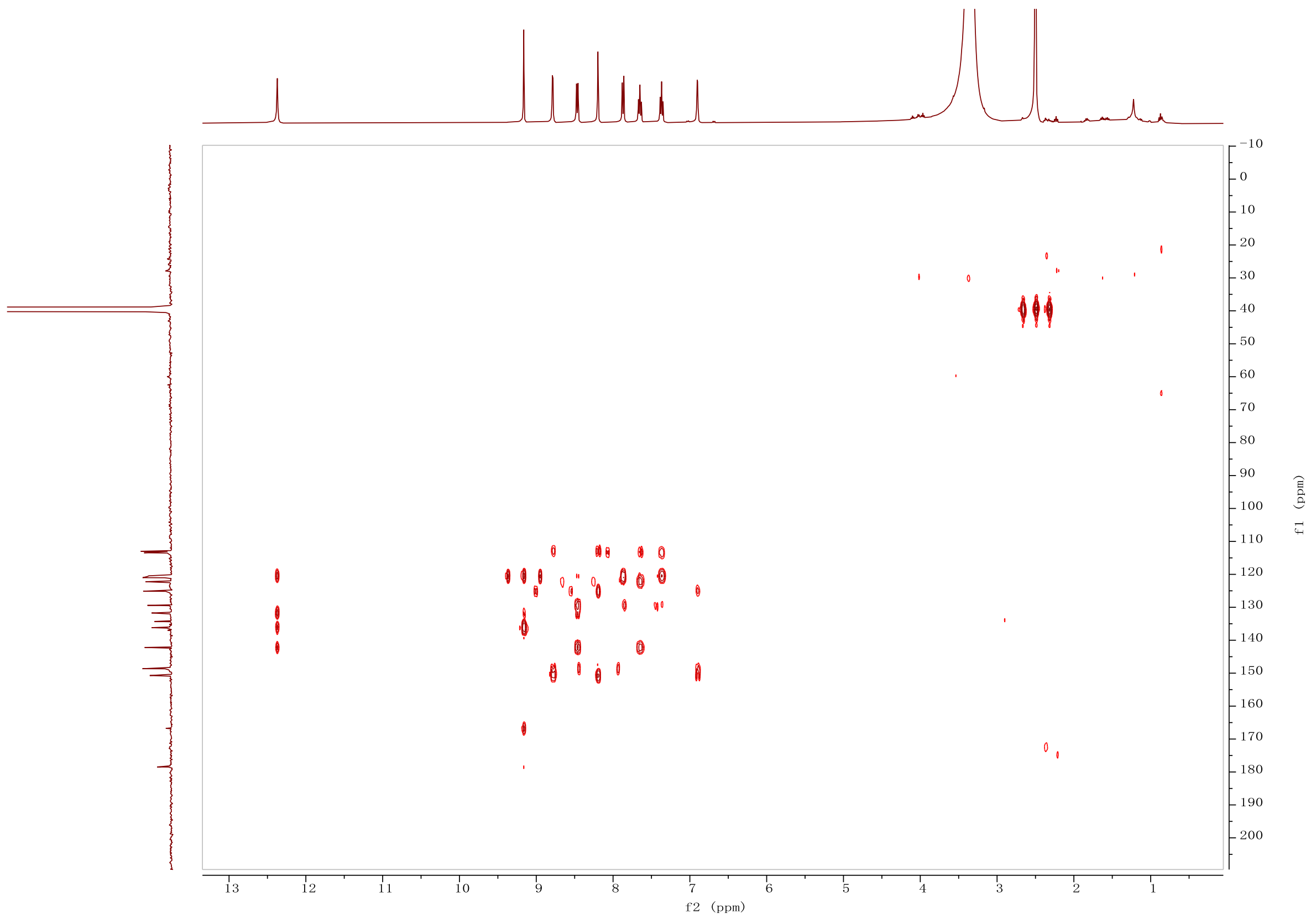

Figure S35 HMBC spectrum of **3** in DMSO-d_6

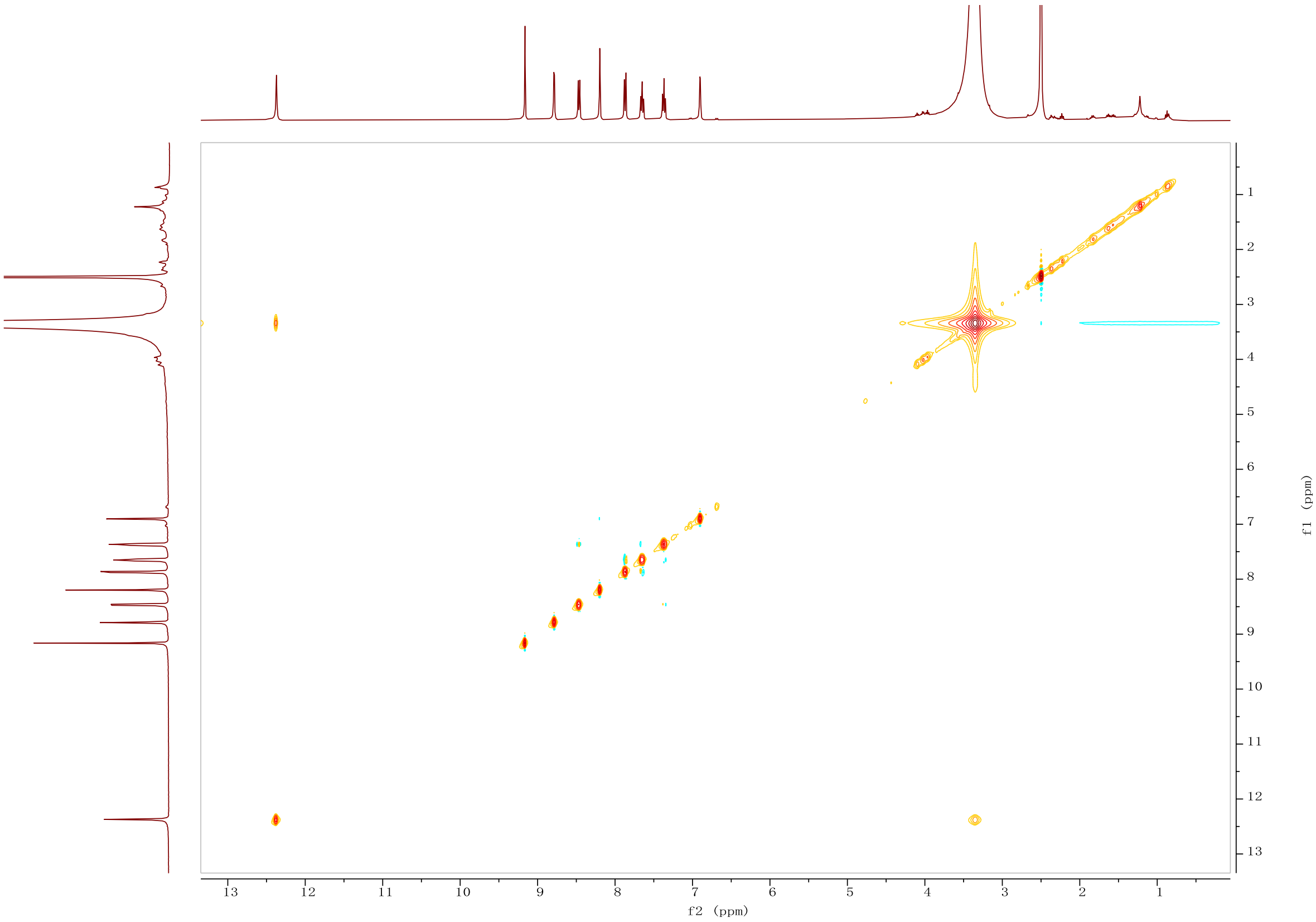

Figure S36 NOESY spectrum of **3** in DMSO-d_6

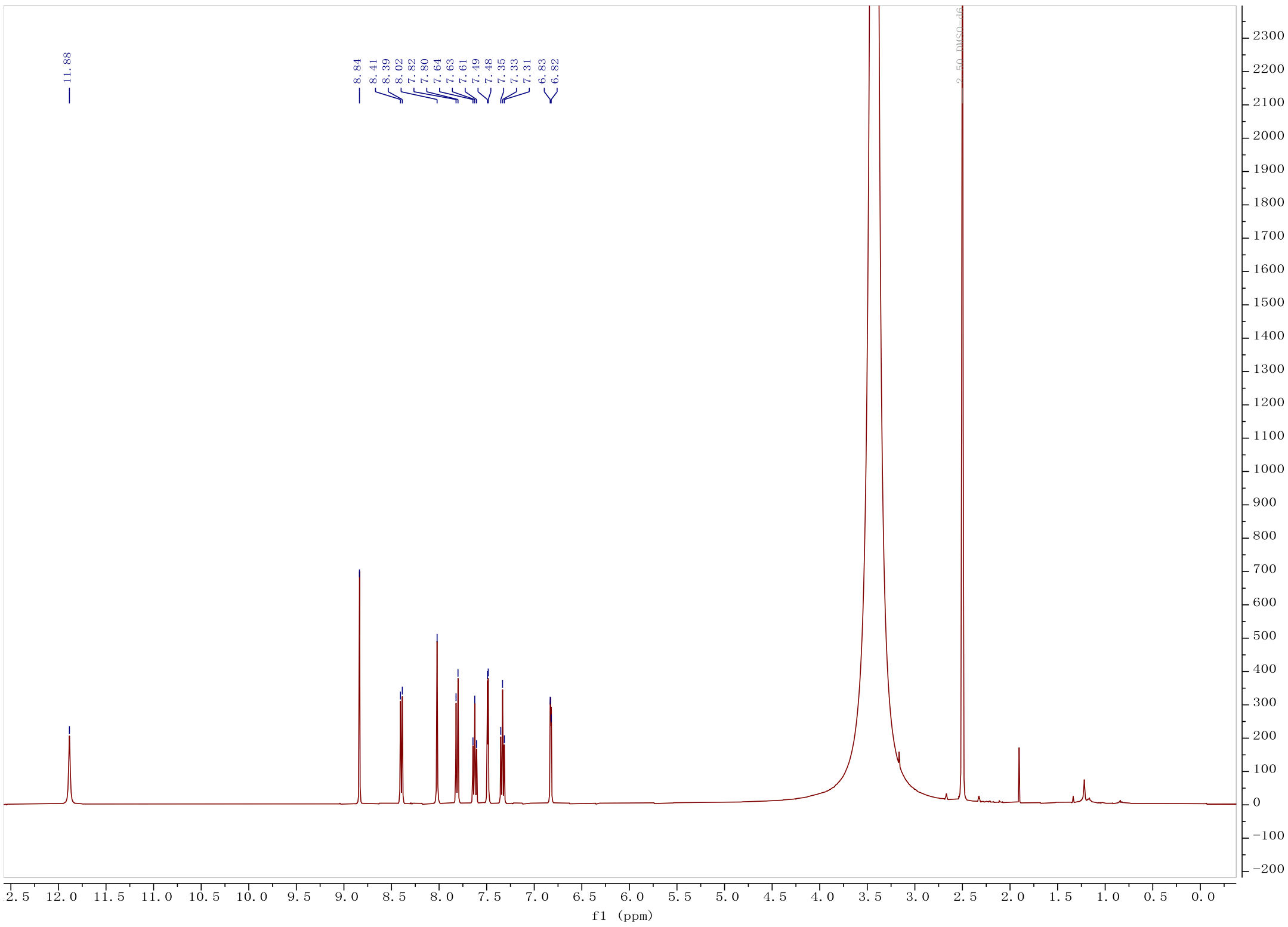

Figure S37 ^{1}H NMR (400MHz) spectrum of **4** in DMSO-d_6

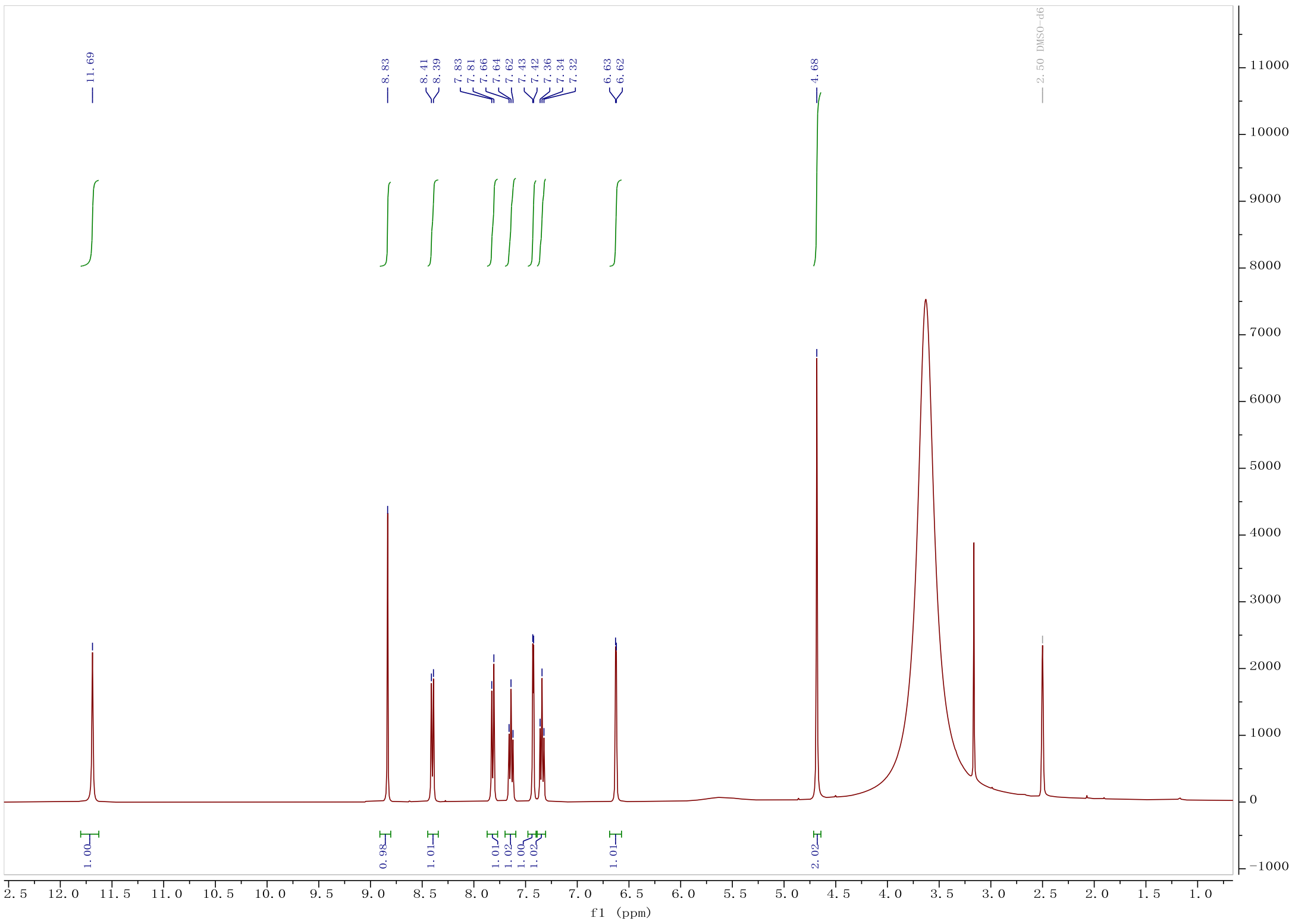

Figure S38 ^{1}H NMR (400MHz) spectrum of **5** in DMSO-d_6

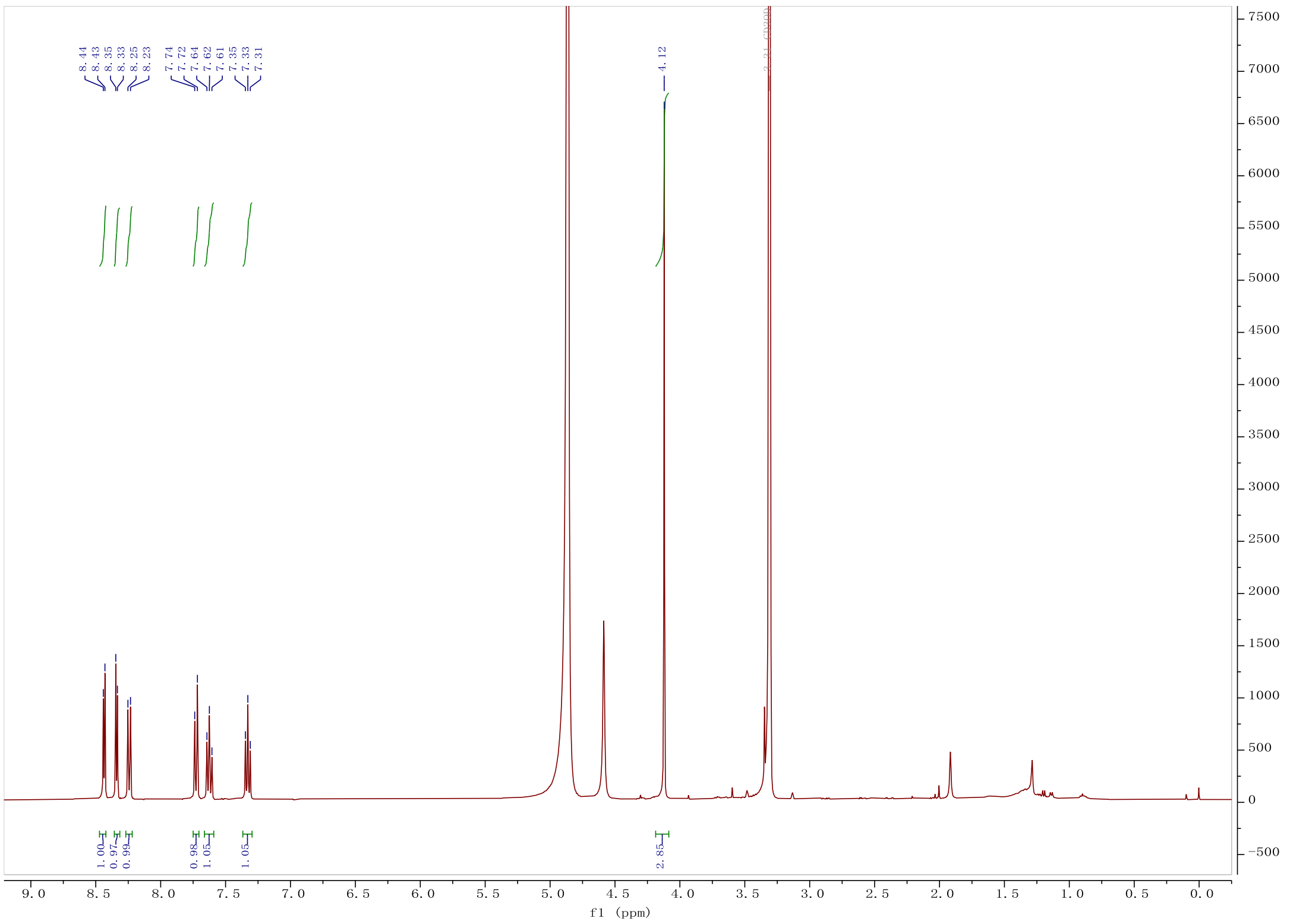

Figure S39 ^{1}H NMR (400MHz) spectrum of **6** in CD_3OD

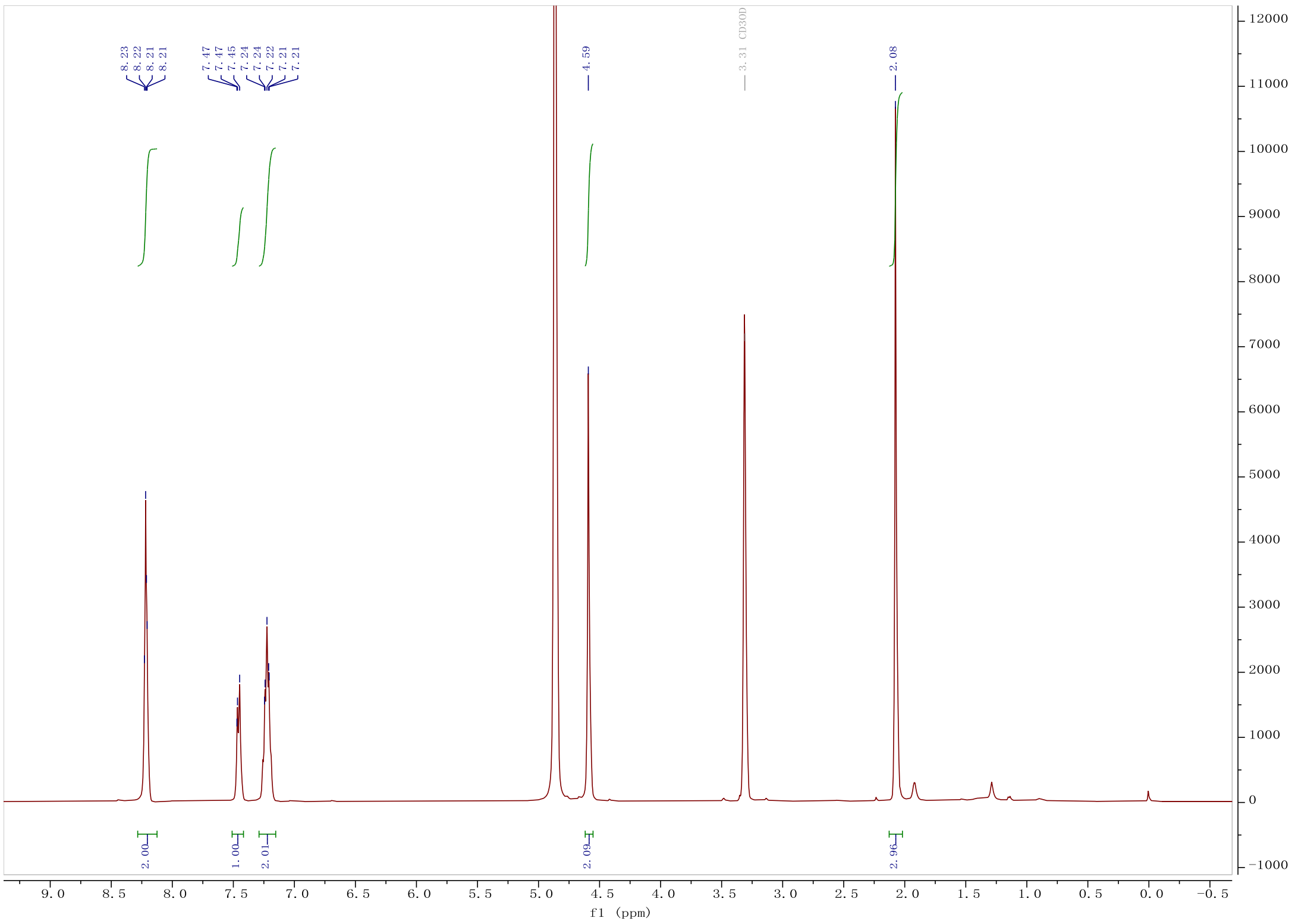

Figure S40 ^{1}H NMR (400MHz) spectrum of **7** in CD_3OD

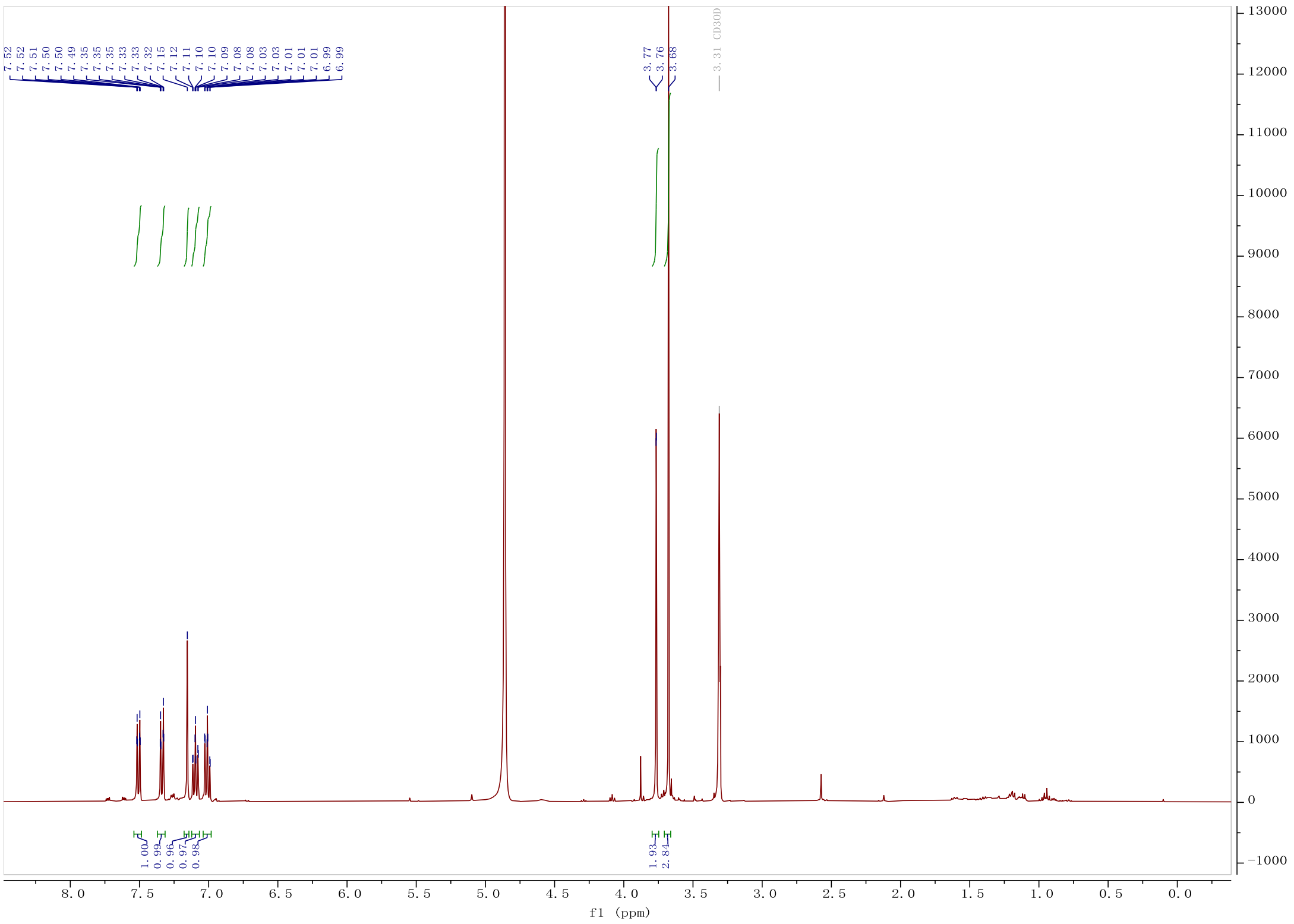

Figure S41 ^{1}H NMR (400MHz) spectrum of **8** in CD_3OD

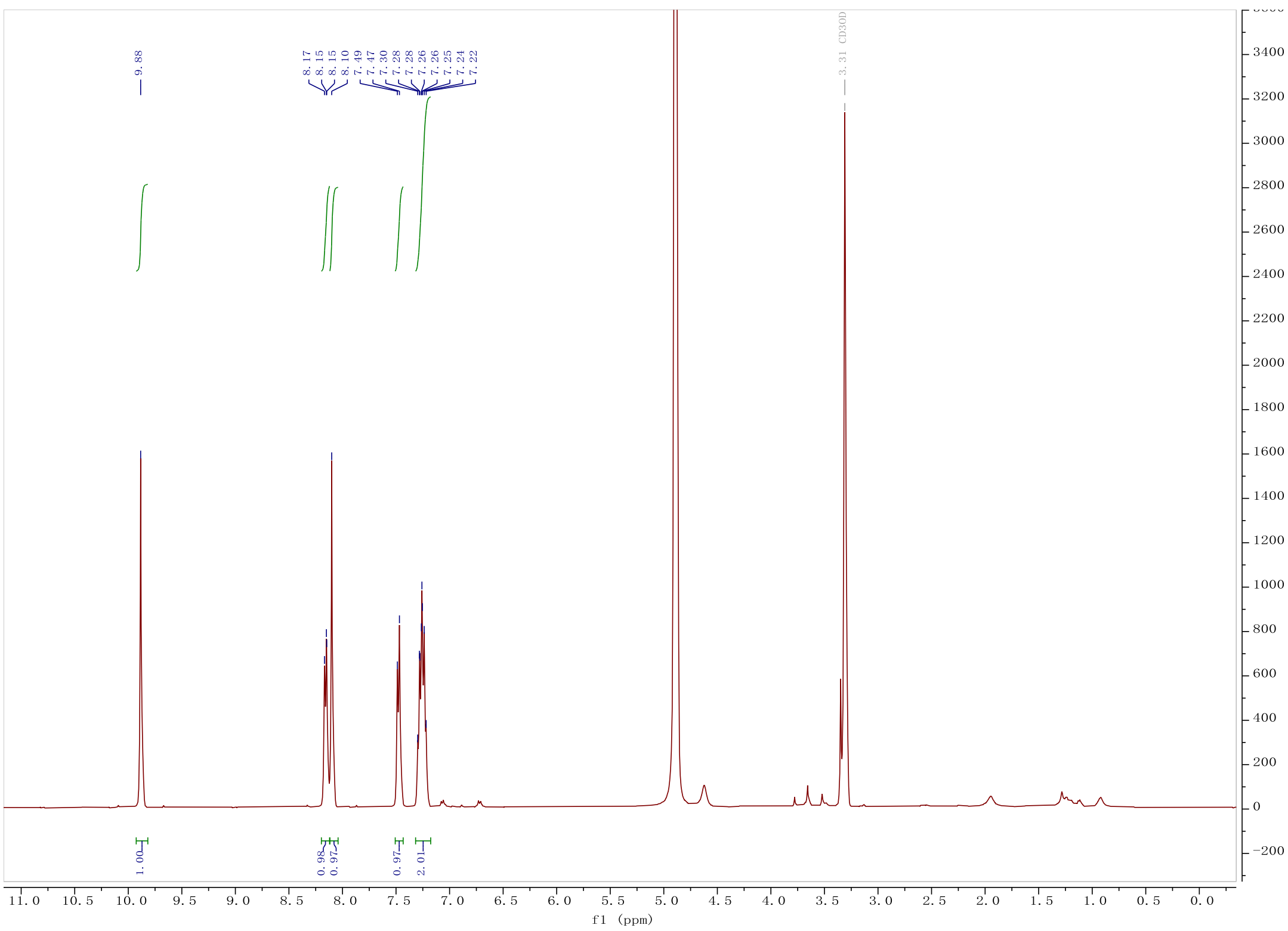

Figure S42 ^{1}H NMR (400MHz) spectrum of **9** in CD_3OD

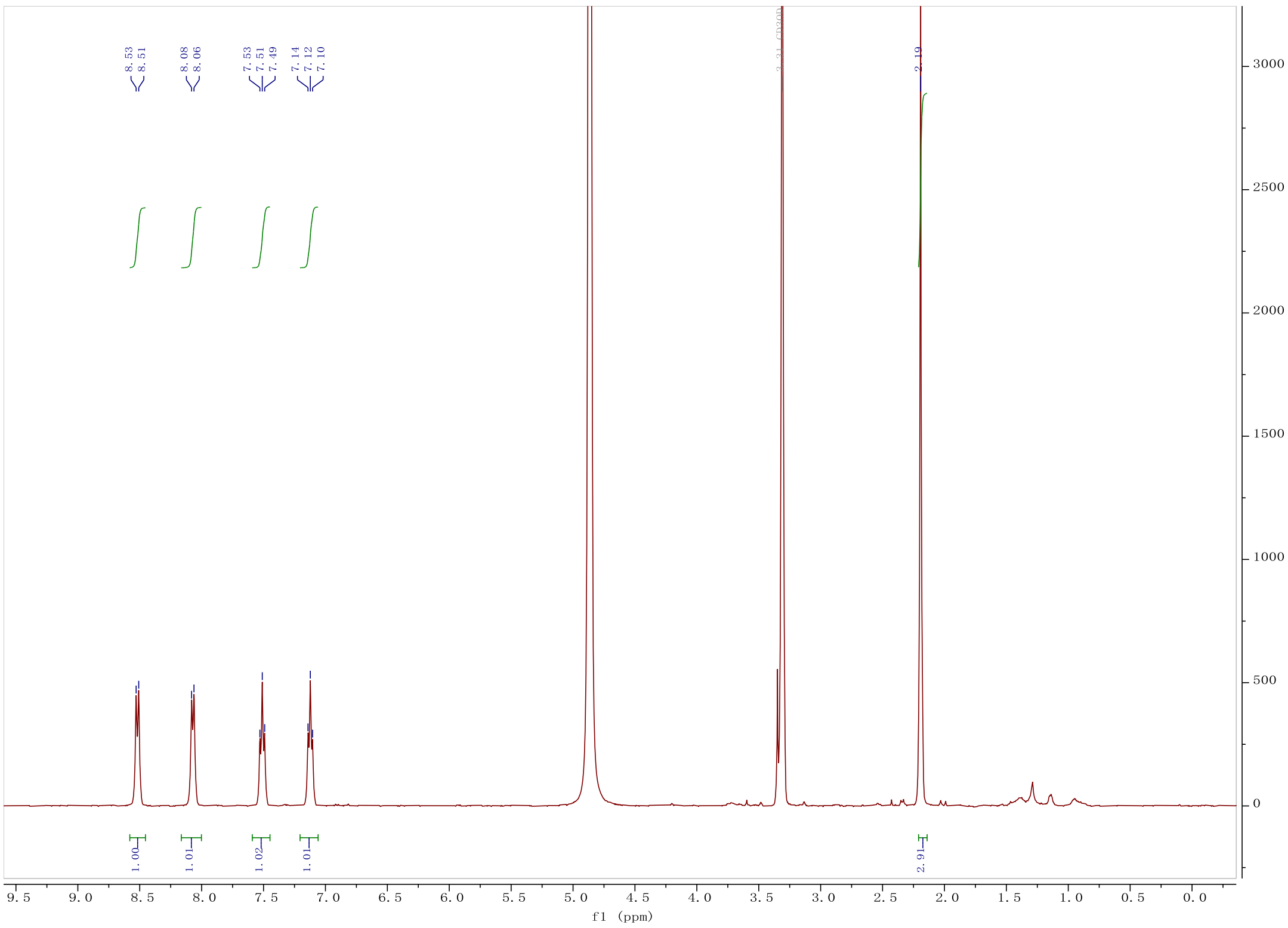

Figure S43 ^{1}H NMR (400MHz) spectrum of **10** in CD_3OD

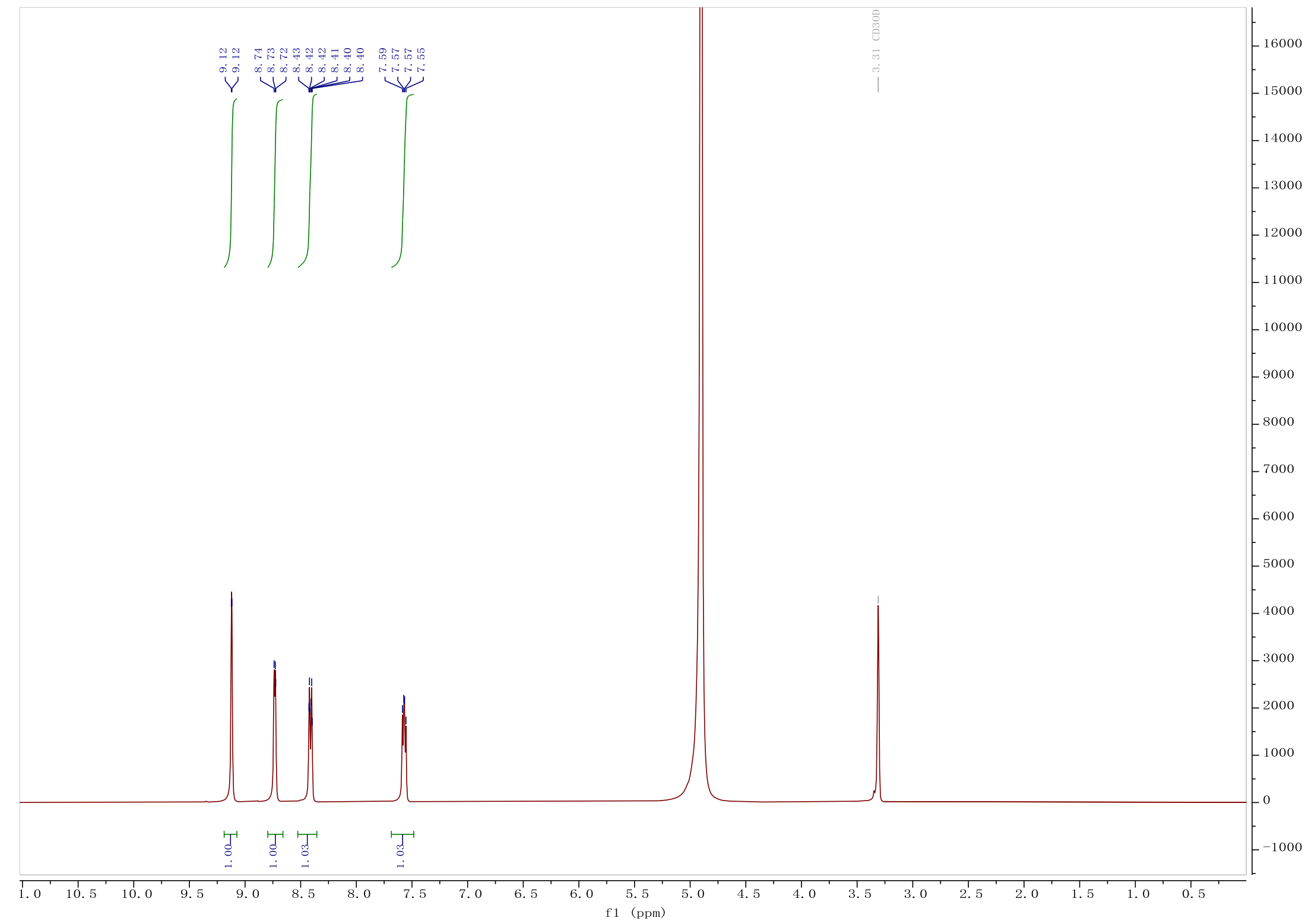

Figure S44 ^{1}H NMR (400MHz) spectrum of **11** in CD_3OD

References

1.Y. T. Lin, X. W. Yao, Z. W. Luo, et al, "Polyketides with Cardioprotective Bioactivities from Sponge-Associated Fungus *Aspergillus giganteus* MA46-5," *Molecules* 30(2025):1632. https://doi.org/10.3390/molecules30071632.

2.H. Fan, Z. M. Shi, Y. H. Lei, et al, "Rare Carbon-Bridged Citrinin Dimers from the Starfish-Derived Symbiotic Fungus *Penicillium* sp. GGF16-1-2," *Marine Drugs* 20(2022):443. https://doi.org/10.3390/md20070443.
